# Effect of Capacitation on Proteomic Profile and Mitochondrial Parameters of Spermatozoa in Bulls

**DOI:** 10.1021/acs.jproteome.4c00910

**Published:** 2025-03-25

**Authors:** María Castelló-Ruiz, Sabrina Gacem, Manuel M. Sánchez del Pino, Carlos O. Hidalgo, Carolina Tamargo, Manuel Álvarez-Rodríguez, Jesús L. Yániz, Miguel A. Silvestre

**Affiliations:** 1 Department of Cellular Biology, Functional Biology and Physical Anthropology, Universitat de València, Burjassot 46100, Spain; 2 Unidad Mixta de Investigación Cerebrovascular, Instituto de Investigación Sanitaria La Fe, Hospital Universitario y Politécnico La Fe, Valencia 46026, Spain; 3 Department of Biochemistry and Molecular Biology, Institute for Biotechnology and Biomedicine (BIOTECMED), 16781 University of Valencia, Burjassot 46100, Spain; 4 Animal Selection and Reproduction Area, Regional Agrifood Research and Development Service (SERIDA), Deva, Gijón 33394, Spain; 5 Department of Animal Reproduction, Spanish National Institute for Agricultural and Food Research and Technology (INIA-CSIC), Madrid 28040, Spain; 6 BIOFITER Research Group, Institute of Environmental Sciences (IUCA), University of Zaragoza, Huesca 22071, Spain

**Keywords:** spermatozoa, capacitation, proteome, mitochondria, bovine; flow cytometry

## Abstract

Sperm capacitation is a critical process for fertilization. This work aims to analyze the effect *in vitro* capacitation had on the proteome and mitochondrial parameters of bull spermatozoa. Viability, mitochondrial membrane potential (MMP), and reactive oxygen species (mROS) were assessed by flow cytometry in noncapacitated (NC) and *in vitro* capacitated (IVC) sperm. Proteome was evaluated using SWATH-MS. *In vitro* capacitation significantly induced a decrease in sperm viability, a high MMP, and an increase in mROS production. Within the group of living spermatozoa, the capacitation significantly induced a decrease in healthy mitochondrial spermatozoa, as well as an increase in mROS production, without affecting the MMP intensity. A total number of 72 differentially abundant proteins were found of which 63 were over-represented in the NC sperm group and 9 in the IVC sperm group. It was observed that many proteins associated with the sperm membrane and acrosome were lost during the capacitation process. For the IVC sperm, the functional enrichment was found in proteins related to the oxidative phosphorylation process. Our results indicate that the capacitation process induces a significant loss of seminal plasma-derived membrane proteins and a significant increase in proteins related with the oxidative phosphorylation (OXPHOS) pathway. Data are available via ProteomeXchange with identifiers PXD056424 and PXD042286.

## Graphical abstract

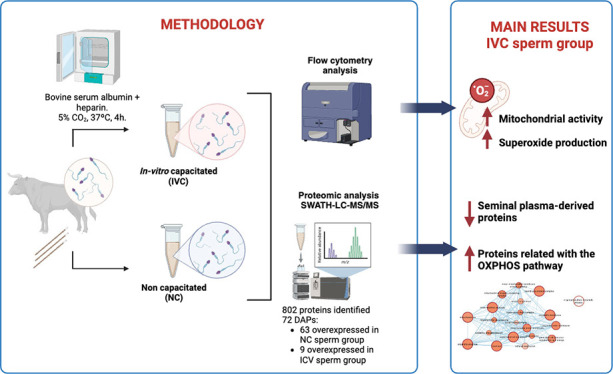

## 1 Introduction

The mammalian spermatozoon is one of the most specialized cells in the body.^1^ Following spermatogenesis, spermatozoa must undergo post-testicular maturation by passing through the epididymis and the female reproductive tract. It is within the latter environment that spermatozoa experience a series of biochemical modifications such as changes in the membrane’s lipid composition, including cholesterol loss and lipid packaging disorder, as well as a decrease in intracellular pH.^2,3^ These changes are accompanied by biophysical modifications such as the acrosome reaction and hyperactivity, collectively termed capacitation. Sperm capacitation is a complex process that has been studied in detail, including changes in membrane composition, calcium concentration, membrane hyperpolarization, intracellular pH (pHi), cyclic adenosine monophosphate (cAMP), and post-translational modifications.^4^ In mammal spermatozoa, the presence of bicarbonate and calcium in the external medium (oviductal or *in vitro* medium) triggers the activation of the adenylyl cyclase, and this activation increases cAMP and activation of protein kinase A (PKA).^2,5,6^ This activation of PKA regulates the protein tyrosine phosphorylation, which is related with plasma membrane potential hyperpolarization in human sperm.^5^ Supporting these ion gradients through modulation of pumps and ion transporters involves a considerable energy cost.^2^

During the capacitation process, controlled amounts of ROS are produced by the sperm, and these ROS are involved in the different steps of the process.^7^ Several studies have observed an increase in mitochondrial reactive ROS in sperm following an *in vitro* capacitation treatment.^8,9^ ROS in sperm present a dual role: they are essential for initiating capacitation through critical tyrosine phosphorylation events, but they can also be detrimental as spermatozoa are highly susceptible to oxidative damage due to their limited antioxidant defenses and the presence of highly oxidizable substrates, such as polyunsaturated fatty acids in membranes.^10^

However, there is limited information regarding the proteomic changes that occur during sperm capacitation, particularly in bovine species when compared to humans and other animals (human,^11–13^ mouse,^14,15^ porcine,^16–18^ ovine,^19^ and buffalo^20^). The protein repertoire involved in bovine sperm capacitation remains largely unexplored.

Therefore, this work aimed to study the effect that *in vitro* capacitation had on the proteome and mitochondrial parameters of bull spermatozoa.

## 2 Materials and Methods

### 2.1 Reagents and Media

Reagents for extension of sperm samples and the fluorescent stains used for the *in vitro* sperm evaluation were from Sigma-Aldrich (Merck Life Science S.L.U., Madrid, Spain). Dulbecco’s phosphate buffered saline (PBS; D8662) and Tyrode’s medium (TL) containing lactate and pyruvate were used for sperm dilution.^21^

### 2.2 Semen Collection

In a previous work,^21^ we analyzed several *in vitro* sperm parameters and proteome in bulls with different fertility. In the present work, frozen spermatozoa from the same bull semen samples were IVC so that we could compare the samples before (NC) and after IVC treatment. Bulls were housed in a center of artificial insemination (Cenero, Gijón, Spain). These males were commonly used commercially. Only ejaculates with sperm motility greater than 70% were chosen for the cryopreservation process. Then, ejaculates were diluted at room temperature (RT) with the Bioxcell diluent (IMV Technologies, L’Aigle, France) to a final sperm concentration of 23 × 10^6^ cells/mL and were frozen in 0.25 mL straws using a programmable freezer.^22^ After the cryopreservation procedure, quality control of the semen doses was performed at the artificial insemination center. For this, the progressive motilities of three randomly semen straws from each freezing batch were evaluated using a CASA system, with a cutoff value of over 40%.

### 2.3 Sperm Sample Preparation and *In Vitro* Capacitation

Three frozen straws per each bull were used in the experiment. Sperm cells from the same sample from three straws per male were used for both cytometry and proteomics analyses. Ten Holstein-Friesian bulls were used in the experiment. The frozen straws were thawed in a water bath at 37 °C for 1 min and were pooled and centrifuged at 956*g* for 15 min at RT on a Percoll monolayer gradient (45% in TL [v/v], Percoll; P4937) to separate the spermatozoa from other putative cells and debris.^23^ The sperm pellets were extended in TL and centrifuged again at 956*g* for 5 min at RT. Each male sample was split: one part was used to assess sperm parameters by cytometry and proteomic profile before IVC, and the second part was submitted to the IVC procedure and assessed later with the same protocols. For IVC, spermatozoa were incubated in TL supplemented with 6 g/L bovine serum albumin (BSA; A7906) and heparin (H9399; 10 IU/ml) at 5% CO_2_ at 37 °C for 4 h.^22^ For proteomic analysis, the samples were washed five times in PBS, and the resulting pellet was stored at −20 °C until further analysis.

### 2.4 Viability, MMP, and ROS of Spermatozoa Assessment

Viability, MMP, mitochondrial ROS (mROS), and IVC of spermatozoa were assessed by using flow cytometry (FC) in the Central Service for Experimental Research (SCSIE) at the University of Valencia. Multiparametric FC analyses were performed using a BD LSRFortessa flow cytometer equipped with five lasers emitting UV wavelengths at 355 nm, blue at 488 nm, yellow-green at 561 nm, violet at 405 nm, and red at 640 nm. The system was controlled by using the FACSDiva 8 software. A minimum of 7500 cells per replicate were recorded, and flow rate was maintained at 500–1500 cells/s.

To evaluate sperm viability (plasma membrane integrity), stain 4’,6’-diamidino-2-phenylindole (DAPI; D9542; 1 μg/mL) was used. DAPI-positive and DAPI-negative cells were considered as dead and living cells, respectively.^24,25^ To evaluate sperm capacitation, merocyanine 540 (M540; 323756; 2 μM) was used to evaluate plasma membrane fluidity.^26,27^ M540-positive and M540-negative cells were considered as IVC and NC sperm, respectively. MMP was assessed by using Mitotracker Deep Red (MTDR; M22426; Invitrogen, 100 nM). MTDR-positive and MTDR-negative cells were considered as sperm with high and low MMP, respectively.^26^ To study the mROS (superoxide anion radical) produced by mitochondria, MitosoxTM Red (MSOX; M36008; Invitrogen, 1 μM) was used. MSOX-positive and MSOX-negative cells were considered as sperm with high and low amounts of superoxide, respectively.^28^ Sperm samples, approximately 5 million spermatozoa/mL, were incubated jointly with DAPI, MTDR and MSOX, or DAPI and M540 for 15 min at 37 °C in the dark. After incubation, the samples were washed and analyzed by FC. DAPI was detected with peak excitation at 355 nm and emission at 450 and 40 nm BP. M540 was detected with a peak excitation at 555 nm and emission at 578 nm. MTDR was detected as peak excitation at 640 nm and emission at 670/14 nm. MSOX was detected at peak excitation 561 nm and emission at 610/20 nm.

### 2.5 Sperm Proteomics

The proteomics analyses were carried out in the Proteomics Unit at the University of Valencia.

#### 2.5.1 Sample Preparation for Protein Extraction and the Spectral Library Building

Protein extraction and quantification and the building of a spectral library were performed following Gacem et al.^21^ Briefly, a Laemmli buffer (Bio-Rad, Hercules, CA, United States) was used to extract protein from sperm samples and was afterward quantified using a Macherey-Nagel quantification reagent (Macherey-Nagel, Düren, Germany). To build a spectral library, a 1D SDS-PAGE was run with 2.5 μg/sample of all samples. The gel lane was sliced into four pieces that were reduced for 30 min at 60 °C in DTT and digested overnight at 37 °C with 250 ng of trypsin (Promega, Madison, WI, United States).^29^ Then, a single gel band containing all proteins from individual samples for both groups (*n* = 10 for NC and *n* = 10 for IVC group) was prepared following the same protocol described above for further Sequential Window Acquisition of All Theoretical Spectra (SWATH) analysis.^21^

#### 2.5.2 Liquid Chromatography and Tandem Mass Spectrometry (LC-MS/MS) Analyses and SWATH Analysis of Individual Samples

5 μL of the digested peptide mixture from each gel slice was analyzed by LC using an Ekspert nanoLC 425 (Eksigent Technologies, Dublin, CA, USA) connected to a mass spectrometer nanoESI qQTOF 6600 plus TripleTOF (SCIEX, Framingham, MA, USA), following the procedure described by Perez-Patiño et al.^30^ with minor modifications in the elution gradients.^21^ For the SWATH LC-MS/MS analysis, digested samples were individually analyzed by operating the TripleTOF 6600plus in SWATH mode.

#### 2.5.3 Protein Identification and Quantification

To generate the peptide library, the wiff files from datadependent acquisition experiments were used for protein identification. The paragon algorithm^31^ of ProteinPilot v5.0 (SCIEX, Framingham, MA, USA) was used to search the B. taurus database with the following settings: trypsin specificity, IAM cys-alkylation, taxonomy not restricted, and the search effort set to through and FDR correction. The identified proteins were grouped using the ProteinPilot Group algorithm. Protein quantitation was performed with PeakView (version 2.2, SCIEX, Framingham, MA, USA), and the wiff files from the data-independent acquisition (SWATH) experiments were scanned with the peptide library created. The sum of the areas under the curves of extracted ion chromatograms that underwent six transitions of up to 20 peptides per protein was used to quantify the proteins. Only peptides with confidence over 95% and a false discovery rate (FDR) below 1% were used.^21,23^ Samples were normalized by their total protein abundance. Data from MS proteomics have been deposited to the ProteomeXchange Consortium via the PRIDE partner repository with the data set identifier PXD056424 and PXD042286.

### 2.6 Statistical Analyses

#### 2.6.1 Analysis to Evaluate Sperm Viability, MMP, MROS, IVC Rate, and MH Rate

The Kolmogorov–Smirnov test was performed to assess the normality of the variables. Before statistical analysis, the variables that did not present normality were transformed by calculating the arcsine of the square root or the logarithm for percentages or continuous variables, respectively. The variables were analyzed using Generalized Linear Models (GLMs) with the SPSS Statistics v.28 program (IBM, Corp; Armonk, NY). Model with one factor (NC vs IC) was used for sperm parameters. A probability of *p* ≤ 0.05 was considered statistically different.

#### 2.6.2 Proteomic Data Analysis of Sperm

The proteomics data analyses were carried out in the Statistics and Omics Data Analysis Section at the University of Valencia. As the capacitation medium contained a considerable amount of BSA, changes in albumin protein levels were not considered in the study as it appeared as a differential protein in the IVC group, despite the washing process.^12^ Following Gacem et al.,^21^ three statistical methodologies were applied. Results from identified proteins were transformed by calculating log base 2 and analyzed by a logistic regression model with Elastic Net penalty (ENLR), using the glmnet R package (version 4.1–2) with a train function of the caret package (lambda = 0.433; alpha = 0.1). To analyze data variation, a supervised classification analysis (see partial least-squares-discriminant analysis graphic) with the R package mixOmics (version 6.16.3) was performed. Those proteins analyzed by ENLR that have a VIP (variable importance in projection) function greater than 1.5 were selected. Finally, we analyzed all proteins using the Limma package for R software (version 3.48.3) and by making multiple comparisons with Benjamini and Hochberg. Proteins with an adjusted *p* value ≤ 0.05 were selected. A combination of the three methodologies was used to obtain a list of relevant differentially abundant proteins (DAPs) between NC and IVC sperm samples.

#### 2.6.3 Bioinformatic Analysis

The functional enrichment analysis was performed using g:Profiler (https://biit.cs.ut.ee/gprofiler/), Cytoscape (https://cytoscape.org), and ClusterProfiler (https://bioconductor.org/packages/release/bioc/html/clusterProfiler.html). In order to analyze the over-representation of all DAPs, we used g:GOSt with the options B. taurus, all proteins known, and the g_SCS significance threshold method (0.05). All the terms of Gene Ontology (GO) with an FDR ≤ 0.01 were categorized into three classes: (1) molecular process (MP), (2) cellular component (CC), and (3) biological process (BP). Cystoscape was used to visualize interactions of biological pathways from the enrichment analysis. Additionally, we performed a Gene Set Enrichment Analysis (GSEA) using the ClusterProfiler package of all the 802 proteins obtained, ordered by the fold change obtained with the Limma package. Analyses of the potential protein–protein interactions of the DAPs were obtained using the Search Tool for the Retrieval of Interacting Genes (STRING) software (https://string-db.org). The queries were made with the B. taurus database (access date: sixth July 2022). List of unrecognized DAPs for B. indicus × B. taurus (hybrid) were mapped to find orthologous proteins for B. taurus using the g:Orth option and Uniprot Blast.

## 3 Results

### 3.1 Cytometric Analysis of Sperm Quality and Capacitation Status

Several sperm parameters (viability, capacitation, MMP, and mROS) were evaluated by FC before and after IVC treatment (Table 1). IVC significantly decreased sperm viability and induced high MMP, as well as significantly increased the production of mROS. In the living spermatozoa group, we evaluated both the percentage of capacitation and mitochondrial healthy (MH) spermatozoa (live sperm with high MMP and low mROS), as well as the MMP and mROS intensity (Figure 1).

**Table 1:** Viability, Capacitation Status, MMP, and mROS of Non- and IVC Bull Spermatozoa

sperm parameters^a^	noncapacitated	IVC
viability (%)	44.01 ± 3.4	13.47 ± 1.32^b^
capacitated (% from live sperm)	1.76 ± 0.39	67.93 ± 6.45^b^
high mROS (%)	43.27 ± 3.70	78.28 ± 3.48^b^
high MMP (%)	53.40 ± 3.44	11.41 ± 1.37^b^
MH (% from live sperm)	97.22 ± 0.44	61.11 ± 4.62^b^
MMP intensity of live sperm	10520.30 ± 1612.57	7483.20 ± 1437.17
mROS intensity of live sperm	584.40 ± 150.83	1174.70 ± 269.71^b^

a mROS: mitochondrial reactive oxygen species; MMP: mitochondrial membrane potential; MH spermatozoa (live sperm with high MMP and low mROS).

b Significant differences (*p* ≤ 0.05) among columns for sperm parameters.

**Figure 1:**
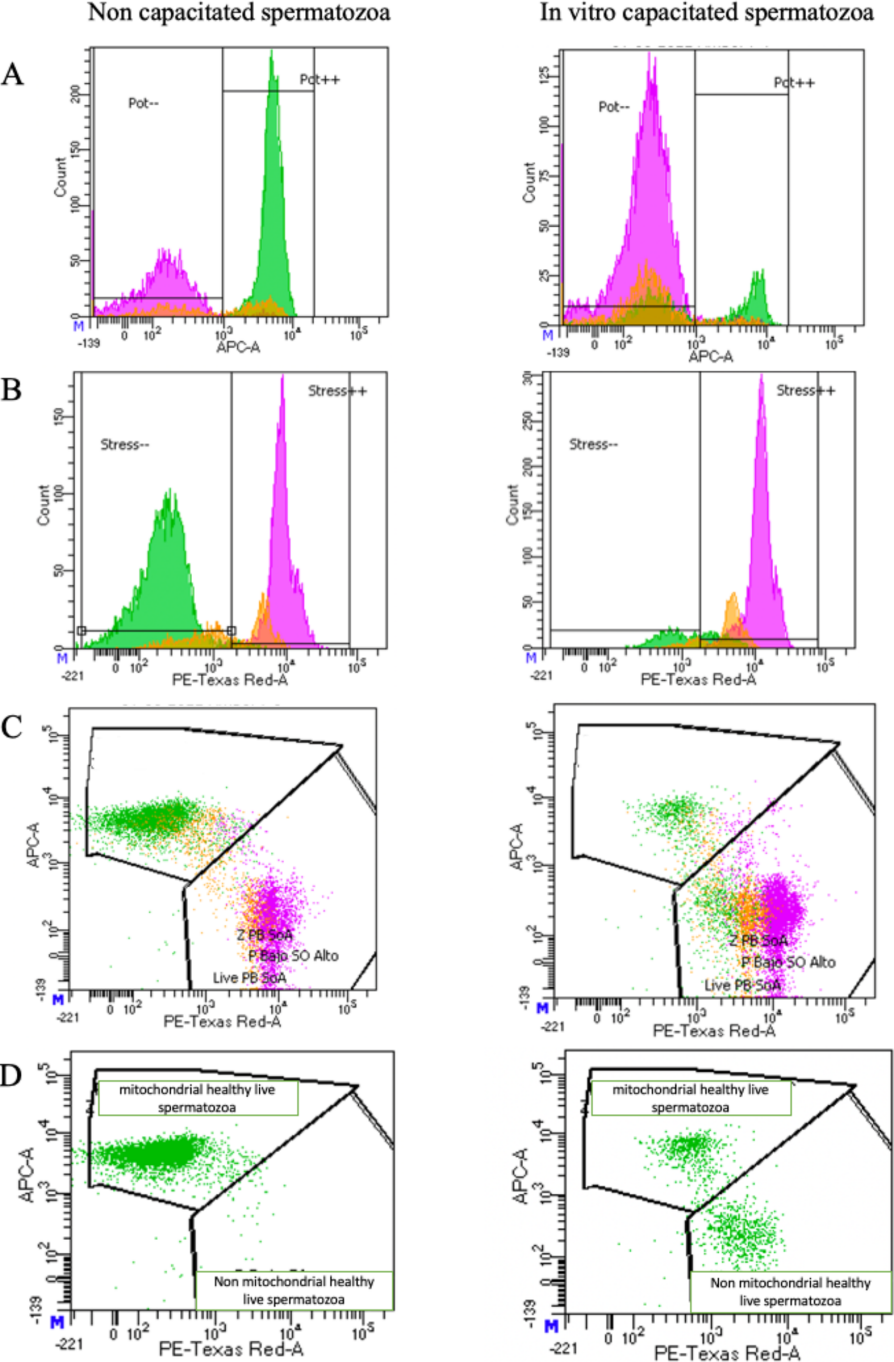
Example of FC output of MMP, mROS of NC, or IVC spermatozoa of one bull. Live, compromised, and dead spermatozoa are colored in green, orange, and pink, respectively. (A) Mitotracker Deep Red positive cells (Pot++) were considered as sperm with high MMP; (B) Mitosox Red positive cells (Stress+) were considered as sperm with high amounts of superoxide (mROS). (C,D) MH live sperm; live sperm with hMMP and low mROS.

A capacitation rate of 68% of live spermatozoa in the IVC group compared to 2% in the NC group was achieved. The capacitation process induced a significant decrease in MH spermatozoa and significantly increased the production of mROS, without affecting the high MMP.

### 3.2 Quantitative Proteomic Differential Analysis

SWATH-MS-MS proteomic analysis from IVC and NC sperm samples reveals a total amount of 802 proteins (Supplementary Table S1), which were aligned to the B. taurus proteome database. After performing an analysis of the proteins with a VIP > 1.5 in the PLS-DA classification, we assessed that the selected proteins, which were specifically grouped into two main clusters where differences in quantification were observed depending on whether they were in the IVC or NC group (Figure 2).

**Figure 2:**
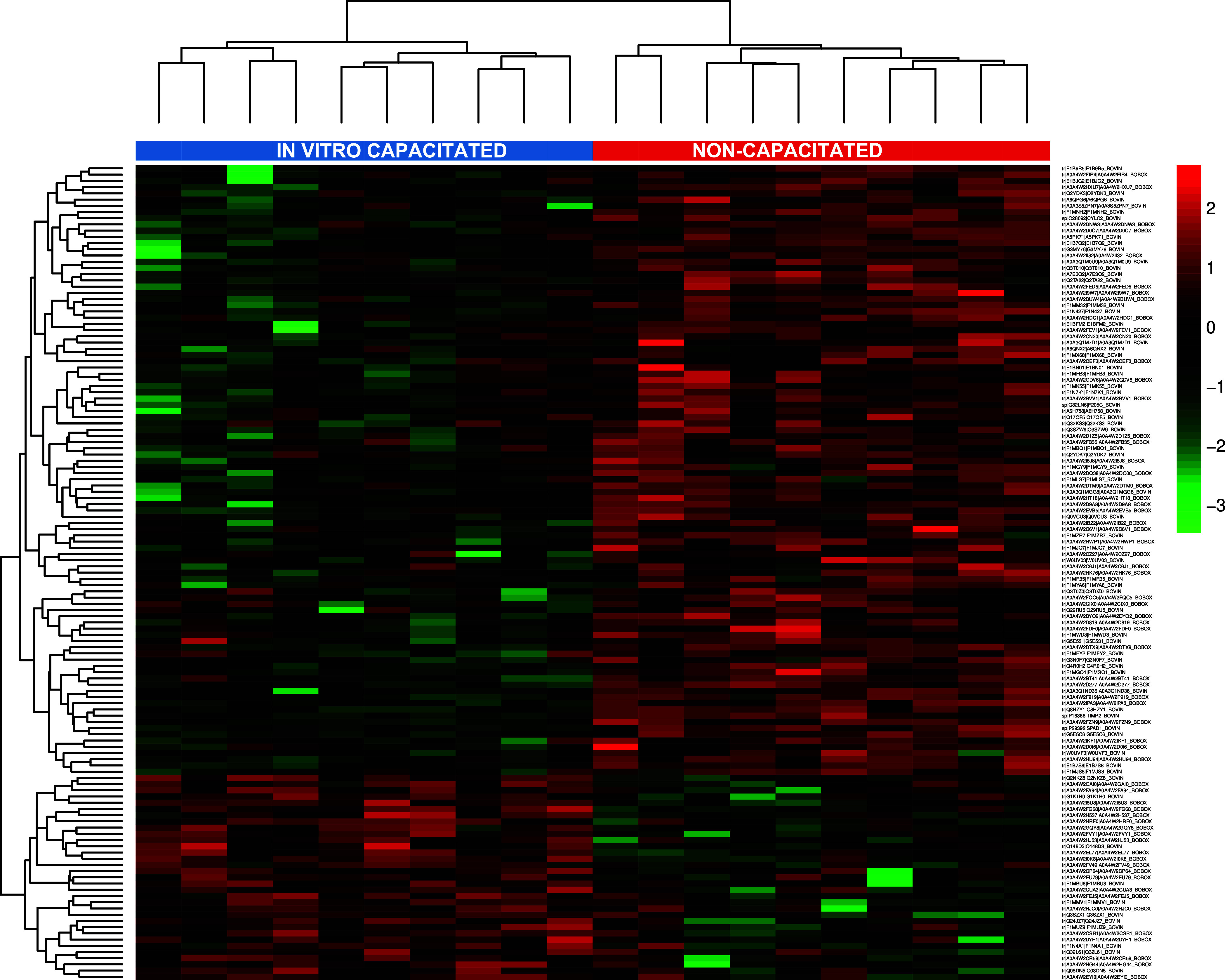
Heatmap of differentially expressed sperm proteins with a VIP > 1.5 in the PLS-DA classification. Heatmap analysis reveals that the sequence of proteins was differentially expressed in the IVC (blue samples) and NC (red samples) sperm groups.

Statistical analysis of the proteomic data revealed 72 DAPs, of which 63 were differentially over-represented in sperm from the NC group and 9 differentially over-represented in sperm from the IVC group (Tables 2 and 3).

**Table 2:** List of the Differentially Expressed Proteins in the Noncapacitated Sperm Group^a^

accession number	protein name	gene name	mass (KDa)	Log FC	adjusted *p* value
P16368	metalloproteinase inhibitor 2	TIMP2	24	2.680	6.8646 × 10^–07^
Q8HZY1	serine-protease inhibitor clade E member 2	SERPINE2	44	2.662	2.6878 × 10^–06^
Q4R0H2	spermadhesin-2	spadh2	15	2.099	1.1388 × 10^–05^
P29392	spermadhesin-1	SPADH1	15	1.255	1.5733 × 10^–05^
P17697	clusterin	CLU	51	1.935	3.2992 × 10^–05^
A0A452DJ99	C-type natriuretic peptide	NPPC	13	2.482	6.0322 × 10^–05^
F1MGQ1	deoxyribonuclease	DNASE1L3	35	1.980	0.00013993
Q28092	cylicin-2	CYLC2	54	1.055	0.00043123
Q8SPU5	BPI fold-containing family A member 1	BPIFA1	27	3.051	0.00048336
A0JNP2	secretoglobin family 1D member	SCGB1D	11	2.884	0.00052596
G5E5C6	acrosin	ACR	45	0.561	0.00073373
A0A3Q1MB60	uncharacterized protein	-	41	0.835	0.00076997
A0A3Q1LUP8	vesicle-associated membrane protein-associated protein A	VAPA	34	0.935	0.00130631
E1B7S8	acrosin-binding protein	ACRBP	61	0.618	0.00130631
A0A3Q1M858	lipocalin/cytosolic fatty-acid binding domain-containing protein	LOC100295548	20	1.654	0.00150306
G3MY76	cylicin-1	CYLC1	74	1.877	0.00185224
Q32LB5	GLIPR1-like protein 1	GLIPR1L1	27	0.821	0.00208318
A0A3Q1ND36	disintegrin and metalloproteinase domain-containing protein 1a-like	-	80	1.669	0.00229041
F1MM32	sulfhydryl oxidase	QSOX1	67	0.804	0.00348238
F1MF73	chromosome 3 C1orf56 homologue	C3H1orf56	33	0.606	0.00354176
F1MZR7	spermatid maturation 1	SPEM1	35	1.479	0.00488762
Q9N2I2	plasma serine-protease inhibitor	SERPINA5	45	1.556	0.00502487
F1N427	Septin	SEPTIN12	41	0.658	0.00502487
E1B7Q2	adenylate kinase 7	AK7	83	0.816	0.00677055
E1BGK2	leucine-rich repeat containing 74A	LRRC74A	54	0.694	0.00788506
A0A3Q1MGG8	IQ motif containing N	-	342	0.592	0.01035334
A7E3Q2	heat shock-related 70 kDa protein 2	HSPA2	70	0.586	0.01211339
A0A3Q1M0U9	transitional endoplasmic reticulum ATPase	VCP	89	0.639	0.01318627
Q32LN6	protein SPATA31F3	FAM205C	45	0.679	0.01395473
Q2YDK3	hyaluronidase	SPAM1	62	0.682	0.01594206
F1MK55	dynein axonemal heavy chain 2	DNAH2	508	0.363	0.01604564
Q32L61	calcium-binding tyrosine phosphorylation regulated	CABYR	49	0.742	0.01742493
P00514	cAMP-dependent protein kinase type I-alpha regulatory subunit	PRKAR1A	43	0.768	0.01742493
Q2TA22	long-chain-fatty-acid--CoA ligase	ACSL6	78	0.543	0.01742493
A5PK71	parkin coregulated gene protein	PACRG	29	0.491	0.01742493
F1N7K1	ropporin-1-like protein	ROPN1L	24	0.456	0.01742493
Q3SZW9	DnaJ (Hsp40) related; subfamily B. member 13	DNAJB13	36	0.493	0.01768009
E1BLG0	adenylate kinase 8	AK8	52	0.589	0.01839726
F1MGY9	aspartylglucosaminidase	AGA	37	0.633	0.02176345
Q3SYS7	IQ domain-containing protein F1	IQCF1	24	0.862	0.02845836
P63103	14–3–3 protein zeta/delta	YWHAZ	28	0.447	0.02845836
A0A3Q1MUT5	renin receptor	ATP6AP2	40	1.730	0.02847853
A8E4N3	radial spoke head protein 3 homologue	RSPH3	69	0.865	0.02847853
A0A3S5ZPN7	coiled-coil domain-containing 116	CCDC116	75	0.764	0.02847853
Q2TA43	actin-related protein T2	ACTRT2	42	0.404	0.02847853
F1MX68	carboxypeptidase	CPVL	55	0.469	0.03202207
E1B9R5	dynein axonemal heavy chain 8	DNAH8	515	0.337	0.03202207
Q3MHW9	NADH-cytochrome b5 reductase 1	CYB5R1	34	0.706	0.0380556
P15103	glutamine synthetase	GLUL	42	0.569	0.0384637
F1MFB3	Gem-associated protein 6	GEMIN6	68	0.590	0.03870914
G3N0H2	chromosome 11 C2orf16 homologue	C11H2orf16		0.557	0.03870914
F1MJQ7	fibronectin type III domain-containing 8	FNDC8	35	0.496	0.03870914
Q32KN6	phosphoglycerate kinase	PGK2	45	0.477	0.03875638
A6QPG6	LANCL2 protein	LANCL2	49	0.525	0.03905358
Q17QF5	protein kinase cAMP-dependent type I regulatory subunit beta	PRKAR1B	43	0.734	0.04020682
A0A3Q1M8A8	tudor domain-containing protein 3	TDRD3	83	0.574	0.04123535
F1MNH2	radial spoke head component 1	RSPH1	35	0.279	0.04435444
Q2M2U5	IQ domain-containing protein F2	IQCF2	20	1.126	0.04467807
A6H758	C11H9ORF9 protein	C11H9ORF9	25	0.627	0.04467807
F1MR35	angiotensin-converting enzyme	ACE3	87	0.459	0.04467807
E1BFM2	serine-protease 50	PRSS50	55	0.974	0.04673994
A6QNX2	DPP7 protein	DPP7	54	0.590	0.04710156
F1N0E5	T-complex protein 1 subunit delta	CCT4	58	0.466	0.04710156

a Proteins identified using logistic regression model with Elastic Net penalty, the parameters lambda 0.433 and alpha 0.1; proteins with a VIP > 1.5 in the PLS-DA classification; and proteins identified using the Limma Package with adjusted *p* value ≤ 0.05 were selected.

**Table 3:** List of the Differentially Expressed Proteins in the Capacitated Sperm Group^a^

accession number	protein name	gene name	mass (KDa)	Log FC	adjusted *p* value
P81644	apolipoprotein A-II	APOA2	11	2.186	0.00130631
Q2YDN8	inactive serine/threonine-protein kinase VRK3	VRK3	55	0.352	0.01604564
Q2HJ97	prohibitin-2	PHB2	33	0.417	0.01999291
F1MBU8	Dpy-19 like 2	DPY19L2	90	0.518	0.0384637
F1MMV1	EF-hand domain-containing family member B	EFHB	98	0.426	0.0384637
A0A452DII8	ATP synthase subunit beta	ATP5F1B	62	0.351	0.04049404
Q95KV7	NADH dehydrogenase [ubiquinone] 1 alpha subcomplex subunit 13	NDUFA13	17	0.447	0.04467807
P04038	cytochrome c oxidase subunit 6C	COX6C	9	0.484	0.04700219
P00130	cytochrome *b*-c1 complex subunit 9	UQCR10	7	0.559	0.04710156

a Proteins identified using logistic regression model with Elastic Net penalty, the parameters lambda 0.433 and alpha 0.1; proteins with a VIP > 1.5 in the PLS-DA classification; and proteins identified using the Limma Package with adjusted *p* value ≤ 0.05 were selected.

Among the proteins in the NC spermatozoa group, the 10 most differentially abundant proteins were the metalloprotease inhibitor TIMP2; the serine-protease inhibitor SERPINE 2; two members of the spermadhesin family (SPADH2 and SPADH1); a glycoprotein with a chaperone-like function, clusterin (CLU); the C-type natriuretic peptide; the deoxyribonuclease DNASE1L3; the structural protein cylicin-2 (CYLC2); and the BPI fold-containing family A member 1 (BPIFA1) and the secretoglobin family 1D member (SCGB1D) (Table 4). A total of nine proteins were found to be over-represented in the IVC spermatozoa group (Table 5), of which four are known to be associated with oxidative phosphorylation: the ATP synthase subunit beta (ATP5F1B); the member of complex I of the respiratory chain, NADH dehydrogenase [ubiquinone] 1 alpha subcomplex subunit 13 (NDUFA13); the member of complex IV of the respiratory chain, cytochrome c oxidase subunit 6C (COX6C); and the member of complex III of the respiratory chain, cytochrome *b*-c1 complex subunit 9 (UQCR10). We also found in the IVC spermatozoa group a differential over-representation of the proteins: apolipoprotein A-II (APOA2), serine/threonine-protein kinase VRK3, prohibitin 2 (PHB2), the mannosyltranferase Dpy-19 like 2 (DPY19L2), and EF-hand domain-containing family member B (EFHB).

**Table 4:** List of the Top 10 Differentially Overexpressed Proteins and Their Functional Role in Noncapacitated Bull Sperm According to the UniProt KB Database

gene names	protein names	subcellular location	function
TIMP2	metalloproteinase inhibitor 2	secreted/extracellular space	complexes with metalloproteinases and irreversibly inactivates them by binding to their catalytic zinc cofactor
SERPINE2	Serine-protease inhibitor clade E member 2	secreted/extracellular space	negative regulation of the activities of tPA and other serine proteases
SPADH2	spermadhesin-2	secreted/extracellular space	members of a family of secretory proteins expressed in the male genital tract of the bull. Products of seminal plasma peripherally associated with the sperm surface and involved in different stages of fertilization
SPADH1	spermadhesin-1	secreted/extracellular space	
CLU	clusterin	secreted/extracellular space	ubiquitous glycoprotein with chaperone and antiapoptotic functions
NPPC	C-type natriuretic peptide	secreted/extracellular space	
DNASE1L3	deoxyribonuclease	endoplasmic reticulum/nucleus	DNA hydrolytic activity; apoptotic DNA fragmentation
CYLC2	cylicin-2	cytoskeletal calyx	structural constituent of cytoskeleton; architectural role during spermatogenesis; involved in spermatid differentiation
BPIFA1	BPI fold-containing family A member 1	secreted/extracellular space	immune related protein; bind lipids, display antibacterial activity, and are involved in inflammatory responses
SCGB1D	secretoglobin family 1D member	secreted/extracellular space	member of secretoglobin family; bind androgens and other steroids

**Table 5:** List of the Differentially Overexpressed Proteins and Their Functional Role in IVC Bull Sperm According to the UniProt KB Database

gene names	protein names	subcellular location	function
APOA2	apolipoprotein A-II	secreted/extracellular space	cholesterol transfer activity; antimicrobial activity. Involved in binding with zona pellucida
VRK3	serine/threonine-protein kinase VRK3	nucleus	protein phosphorylation; vesicle-mediated transport signal transduction
PHB2	prohibitin-2	mitochondria (membrane)	inner mitochondrial membrane protein complex; chaperone-like protein; stabilizes mitochondrial respiratory enzymes and maintains mitochondrial integrity
DPY19L2	Dpy-19 like 2	membrane	mannosyltransferase activity spermatid development
EFHB	EF-hand domain-containing family member B (Cilia- and flagella-associated protein 21)	cytoplasm, cytoskeleton, cilium axoneme	microtubule inner protein required for motile cilia beating; cytosolic sensor for calcium
ATP5F1B	ATP synthase subunit beta	mitochondria	mitochondrial proton-transporting ATP synthase complex; Involved in ATP synthesis
NDUFA13	NADH dehydrogenase [ubiquinone] 1 alpha subcomplex subunit 13	mitochondria (membrane)	member of mitochondrial membrane respiratory chain NADH dehydrogenase (complex I); transfers electrons from NADH to the respiratory chain
COX6C	cytochrome c oxidase subunit 6C	mitochondria (membrane)	member of the cytochrome c oxidase (complex IV of the mitochondrial membrane respiratory chain); involved in oxidative phosphorylation
UQCR10	cytochrome *b*-c1 complex subunit 9	mitochondria (membrane)	component of the mitochondrial respiratory chain complex III; involved in mitochondrial electron transport from ubiquinol to cytochrome c

### 3.3 Bioinformatics Analysis

#### 3.3.1 Over-representation Analysis (ORA)

We performed an ORA analysis to compare the differential proteins with a set of reference proteins (whole genome). The enrichment analysis was performed using the g:profiler software (release 22/05/2024) to search for significant differences in the abundance of GO terms associated with proteins in the IVC and NC groups, relative to their abundance in the B. taurus genome. Of the 63 proteins mapped in the NC sperm group (Figure 3), the predominant enriched metabolic function GO terms were citidylate kinase activity [GO:004127] and unfolded protein binding [GO:0051082]; the predominant enriched GO BPs were sexual reproduction [GO:0019953], reproductive process [GO:0022414], fertilization [GO:0009566], spermatid development [GO:0007286], and chaperone-mediated protein folding [GO:0061077]; and the predominant enriched CC GO terms were plasma membrane-bounded cell projection cytoplasm [GO:0032838], secretory granule [GO:0030141], sperm flagellum [GO: 0036126], and acrosomal vesicle [GO0001669] (see all GO terms in supplementary figure S1).

**Figure 3:**
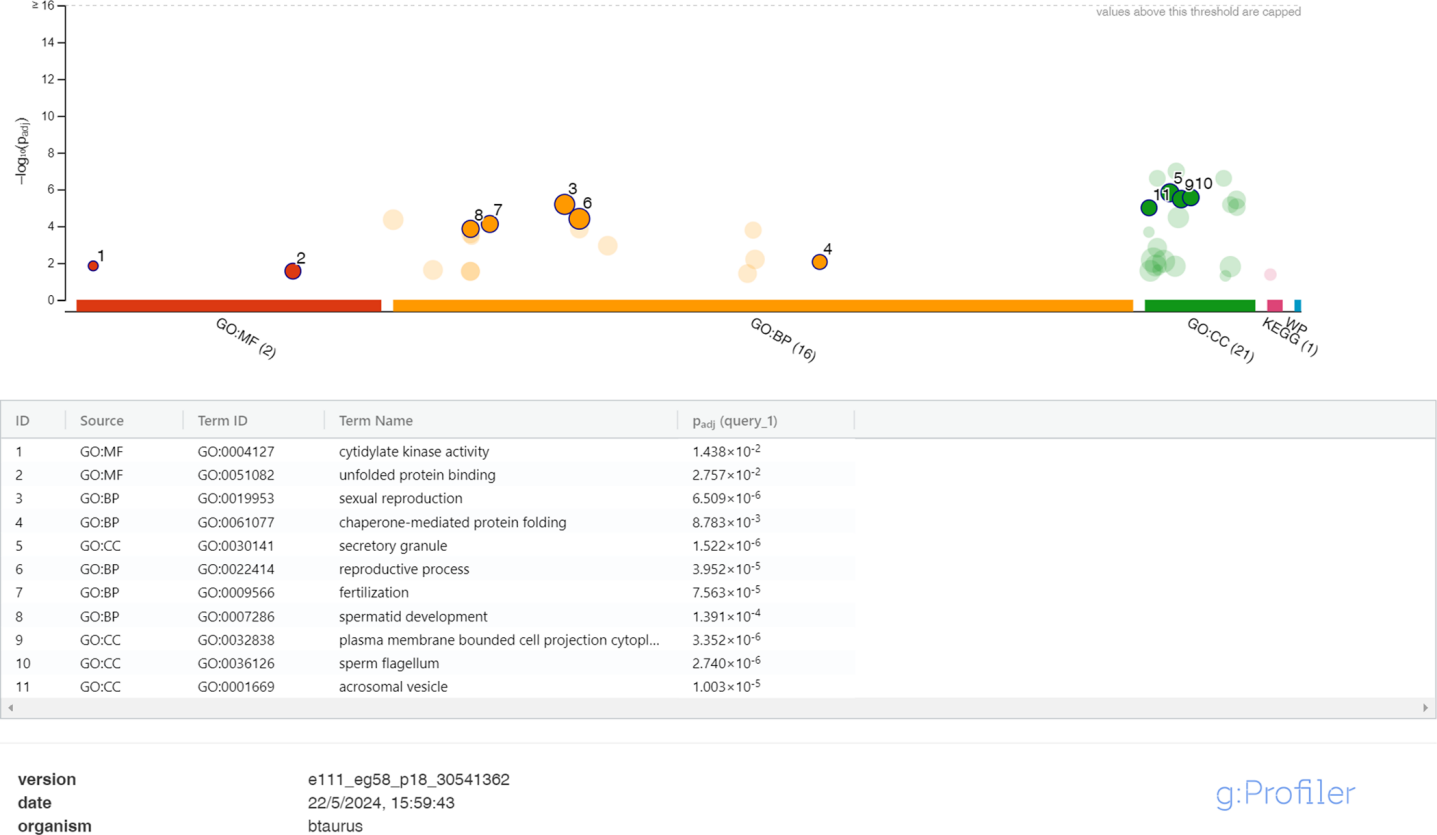
g:GOST multiquery Manhattan plot showing the enrichment analysis of the NC bull sperm (released 22/05/2024). The sperm proteome was queried against the B. taurus proteome database. Selected GO terms corresponding to molecular function (MF) are colored red, BP orange, and CC green.

The nine differentially expressed proteins found in higher abundance in the IVC sperm group (Figure 4) were mapped to CC GO terms. The predominant enriched CC GO terms were related with mitochondrial structure, such as inner mitochondrial membrane protein complex [GO:0098800], mitochondrial protein-containing complex [GO: 0098798], mitochondrial membrane [GO0031966], mitochondrial envelope [GO: 0005740], and mitochondrial prohibitin complex [GO: 0035632]. Protein samples from the IVC sperm group were also enriched in biological pathways related to the mitochondrial function. The predominant enriched biological pathway KEGG or WikiPathway terms were oxidative phosphorylation [KEGG:00190] and electron transport chain [WP: 1002].

**Figure 4:**
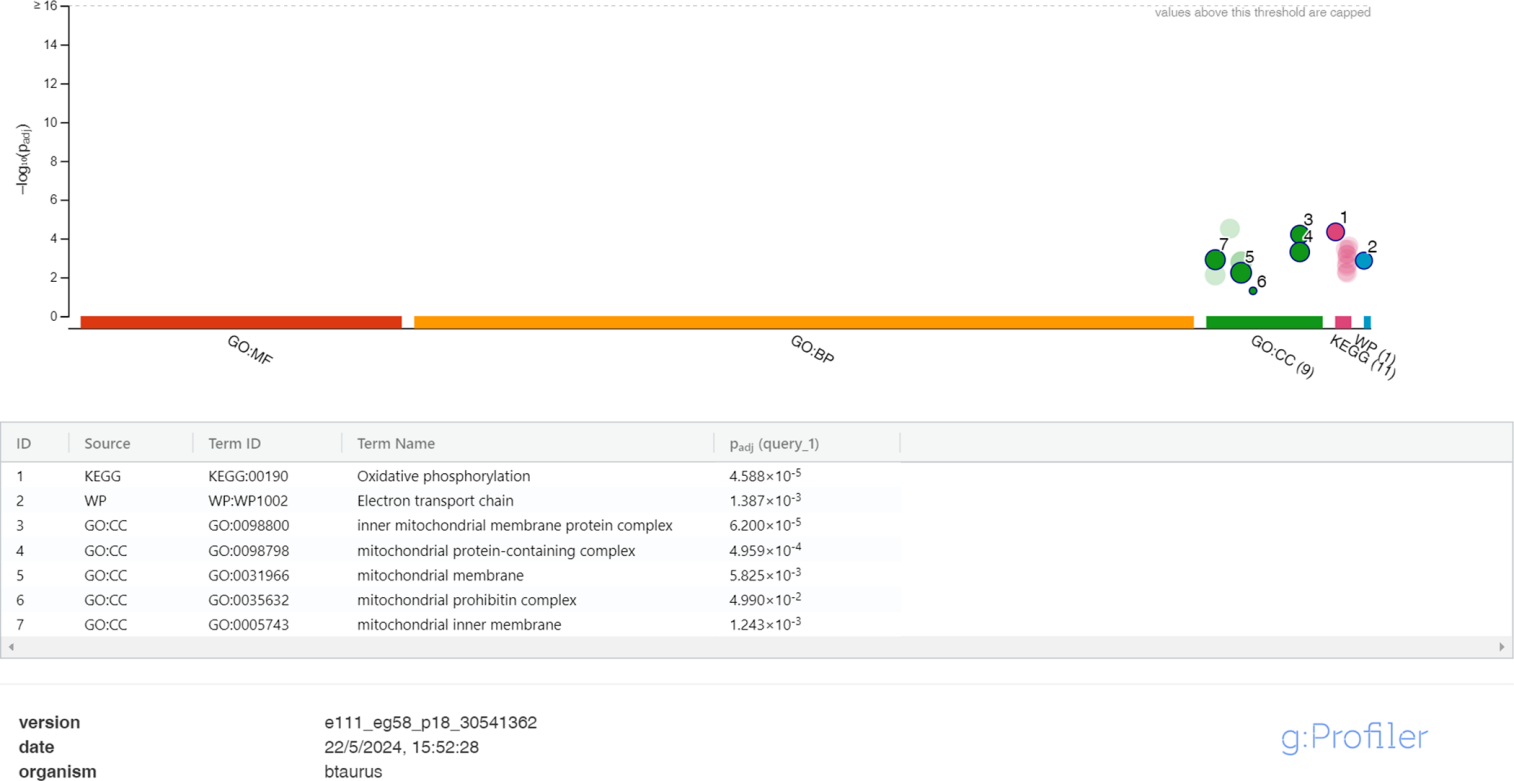
g:GOST multiquery Manhattan plot showing the enrichment analysis of the IVC bull sperm (released 22/05/2024). The sperm proteome was queried against the B. taurus proteome database. GO terms corresponding to CC are in green, KEGG pathways in pink, and the WikiPathway term in blue.

We used the EnrichmentMap Cytoscape app to visualize the results of the protein-set enrichment analysis. The results showed that proteins in the NC sperm group were grouped into two main clusters related to structural features or processes involved in male sexual reproduction (Figure 5A). Proteins in the IVC sperm group were grouped into main clusters related to mitochondrial activity and structure (Figure 5B).

**Figure 5:**
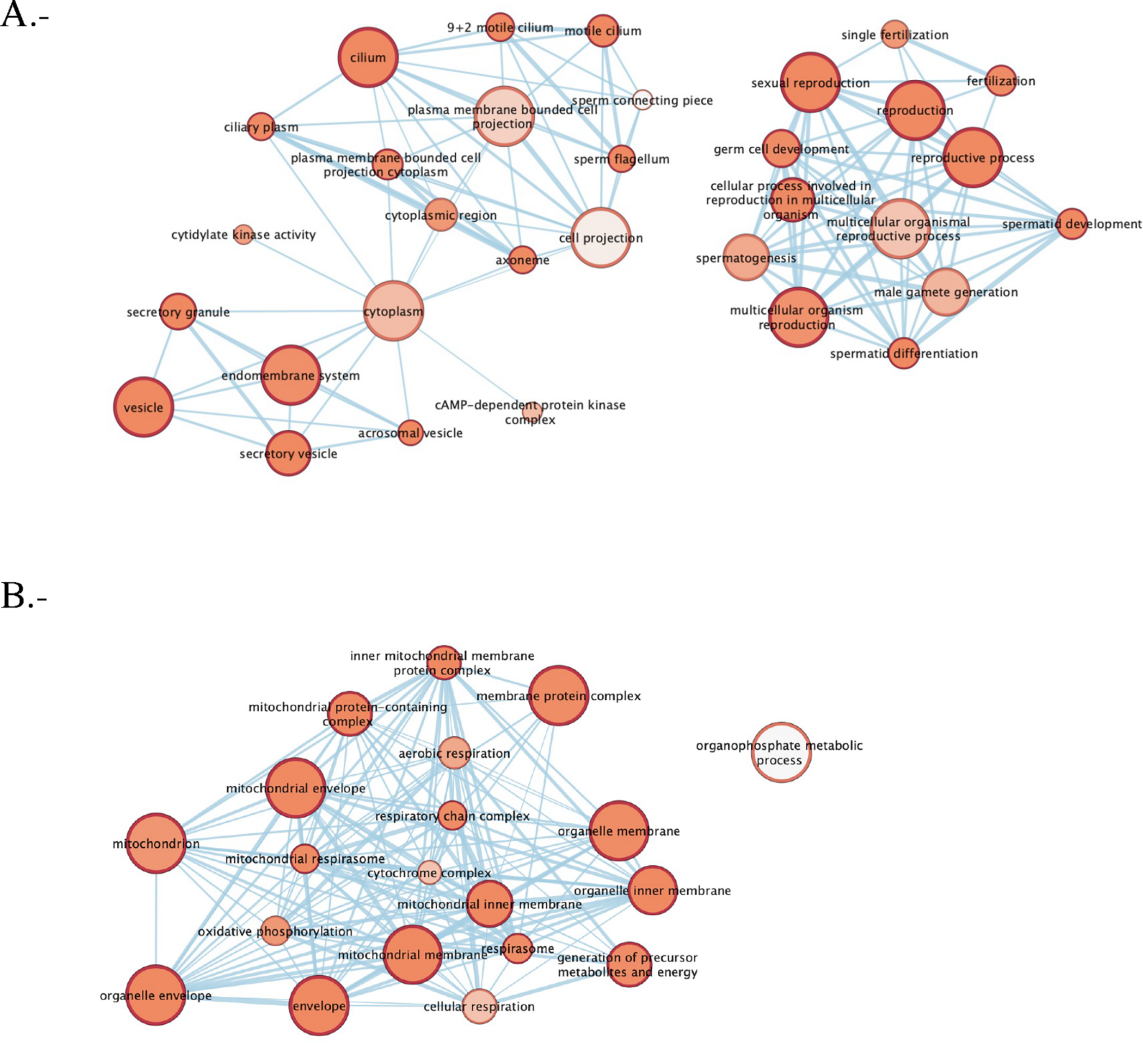
Representation of the protein-set enrichment networks of the (A) NC and (B) IVC sperm groups. Edges represent mutual overlap, and nodes represent protein sets. Highly redundant protein sets are clustered together. The figure was generated by using the EnrichmentMap APP for Cytoscape.

#### 3.3.2 Protein–Protein Interaction

A functional protein association network between the 72 DAPs was assessed using the STRING database. A total of 69 nodes and 70 edges were identified in the B. taurus spermatozoa (Figure 6). To identify the DAPs of each group, the top 10 DAPs of the NC and the DAPs of the IVC sperm groups were represented in green and red, respectively. This analysis highlighted that proteins were clustered into three protein networks that were functionally enriched in three BPs: fertilization, sperm capacitation, and oxidative phosphorylation (Table 6). DAPs from the NC sperm group were mostly clustered in the fertilization protein cluster, whereas those from the IVC sperm group were clustered in the oxidative phosphorylation cluster.

**Figure 6:**
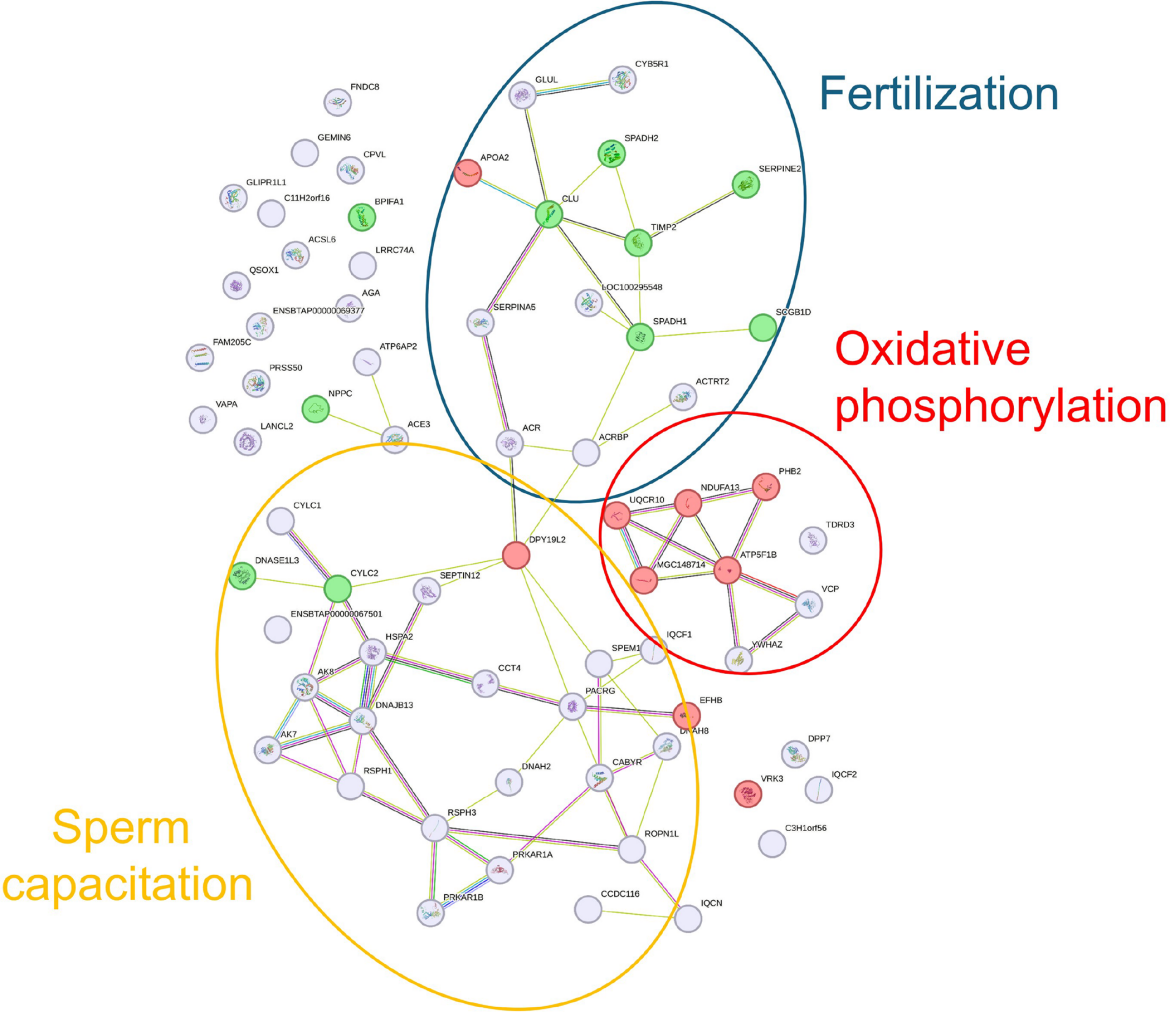
STRING protein–protein interaction network showing the interactions of the differentially abundant proteins on IVC and NC bull sperm. Proteins were clustered in three functional networks enriched in the following BPs: fertilization (blue circle), sperm capacitation (yellow circle), and oxidative phosphorylation (red circle). The top 10 DAPs of the NC sperm group have been colored in green, and the proteins from the IVC sperm group are in red.

**Table 6:** GO Terms Correspond to BPs Enriched in the Functional Networks^a^

biological process	GO term	enrichment fold	FDR
fertilization	GO:0007338 single fertilization	1.7	0.0073
	GO:0009566 fertilization	1.7	0.00054
sperm capacitation	GO:20000480 negative regulation of cAMP-dependent protein kinase activity	2.26	0.0486
	GO:0048240 sperm capacitation	2.01	0.0035
	GO:0007286 spermatid development	1.68	1.12 × 10^–8^
	GO:0061077 chaperone-mediated protein folding	1.62	0.0392
	GO:0007283 spermatogenesis	1.64	8.02 × 10^–10^
oxidative phosphorylation	GO:0006119 oxidative phosphorylation	1.9	0.0385
	GO: 0022900 electron transport chain	1.83	0.0395
	GO:0006091 generation of precursor metabolites and energy	1.58	0.0232
	GO:0006886 intracellular protein transport	1.32	0.0486

a The proteins were clustered using string software.

#### 3.3.3 GSEA

This analysis provides us with a ranking of protein sets, such as biological pathways or ontology terms, according to their correlation with our variable of interest (capacitation). Results show a higher representation of the protein sets related to mitochondria, mitochondria inner membrane, or mitochondria-protein-containing complex in the IVC sperm group. They also show a higher representation of the set of proteins related with cytoplasmic vesicles, secretory vesicles, extracellular region, or sexual reproduction in the NC sperm group (Figure 7).

**Figure 7:**
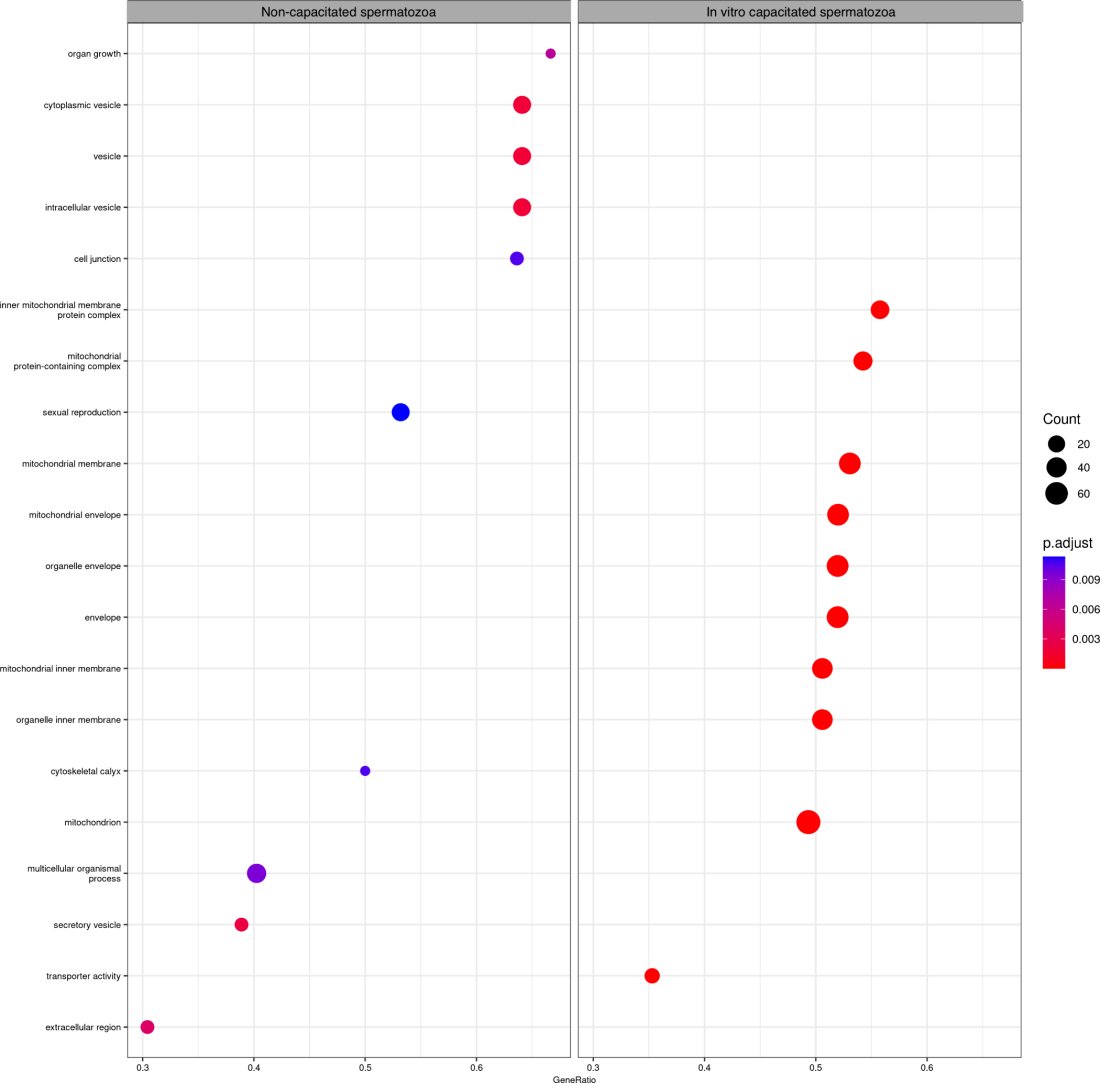
Dot plot of protein-set enrichment analysis of the NC and IVC sperm groups. The number of proteins overlapping is indicated by the diameters of the dots. The color indicates the adjusted *p* value. The figure was generated using the R package clusterProfiler.

## 4 Discussion

Sperm capacitation is the final step in post-testicular maturation, and it is necessary for spermatozoa to attain their full fertilizing capacity. However, the process remains incompletely understood, with limited information available regarding the proteins involved. Therefore, the aim of this study was to investigate the effect of IVC on the proteome and mitochondrial parameters of bull spermatozoa.

Our results showed changes in the proteomic profile of the IVC spermatozoa, with some of the 72 DAPs being over- and others under-represented. These changes in the differential protein amount after IVC have also been observed in other species (human,^11–13^ mouse,^14,15^ porcine,^16–18^ ovine,^19^ and buffalo^20^). We point out that the studies referenced above are not homogeneous in terms of the type of methodology used for protein extraction or the proteomic techniques employed for their analysis. Therefore, we should be cautious with the comparisons with other studies, since the type of protein extraction procedure may influence the efficiency of sperm protein recovery. The data indicated a loss or/and under-representation of DAPs during sperm capacitation rather than a gain.^12,13^ Indeed, the top 10 most significant DAPs were found in the NC spermatozoa. Although we did not assess the acrosome status in this work, we assumed that acrosome loss occurred during the capacitation process. Following IVC treatment, a significant increase in live capacitated sperm assessed by M540 was observed, validating the efficiency of the IVC treatment. Rathi et al.^32^ demonstrated that M540-positive sperm corresponded to capacitated or acrosome-reacted sperm evaluated by chlortetracycline staining.^3^ Consequently, it is likely that many proteins associated with the sperm membrane and acrosome were lost. During ejaculation, spermatozoa mix with seminal plasma compounds that include a set of secretory proteins, some of which were among the DAPs identified in this study, such as spermadhesins (SPADH2, SPADH1), CLU, NPPC, SERPINE2, TIMP2, DNASE1L3, and SCGB1D.^33–38^ Spermadhesins and other proteins associated with the sperm membrane are often lost after IVC, except for phospholipid-bound spermadhesins.^34,35^ Among these DAPs, SERPINE2, a seminal vesicle protein acting as a decapacitation factor, was notable as its mere presence inhibits and reverses the capacitation effect of BSA, thus inhibiting both the rate of capacitated and acrosome-reacted sperm.^34,39^ In addition to those secreted into the seminal plasma, cylicin-1 (CYLC1) was among the top 10 DAPs in the NC sperm. CYLC1, a perinuclear theca protein mainly localized in the calix,^40^ has been recently linked to acrosome formation and male infertility.^41,42^ Recently, Chhikara et al.^13^ also observed that CYLC1 was over-represented in NC sperms compared to acrosome-reacted sperms after IVC treatment. However, other proteomic studies have found the opposite in capacitated sperm.^20^ TIMP2, CLU, NPPC, and SPADH1 are also plasma seminal proteins whose higher abundance is associated with higher ejaculate quality or fertility bulls.^36,43^ The plasma protein DNASE1L3 hydrolyzes DNA during apoptosis, and a greater expression of DNASE1L3 was observed in bull spermatozoa with high freezability.^33^ When GOs of over-represented DAPs in NC sperm were analyzed, these proteins were mainly related to structures such as the acrosome (secretory granules/acrosomal vesicle) or the flagellum (motile cilium). Furthermore, GOs related to sexual reproduction or male gamete generation were highlighted in NC sperm but did not appear in the IVC ones. The acrosome is a unique vesicle of the spermatozoon, and its presence alone explains why these GOs are identified in the NC group. Capacitated sperm should have suffered a greater spontaneous acrosome loss during the capacitation process, despite the absence of an acrosome reaction inducer, as observed in other studies.^44^ Similar results with phosphorylated DAPS were observed in yak spermatozoa.^45^

After a 4 h IVC treatment, spermatozoa showed a reduction of high MMP and an increase of superoxide anion or mROS levels. However, these changes were masked by a reduction in sperm viability during capacitation, as observed in other studies.^22,46,47^ Levels of mROS could be one of the factors implicated in this loss of sperm viability and motility since apoptosis induced via phosphoinositide 3-kinase suppression implied mROS generation and a reduction of viability and motility in human sperm.^10,48^ Thus, we studied the effect of IVC on live sperm populations and found that live IVC sperm were less ″MH″ than NC sperm, primarily due to an increase in mROS rather than a decrease in high MMP. Bovine sperm are highly dependent on ATP availability for capacitation.^49,50^ To obtain ATP, spermatozoa can use different energy pathways and substrates. The most common metabolic pathways include glycolysis or the mitochondrial Krebs cycle and OXPHOS. The OXPHOS pathway is the most efficient way of obtaining ATP per molecule of glucose;^49,50^ however, it requires oxygen and is localized to the midpiece of sperm where mitochondria are enclosed. The predominant or exclusive metabolic pathway remains an open debate, as it depends on the species, the energy substrate in the environment, oxygen availability, and the BP involved.^50^

In bovine sperm, literature indicates that both pathways are involved in motility^51^ and capacitation.^49^ During capacitation, a considerable energy supply is needed,^2^ and mitochondrial activity appears to increase in both mouse and human spermatozoa.^8,9^

In line with our results, other studies also found increased mROS after IVC treatment.^8,9^ Superoxide is produced in the mitochondrial electron transport chain (ETC), mainly in complex I (NADH: ubiquinone oxidoreductase) and III (NADH: ubiquinone oxidoreductase).^52^ One factor affecting the rate of superoxide anion production is the ETC protein concentration in the mitochondria.^53^ Differences in complex I protein concentration explained the differences in H_2_O_2_ production between rat and pigeon heart mitochondria.^52^ Our study showed that the IVC sperm group had a significant over-representation of ECT proteins, including all complexes and ATP synthase complex in the OXPHOS pathway. Moreover, functional enrichment analysis showed that ETC, OXPHOS pathways, and intracellular protein transport were BP over-represented in IVC spermatozoa. Several proteomic studies have found similar results in different livestock species. In porcine, the abundance of ubiquinol-cytochrome c reductase complex increased in IVC spermatozoa.^17,54^ Similarly, Peris-Frau et al.^19^ observed that DLAT, a mitochondrion matrix protein of pyruvate dehydrogenase complex, was over-represented in capacitated ovine sperm. Consistent with our results, ATP synthase complex subunit proteins and energy production were also over-represented in a human-capacitated and acrosome-reacted sperm.^12^ Moreover, we found an over-representation of PHB2, a mitochondrial membrane protein regulating mitochondrial respiration mainly in stress situation,^55^ in the IVC spermatozoa as it was previously observed in human-capacitated and acrosome-reacted sperm.^12^ In this work, we observed that APOA2 was overexpressed in IVC bull sperm. This protein is one of the most abundant in the high-density lipoprotein (HDL) complex and is related with APOA1, which was overexpressed in boar sperm after capacitation.^16^

Although mature spermatozoa are transcriptionally inactive, several studies have observed a significant increase in DAPs after capacitation.^12,15–20^ As discussed by Skerret-Byrne et al.,^15^ one explanation could be that post-translational changes, unmasking and/or repositioning of key elements of the intrinsic sperm proteome may also occur during capacitation, which could be affected by the different extraction methods and consequently could affect the differential abundance obtained. Another explanation,^15^ even if it goes against the central dogma, would be that there is a certain capacity for RNA translation, as other authors have observed.^14,56,57^ These studies showed that the use of mitochondrial translation inhibitors, such as chloramphenicol, during sperm capacitation reduces nuclear-encoded protein content.^14,56,57^ Moreover, chloramphenicol partially inhibited the incorporation of labeled lysine into ATP1A4 synthesis during bull sperm capacitation.^57^ However, the precise mechanisms involved in these processes remain unclear.

Finally, it is important to note that the sperm samples used in this study were frozen doses. During cryopreservation, frozen/thawed sperm are subjected to changes in the amount and distribution of their proteins, including post-translational modifications.^58^ Many studies have found DAPs both over- and under-represented in frozen/thawed spermatozoa.^58,59^ Differences between different freezing protocols have even been observed recently.^60,61^ Many of these observed changes are similar to those undergone during capacitation, such as phosphorylation of tyrosine residues; in fact, the term cryocapacitation is sometimes used.^58^

## 5 Conclusion

Based on all the above, we could conclude that during the capacitation process, there is a significant loss of seminal plasma-derived membrane proteins and a significant increase in proteins related to the OXPHOS pathway. Additionally, a significant increase in superoxide production and in mitochondrial activity was observed among the IVC bull spermatozoa.

## Supplementary Material



## Notes

g:GOST multiquery Manhattan plot showing the complete list of the GO terms released after the enrichment analysis of NC bull sperm (Figure S1); full list of the 802 identified proteins after the proteomic analysis by SWATH LC/MS-MS, of NC and IVC sperm samples (Table S1) (PDF)

## Abbreviations

PKA: Protein kinase A
cAMP: cyclic adenosine monophosphate
CASA: Computer-assisted sperm analysis
pHi: Intracellular pH
FC: Flow cytometry
SWATH-MS: Sequential Window Acquisition of all Theoretical Mass Spectra
MMP: Mitochondrial membrane potential
mROS: Mitochondrial reactive oxygen species
DAPs: Differentially abundant proteins
TL: Tyrode’s medium
NC: Noncapacitated treatment
IVC: *In vitro* capacitation treatment
DAPI: 4’,6’-Diamidino-2-phenylindole
MTDR: Mitotracker Deep Red
MSOX: MitosoxTM Red
M540: Merocyanine 540
TFA: Trifluoroacetic acid
ACN: Acetonitrile
FA: Formic acid
LC-MS/MS: Liquid chromatography and tandem mass spectrometry
BP: Biological process
CC: Cellular component
MP: Molecular process
FDR: False discovery rate
OXPHOS: Oxidative phosphorylation pathway

## References

[1] Baker M. A., Nixon B., Naumovski N., Aitken R. J. (2012). Proteomic Insights
into the Maturation and Capacitation of Mammalian Spermatozoa. Syst. Biol. Reprod Med..

[2] Stival C., Puga Molina L. D. C., Paudel B., Buffone M. G., Visconti P. E., Krapf D. (2016). Sperm Capacitation
and Acrosome Reaction in Mammalian Sperm. Adv.
Anat Embryol Cell Biol..

[3] Gadella B. M., Rathi R., Brouwers J. F. H. M., Stout T. A. E., Colenbrander B. (2001). Capacitation
and the Acrosome Reaction in Equine Sperm. Anim
Reprod Sci..

[4] Darszon A., Nishigaki T., López-González I., Visconti P. E., Treviño C. L. (2020). Differences and Similarities: The
Richness of Comparative Sperm Physiology. Physiology.

[5] Puga
Molina L. C., Luque G. M., Balestrini P. A., Marín-Briggiler C. I., Romarowski A., Buffone M. G. (2018). Molecular Basis of Human Sperm Capacitation. Front. Cell Dev. Biol..

[6] Buffone M. G., Hirohashi N., Gerton G. L. (2014). Unresolved Questions Concerning Mammalian
Sperm Acrosomal Exocytosis1. Biol. Reprod..

[7] De
Lamirande E., O’flaherty C. (2008). Sperm Activation: Role of Reactive
Oxygen Species and Kinases. Biochim. Biophys.
Acta, Proteins Proteomics.

[8] Irigoyen P., Mansilla S., Castro L., Cassina A., Sapiro R. (2024). Mitochondrial
Function and Reactive Oxygen Species Production during Human Sperm
Capacitation: Unraveling Key Players. FASEB
J..

[9] Ferreira J. J., Cassina A., Irigoyen P., Ford M., Pietroroia S., Peramsetty N., Radi R., Santi C. M., Sapiro R. (2021). Increased
Mitochondrial Activity upon CatSper Channel Activation Is Required
for Mouse Sperm Capacitation. Redox Biol..

[10] Aitken R., Baker M., Nixon B. (2015). Are Sperm
Capacitation and Apoptosis
the Opposite Ends of a Continuum Driven by Oxidative Stress?. Asian J. Androl.

[11] Secciani F., Bianchi L., Ermini L., Cianti R., Armini A., La Sala G. B., Focarelli R., Bini L., Rosati F. (2009). Protein Profile
of Capacitated versus Ejaculated Human Sperm. J. Proteome Res..

[12] Castillo J., Bogle O. A., Jodar M., Torabi F., Delgado-Dueñas D., Estanyol J. M., Ballescà J. L., Miller D., Oliva R. (2019). Proteomic
Changes in Human Sperm During Sequential in Vitro Capacitation and
Acrosome Reaction. Front Cell Dev Biol..

[13] Chhikara N., Tomar A. K., Datta S. K., Yadav S. (2023). Proteomic Changes in
Human Spermatozoa during in Vitro Capacitation and Acrosome Reaction
in Normozoospermia and Asthenozoospermia. Andrology.

[14] Zhao C., Guo X., Shi Z., Wang F., Huang X., Huo R., Zhu H., Wang X., Liu J., Zhou Z., Sha J. (2009). Role of Translation
by Mitochondrial-type Ribosomes during Sperm Capacitation: An Analysis
Based on a Proteomic Approach. Proteomics.

[15] Skerrett-Byrne D. A., Anderson A. L., Bromfield E. G., Bernstein I. R., Mulhall J. E., Schjenken J. E., Dun M. D., Humphrey S. J., Nixon B. (2022). Global Profiling of the Proteomic Changes Associated with the Post-Testicular
Maturation of Mouse Spermatozoa. Cell Rep.

[16] Kwon W.-S., Rahman M. S., Lee J.-S., Kim J., Yoon S.-J., Park Y.-J., You Y.-A., Hwang S., Pang M.-G. (2014). A Comprehensive
Proteomic Approach to Identifying Capacitation Related Proteins in
Boar Spermatozoa. BMC Genomics.

[17] Choi Y. J., Uhm S. J., Song S. J., Song H., Park J. K., Kim T., Park C., Kim J. H. (2008). Cytochrome c Upregulation during
Capacitation and Spontaneous Acrosome Reaction Determines the Fate
of Pig Sperm Cells: Linking Proteome Analysis. Journal of Reproduction and Development.

[18] Zigo M., Kerns K., Sutovsky P. (2023). The Ubiquitin-Proteasome
System Participates
in Sperm Surface Subproteome Remodeling during Boar Sperm Capacitation. Biomolecules.

[19] Peris-Frau P., Martín-Maestro A., Iniesta-Cuerda M., Sánchez-Ajofrín I., Mateos-Hernández L., Garde J. J., Villar M., Soler A. J. (2019). Freezing–Thawing
Procedures Remodel the Proteome of Ram Sperm before and after In Vitro
Capacitation. Int. J. Mol. Sci..

[20] Hou Z., Fu Q., Huang Y., Zhang P., Chen F., Li M., Xu Z., Yao S., Chen D., Zhang M. (2019). Comparative Proteomic
Identification Buffalo Spermatozoa during in Vitro Capacitation. Theriogenology.

[21] Gacem S., Castello-Ruiz M., Hidalgo C. O., Tamargo C., Santolaria P., Soler C., Yániz J. L., Silvestre M. A. (2023). Bull Sperm
SWATH-MS-Based Proteomics Reveals Link between High Fertility and
Energy Production, Motility Structures, and Sperm–Oocyte Interaction. J. Proteome Res..

[22] Yániz J. L., Palacín I., Silvestre M. A., Hidalgo C. O., Tamargo C., Santolaria P. (2021). Ability of
the ISAS3Fun Method to Detect Sperm Acrosome
Integrity and Its Potential to Discriminate between High and Low Field
Fertility Bulls. Biology (Basel).

[23] Pérez-Patiño C., Parrilla I., Li J., Barranco I., Martínez E. A., Rodriguez-Martínez H., Roca J. (2019). The Proteome of Pig
Spermatozoa Is Remodeled During Ejaculation. Molecular & Cellular Proteomics.

[24] Roy D., Dey S., Majumder G. C., Bhattacharyya D. (2015). Role of Epididymal Anti Sticking
Factor in Sperm Capacitation. Biochem. Biophys.
Res. Commun..

[25] Giaccagli M. M., Gómez-Elías M. D., Herzfeld J. D., Marín-Briggiler C. I., Cuasnicú P. S., Cohen D. J., Da Ros V. G. (2021). Capacitation-Induced
Mitochondrial Activity Is Required for Sperm Fertilizing Ability in
Mice by Modulating Hyperactivation. Front. Cell
Dev. Biol..

[26] Hallap T., Nagy S., Jaakma Ü., Johannisson A., Rodriguez-Martinez H. (2005). Mitochondrial Activity of Frozen-Thawed
Spermatozoa
Assessed by MitoTracker Deep Red 633. Theriogenology.

[27] Peña F. J., Johannisson A., Wallgren M., Rodriguez-Martinez H. (2004). Effect of
Hyaluronan Supplementation on Boar Sperm Motility and Membrane Lipid
Architecture Status after Cryopreservation. Theriogenology.

[28] Kotwicka M., Skibinska I., Jendraszak M., Jedrzejczak P. (2016). 17β-Estradiol
Modifies Human Spermatozoa Mitochondrial Function in Vitro. Reproductive Biology and Endocrinology.

[29] Shevchenko A., Wilm M., Vorm O., Mann M. (1996). Mass Spectrometric
Sequencing of Proteins from Silver-Stained Polyacrylamide Gels. Anal. Chem..

[30] Pérez-Patiño C., Parrilla I., Barranco I., Vergara-Barberán M., Simó-Alfonso E. F., Herrero-Martínez J. M., Rodriguez-Martínez H., Martínez E. A., Roca J. (2018). New In-Depth Analytical Approach of the Porcine Seminal Plasma Proteome
Reveals Potential Fertility Biomarkers. J. Proteome
Res..

[31] Shilov I. V., Seymour S. L., Patel A. A., Loboda A., Tang W. H., Keating S. P., Hunter C. L., Nuwaysir L. M., Schaeffer D. A. (2007). The Paragon
Algorithm, a Next Generation Search Engine That Uses Sequence Temperature
Values and Feature Probabilities to Identify Peptides from Tandem
Mass Spectra. Molecular & Cellular Proteomics.

[32] Rathi R., Colenbrander B., Bevers M. M., Gadella B. M. (2001). Evaluation of In
Vitro Capacitation of Stallion Spermatozoa. Biol. Reprod..

[33] Rego J. P. A., Martins J. M., Wolf C. A., Van Tilburg M., Moreno F., Monteiro-Moreira A. C., Moreira R. A., Santos D. O., Moura A. A. (2016). Proteomic Analysis of Seminal Plasma and Sperm Cells
and Their Associations with Semen Freezability in Guzerat Bulls. J. Anim Sci..

[34] Lu C. H., Lee R. K. K., Hwu Y. M., Chu S. L., Chen Y. J., Chang W. C., Lin S. P., Li S. H. (2011). SERPINE2, a Serine
Protease Inhibitor Extensively Expressed in Adult Male Mouse Reproductive
Tissues, May Serve as a Murine Sperm Decapacitation Factor. Biol. Reprod..

[35] Töpfer-Petersen E., Romero A., Varela P. F., Ekhlasi-Hundrieser M., Dostàlovà Z., Sanz L., Calvete J. J. (1998). Spermadhesins:
A New Protein Family. Facts, Hypotheses and Perspectives. Andrologia.

[36] Viana A. G. A., Martins A. M. A., Pontes A. H., Fontes W., Castro M. S., Ricart C. A. O., Sousa M. V., Kaya A., Topper E., Memili E., Moura A. A. (2018). Proteomic
Landscape
of Seminal Plasma Associated with Dairy Bull Fertility. Sci. Rep.

[37] Boe-Hansen G. B., Rego J. P. A., Crisp J. M., Moura A. A., Nouwens A. S., Li Y., Venus B., Burns B. M., McGowan M. R. (2015). Seminal Plasma Proteins
and Their Relationship with Percentage of Morphologically Normal Sperm
in 2-Year-Old Brahman (Bos Indicus) Bulls. Anim
Reprod Sci..

[38] Gomes F. P., Park R., Viana A. G., Fernandez-Costa C., Topper E., Kaya A., Memili E., Yates J. R., Moura A. A. (2020). Protein Signatures of Seminal Plasma from Bulls with
Contrasting Frozen-Thawed Sperm Viability. Sci.
Rep.

[39] Li S. H., Hwu Y. M., Lu C. H., Lin M. H., Yeh L. Y., Lee R. K. K. (2018). Serine Protease
Inhibitor SERPINE2 Reversibly Modulates
Murine Sperm Capacitation. Int. J. Mol. Sci..

[40] Longo F. J., Krohne G., Franke W. W. (1987). Basic Proteins
of the Perinuclear
Theca of Mammalian Spermatozoa and Spermatids: A Novel Class of Cytoskeletal
Elements. J. Cell Biol..

[41] Schneider S., Kovacevic A., Mayer M., Dicke A. K., Arévalo L., Koser S. A., Hansen J. N., Young S., Brenker C., Kliesch S., Wachten D., Kirfel G., Struenker T., Tüttelmann F., Schorle H. (2023). Cylicins Are a Structural Component
of the Sperm Calyx Being Indispensable for Male Fertility in Mice
and Human. Elife.

[42] Jin H. J., Fan Y., Yang X., Dong Y., Zhang X. Z., Geng X. Y., Yan Z., Wu L., Ma M., Li B., Lyu Q., Pan Y., Liu M., Kuang Y., Chen S. R. (2024). Disruption in CYLC1
Leads to Acrosome Detachment, Sperm Head Deformity, and Male in/Subfertility
in Humans and Mice. Elife.

[43] Mostek A., Westfalewicz B., Słowińska M., Dietrich M. A., Judycka S., Ciereszko A. (2018). Differences in Sperm Protein Abundance
and Carbonylation Level in Bull Ejaculates of Low and High Quality. PLoS One.

[44] Mostek A., Janta A., Majewska A., Ciereszko A. (2021). Bull Sperm
Capacitation Is Accompanied by Redox Modifications of Proteins. Int. J. Mol. Sci..

[45] Zhang R., Liang C., Guo X., Bao P., Pei J., Wu F., Yin M., Chu M., Yan P. (2022). Quantitative Phosphoproteomics
Analyses Reveal the Regulatory Mechanisms Related to Frozen-Thawed
Sperm Capacitation and Acrosome Reaction in Yak (Bos Grunniens). Front. Physiol.

[46] Dudkiewicz S., Peris-Frau P., Nieto-Cristóbal H., Santiago-Moreno J., de Mercado E., Álvarez-Rodríguez M. (2024). Bicarbonate
and BSA Increase the Capacitation Pattern and Acrosomal Exocytosis
in Boar Sperm after 120 min of Incubation. Reproduction
in Domestic Animals.

[47] Ruiz-Díaz S., Grande-Pérez S., Arce-López S., Tamargo C., Olegario
Hidalgo C., Pérez-Cerezales S. (2020). Changes in the Cellular
Distribution of Tyrosine Phosphorylation and Its Relationship with
the Acrosomal Exocytosis and Plasma Membrane Integrity during In Vitro
Capacitation of Frozen/Thawed Bull Spermatozoa. Int. J. Mol. Sci..

[48] Koppers A. J., Mitchell L. A., Wang P., Lin M., Aitken R. J. (2011). Phosphoinositide
3-Kinase Signalling Pathway Involvement in a Truncated Apoptotic Cascade
Associated with Motility Loss and Oxidative DNA Damage in Human Spermatozoa. Biochem. J..

[49] Dahan T., Breitbart H. (2022). Involvement of Metabolic Pathway in the Sperm Spontaneous
Acrosome Reaction. Theriogenology.

[50] Amaral A. (2022). Energy Metabolism
in Mammalian Sperm Motility. WIREs Mechanisms
of Disease.

[51] Gallo A., Esposito M. C., Tosti E., Boni R. (2021). Sperm Motility, Oxidative
Status, and Mitochondrial Activity: Exploring Correlation in Different
Species. Antioxidants.

[52] St-Pierre J., Buckingham J. A., Roebuck S. J., Brand M. D. (2002). Topology of Superoxide
Production from Different Sites in the Mitochondrial Electron Transport
Chain. J. Biol. Chem..

[53] Murphy M. P. (2009). How Mitochondria
Produce Reactive Oxygen Species. Biochem. J..

[54] Kwon W. S., Rahman M. S., Ryu D. Y., Park Y. J., Pang M. G. (2015). Increased
Male Fertility Using Fertility-Related Biomarkers. Sci. Rep..

[55] Coates P. J., Nenutil R., McGregor A., Picksley S. M., Crouch D. H., Hall P. A., Wright E. G. (2001). Mammalian Prohibitin Proteins Respond
to Mitochondrial Stress and Decrease during Cellular Senescence. Exp. Cell Res..

[56] Gur Y., Breitbart H. (2006). Mammalian
Sperm Translate Nuclear-Encoded Proteins
by Mitochondrial-Type Ribosomes. Genes Dev..

[57] Rajamanickam G. D., Kastelic J. P., Thundathil J. C. (2017). Content of Testis-Specific Isoform
of Na/K-ATPase (ATP1A4) Is Increased during Bovine Sperm Capacitation
through Translation in Mitochondrial Ribosomes. Cell Tissue Res..

[58] Pini T., Leahy T., de Graaf S. P. (2018). Sublethal Sperm
Freezing Damage:
Manifestations and Solutions. Theriogenology.

[59] Larbi A., Li C., Quan G. (2024). An Updated
Review on the Application of Proteomics
to Explore Sperm Cryoinjury Mechanisms in Livestock Animals. Anim Reprod Sci..

[60] Parrilla I., Cambra J. M., Cuello C., Rodriguez-Martinez H., Gil M. A., Martinez E. A. (2024). Cryopreservation
of Highly Extended
Pig Spermatozoa Remodels Its Proteome and Counteracts Polyspermic
Fertilization in Vitro. Andrology.

[61] Corda P. O., Silva J. V., Pereira S. C., Barros A., Alves M. G., Fardilha M. (2022). Bioinformatic Approach
to Unveil Key Differentially
Expressed Proteins in Human Sperm After Slow and Rapid Cryopreservation. Front. Cell Dev. Biol..

