# Supplementary material for: Effect of Capacitation on Proteomic Profile and Mitochondrial Parameters of Spermatozoa in Bulls
Source: J Proteome Res. 2025 Mar 25;24(4):1817–31. doi: 10.1021/acs.jproteome.4c00910 (PMC12128097; doi:10.1021/acs.jproteome.4c00910)
Supplement: Supplementary file 1 [file pr4c00910_si_001.pdf]

Effect of capacitation on proteomic profile and mitochondrial parameters of spermatozoa in bulls.

*María Castelló-Ruiz[1,2], Sabrina Gacem[1], Manuel M. Sánchez Del Pino[3], Carlos O. Hidalgo[4], Carolina Tamargo[4], Manuel Álvarez-Rodríguez[5], Jesús L. Yániz[6], Miguel A. Silvestre[1]**

[1]Department of Cell Biology, Functional Biology and Physical Anthropology Universitat de València, 46100 Valencia, Spain. E-mail: miguel.silvestre@uv.es

[2]Unidad Mixta de Investigación Cerebrovascular, Instituto de Investigación Sanitaria La Fe, Hospital Universitario y Politécnico La Fe, Valencia, Spain.

[3]Department of Biochemistry and Molecular Biology, Institute for Biotechnology and Biomedicine (BIOTECMED), University of Valencia, Valencia, Spain.

[4]Animal Selection and Reproduction Area, Regional Agrifood Research and Development Service (SERIDA), 33394 Deva, Gijón, Spain

[5]Department of Animal Reproduction, Spanish National Institute for Agricultural and Food Research and Technology (INIA-CSIC), 28040 Madrid, Spain.

List of contents:

Figure S1: g:GOST multiquery Manhattan plot showing the complete list of the GO terms released after the enrichment analysis of NC bull sperm.

Table S1: Full list of the 802 identified proteins after the proteomic analysis by SWATH LC/MS-MS, of non-capacitated (NC) and *in-vitro* capacitated (IVC) sperm samples.

Figure S1: g:GOST multiquery Manhattan plot showing the complete list of the GO terms released after the enrichment analysis of NC bull sperm.

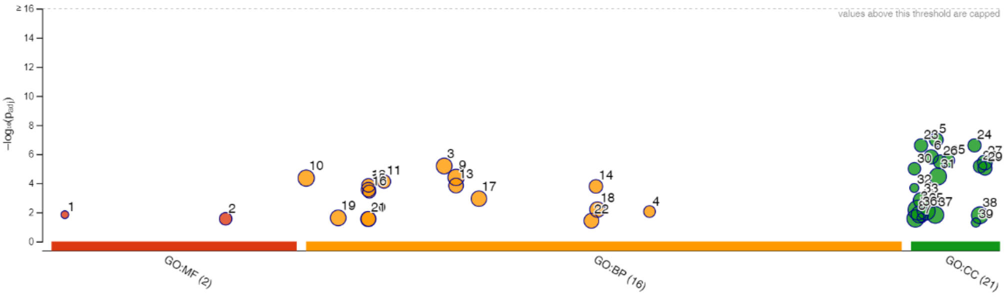

ID	Source	Term ID	Term Name	p_{adj} (query_1)
1	GO:MF	GO:0004127	cytidylate kinase activity	1.438×10^{-2}
2	GO:MF	GO:0051082	unfolded protein binding	2.757×10^{-2}
3	GO:BP	GO:0019953	sexual reproduction	6.509×10^{-6}
4	GO:BP	GO:0061077	chaperone-mediated protein folding	8.783×10^{-3}
5	GO:CC	GO:0031514	motile cilium	1.035×10^{-7}
6	GO:CC	GO:0030141	secretory granule	1.522×10^{-6}
7	GO:CC	GO:0005952	cAMP-dependent protein kinase complex	2.338×10^{-2}
8	GO:CC	GO:0005576	extracellular region	2.662×10^{-2}
9	GO:BP	GO:0022414	reproductive process	3.952×10^{-5}
10	GO:BP	GO:0000003	reproduction	4.423×10^{-5}
11	GO:BP	GO:0009566	fertilization	7.563×10^{-5}
12	GO:BP	GO:0007286	spermatid development	1.391×10^{-4}
13	GO:BP	GO:0022412	cellular process involved in reproduction in multic...	1.438×10^{-4}
14	GO:BP	GO:0048515	spermatid differentiation	1.639×10^{-4}
15	GO:BP	GO:0007281	germ cell development	2.729×10^{-4}
16	GO:BP	GO:0007338	single fertilization	3.719×10^{-4}
17	GO:BP	GO:0032504	multicellular organism reproduction	1.144×10^{-3}
18	GO:BP	GO:0048609	multicellular organismal reproductive process	6.381×10^{-3}
19	GO:BP	GO:0003006	developmental process involved in reproduction	2.390×10^{-2}
20	GO:BP	GO:0007283	spermatogenesis	2.814×10^{-2}
21	GO:BP	GO:0007276	gamete generation	2.860×10^{-2}
22	GO:BP	GO:0048232	male gamete generation	3.767×10^{-2}
23	GO:CC	GO:0005930	axoneme	2.497×10^{-7}
24	GO:CC	GO:0097014	ciliary plasm	2.497×10^{-7}
25	GO:CC	GO:0036126	sperm flagellum	2.740×10^{-6}
26	GO:CC	GO:0032838	plasma membrane bounded cell projection cytopl...	3.352×10^{-6}
27	GO:CC	GO:0099503	secretory vesicle	3.725×10^{-6}
28	GO:CC	GO:0097729	9+2 motile cilium	6.774×10^{-6}
29	GO:CC	GO:0099568	cytoplasmic region	9.144×10^{-6}
30	GO:CC	GO:0001669	acrosomal vesicle	1.003×10^{-5}
31	GO:CC	GO:0031982	vesicle	3.356×10^{-5}
32	GO:CC	GO:0001534	radial spoke	2.089×10^{-4}
33	GO:CC	GO:0005929	cilium	1.469×10^{-3}
34	GO:CC	GO:0005737	cytoplasm	6.915×10^{-3}
35	GO:CC	GO:0012505	endomembrane system	8.007×10^{-3}
36	GO:CC	GO:0005856	cytoskeleton	1.385×10^{-2}
37	GO:CC	GO:0031410	cytoplasmic vesicle	1.495×10^{-2}
38	GO:CC	GO:0097708	intracellular vesicle	1.579×10^{-2}
39	GO:CC	GO:0097224	sperm connecting piece	4.988×10^{-2}

version e111_eg58_p18_30541362
date 22/5/2024, 13:52:54
organism btaurus

g:Profiler

Table S1. Proteins identified in non-capacitated (NC) and *in-vitro* capacitated (IVC) group spermatozoa.

N	Accesion	Protein names	Gene Names	Length	Mass	Peptides (95%)	%Cov(95)	NC (Mean of normalised abundant value)	IVC (Mean of normalised abundant value)	FC (NC/IVC)	adj.p value
1	A6H782	Tektin-3	TEKT3 OMC45	490	56681	45	69,99999881	18126,59855	18770,60695	0,965690592	0,976026651
2	P04394	NADH dehydrogenase [ubiquinone] flavoprotein 2, mitochondrial (EC 7.1.1.2) (NADH	NDUFV2	249	27308	6	32,92999864	558643,775	417313,5781	1,338666663	0,696102603
3	P16368	Metalloproteinase inhibitor 2 (Collagenase inhibitor) (Tissue inhibitor of metalloprot	TIMP2	220	24355	3	13,63999993	246308,9463	36020,39203	6,838041799	6,86457E-07
4	P29392	Spermadhesin-1 (Acidic seminal fluid protein) (ASFP)	SPADH1	134	15036	4	45,51999867	141612,7264	57768,49166	2,451383485	1,57335E-05
5	Q00361	ATP synthase subunit e, mitochondrial (ATPase subunit e) (ATP synthase membrane su	ATP5ME ATP5I	71	8321	4	54,9300015	514168,2518	580502,9876	0,885728864	0,855554095
6	Q02827	NADH dehydrogenase [ubiquinone] 1 subunit C2 (Complex I-B14.5b) (CI-B14.5b) (NAI	NDUFC2	120	14096	2	19,16999966	822481,0917	961581,4147	0,855342126	0,290611247
7	Q148N0	2-oxoglutarate dehydrogenase complex component E1 (E1o) (OGDC-E1) (OGDH-E1) (E	OGDH	1023	115808	39	44,47999895	1415482,632	1647413,337	0,859215232	0,23992354
8	Q28092	Cylicin-2 (Cylicin II) (Multiple-band polypeptide II)	CYLC2 CYL2	488	53562	26	54,71000075	212470,466	101435,7961	2,094630044	0,000431234
9	Q2KJH9	4-trimethylaminobutyraldehyde dehydrogenase (TMABA-DH) (TMABADH) (EC 1.2.1.4	ALDH9A1	494	53977	5	12,74999976	299259,7091	342143,1922	0,874662176	0,403417903
10	Q2YDN8	Serine/threonine-protein kinase VRK3 (EC 2.7.11.22) (Vaccinia-related kinase 3)	VRK3	451	50549	15	42,35000014	2350035,923	1850834,415	1,269717001	0,135366534
11	Q32KP0	Sperm microtubule inner protein 6 (Ciliated bronchial epithelial protein 1) (Spermat	SPMIP6 CBE1 SMRP1	304	35066	37	78,94999981	2603679,225	2896687,458	0,898847136	0,92558903
12	Q32L04	Late cornified envelope-like proline-rich protein 1	LELP1	110	11767	11	83,63999724	113748,7309	121073,188	0,939503888	0,787508604
13	Q32LB7	V-type proton ATPase subunit E 2 (V-ATPase subunit E 2) (Vacuolar proton pump subu	ATP6V1E2	226	26171	3	18,5800001	168050,1045	161062,9138	1,043381747	0,995935621
14	Q32LN6	Protein SPATA31F3 (Protein FAM205C)	SPATA31F3 FAM205C	408	45146	27	44,85000074	4538882,944	2878801,28	1,576657262	0,01395473
15	Q3SWX2	Acyl-coenzyme A thioesterase 9, mitochondrial (Acyl-CoA thioesterase 9) (EC 3.1.2.-) (	ACOT9	437	49853	15	31,58000112	305831,6722	352811,9298	0,866840507	0,183240364
16	Q3SZT4	Testis-expressed protein 29	TEX29	147	16331	9	45,57999969	131591,0301	118588,2369	1,109646568	0,632942695
17	Q3T056	L-lactate dehydrogenase A-like 6B (EC 1.1.1.27)	LDHAL6B	381	41592	27	53,53999734	10414032,2	12864426,07	0,80952171	0,143241486
18	Q9N2J2	Phospholipid hydroperoxide glutathione peroxidase (PHGPx) (EC 1.11.1.12) (Glutath	GPX4	197	22240	82	74,11000133	137891878,1	155544643,6	0,886509975	0,357588717
19	Q9XSJ4	Alpha-enolase (EC 4.2.1.11) (2-phospho-D-glycerate hydro-lyase) (Enolase 1) (HAP47) (	ENO1	434	47326	53	81,33999705	10093752,57	8273925,685	1,219947212	0,330497504
20	A0A3Q1LNB4	Dynein axonemal heavy chain 10	DNAH10 ATP6V0A2	4590	528941	10	2,449000068	193769,3638	153086,456	1,26575119	0,2263211
21	A0A3Q1LPJ9	Disintegrin and metalloproteinase domain-containing protein 20-like		755	85456	16	16,70999974	138804,8789	142696,3674	0,972728889	0,922246589
22	A0A3Q1LQR2	Tubulin alpha chain	TUBA1C	449	49845	68	39,86999989	123975,8121	91324,31958	1,357533378	0,321587532
23	A0A3Q1LUM9	Methylcrotonoyl-CoA carboxylase				2	5,69400005	24295,21143	23616,69571	1,028730341	0,921556871
24	A0A3Q1LYJ4	Histone H2A		116	13051	9	70,09000182	116416,4483	176459,3876	0,659735081	0,137302261
25	A0A3Q1M0U9	Transitional endoplasmic reticulum ATPase (EC 3.6.4.6) (15S Mg(2+)-ATPase p97 subu	VCP	801	89379	12	22,85999954	346400,4416	224950,7036	1,539894902	0,013186267
26	A0A3Q1M2V9	Transmembrane protein 11	TMEM11	257	29481	3	16,33999944	534024,98	599116,6075	0,891353992	0,512766423
27	A0A3Q1M7D1	Dynein axonemal heavy chain 12	DNAH12	3960	454030	12	4,114000127	123225,9911	83573,58006	1,47446108	0,05146089
28	A0A3Q1MA90	Transmembrane protein 190	TMEM190	238	25964	11	27,52000093	14296328,9	13661220,5	1,046489873	0,765897098
29	A0A3Q1MGG8	IQ motif containing N		3340	342049	187	48,80999923	9267442,289	6225120,287	1,488716982	0,010353336
30	A0A3Q1MNG4	BRCA2 and CDKN1A-interacting protein	BCCIP	306	35193	1	6,536000222	210910,3311	266266,9755	0,792100976	0,909921563
31	A0A3Q1MNP8	Dynein light chain roadblock-type 2	DYNLRB2	174	19545	2	16,08999968	269965,311	231214,4269	1,167597172	0,536740196
32	A0A3Q1MNS9	Sperm microtubule associated protein 2 like	SPMAP2L THEGL	457	52248	2	5,110999942	119287,3433	93464,06663	1,27629096	0,188928576
33	A0A3Q1N631	Protein angel homolog 2				1	4,022999853	18752,61849	23906,7056	0,784408308	0,589856937
34	A0A3Q1ND36	Disintegrin and metalloproteinase domain-containing protein 1a-like		716	79840	1	1,338000037	30050,2917	9663,389847	3,109704997	0,002290411
35	A0A3S5ZPN7	Coiled-coil domain containing 116	CCDC116	677	75049	2	4,484000057	114302,1964	71472,81982	1,599240056	0,028478525
36	A0A452DI02	Acyl-protein thioesterase 1 (EC 3.1.2.22) (Lysophospholipase 1) (Lysophospholipase I	LYPLA1	219	23575	3	17,35000014	351862,8689	360732,8846	0,975411125	0,887855462
37	A0A4W2BMT5	Protein interacting with cyclin A1	PROCA1	376	42242	4	14,10000026	28043,06506	29945,38891	0,936473563	0,812227059
38	A0A4W2BMW7	IQ domain-containing protein F5	LOC113880958 IQCF5	149	18051	2	12,74999976	1645934,213	1705692,622	0,964965312	0,935160425
39	A0A4W2BP90	Protein jagunal homolog 1	JAGN1	183	21102	2	7,103999704	30277,8415	37068,82713	0,816800634	0,428379247
40	A0A4W2BQ23	Lysozyme like 4	LYZL4	145	16293	11	70,34000158	652832,7611	670337,0825	0,973887285	0,873954779
41	A0A4W2BRF2	Mitochondrial import inner membrane translocase subunit Tim21		244	27962	1	5,327999964	32383,23257	37558,76644	0,862201708	0,56761005
42	A0A4W2BT41	Epididymal-specific lipocalin-5-like	LOC113901868	180	19569	2	15,0000006	44587,8363	16616,19348	2,683396552	0,00150306
43	A0A4W2BT96	ATP synthase subunit alpha		588	63130	91	64,63000178	155219,7816	167723,6339	0,92544967	0,915883223
44	A0A4W2BTQ2	Kinesin-like protein		854	97297	2	3,62999998	39869,35485	29592,8778	1,347261835	0,178188845
45	A0A4W2BU14	Glycerophosphodiester phosphodiesterase 1	GDE1	331	37653	3	10,57000011	341706,7858	415429,5262	0,822538515	0,261388431
46	A0A4W2BU38	Rieske domain-containing protein		464	50631	9	20,03999949	1292178,051	1562450,854	0,827019966	0,257814642
47	A0A4W2BUW4	Vesicle-associated membrane protein-associated protein A	VAPA	294	32454	14	27,21000016	427171,2676	232427,3769	1,837869847	0,001306308
48	A0A4W2BV22	Septin-4	SEPTIN4	477	54875	3	8,595000207	113849,1459	80997,86158	1,405582119	0,227927877
49	A0A4W2BVV1	Adenylate kinase 8 (EC 2.7.4.3) (EC 2.7.4.6) (ATP-AMP transphosphorylase 8)	AK8	498	56728	10	26,51000023	746966,7879	506309,9567	1,475315226	0,018397256
50	A0A4W2BVW5	Proteasome assembly chaperone 2	PSMG2	264	29224	2	9,091000259	29172,88572	39898,33838	0,731180468	0,470737173
51	A0A4W2BWR0	Sperm-associated microtubule inner protein 5 domain-containing protein	C26H10orf82	283	31820	9	37,09999919	994742,2893	1050324,321	0,947081078	0,644203165
52	A0A4W2BWY0	Voltage-dependent anion-selective channel protein 3	VDAC3	283	30739	64	74,55999851	35298497,3	38969163,61	0,905805874	0,583381185
53	A0A4W2BZ57	Carnitine O-palmitoyltransferase 1, muscle isoform (EC 2.3.1.21) (Carnitine O-palmit	CPT1B	771	88512	21	22,17999995	706955,0591	747429,0071	0,945849107	0,674721891
54	A0A4W2BZV4	Radial spoke head protein 9 homolog	RSPH9	276	31294	12	43,84000003	1087875,474	962945,0571	1,129737846	0,855554095
55	A0A4W2C0V1	Cytochrome c1, heme protein, mitochondrial	LOC113903907 CYC1	325	35297	21	52,92000175	1883733,424	1775641,486	1,060874866	0,749494315
56	A0A4W2C0W3	EF-hand domain containing 1	EFHC1	640	74004	23	38,74999881	1187395,763	1172142,026	1,013013557	0,900271027
57	A0A4W2C225	GRAM domain-containing protein		313	31966	16	29,3900013	2084079,478	1361194,914	1,531066166	0,297900975
58	A0A4W2C4M9	Aspartate aminotransferase (EC 2.6.1.1)	GOT1	413	46399	4	11,86000034	23696,71072	21266,89696	1,114253328	0,975027051
59	A0A4W2C4X4	Dynein axonemal heavy chain 17	DNAH17	4462	509199	81	21,78000063	26198,45857	42402,68085	0,617849109	0,300356268
60	A0A4W2C5D2	CDGSH iron sulfur domain 1	CISD1	106	11983	3	21,69999927	33274,72895	38983,13054	0,853567389	0,698933149

61	A0A4W2C6J1	Leucine zipper protein 2	LUZP2	340	38973	55	57,64999986	3580591,747	2473033,383	1,447854191	0,126671924
62	A0A4W2C6Q1	Cytochrome c oxidase subunit (Cytochrome c oxidase polypeptide VIa)	GATC	126	14105	3	46,83000147	66988,67189	63942,7627	1,047634933	0,727036926
63	A0A4W2C6R9	Adenine phosphoribosyltransferase (EC 2.4.2.7)		246	26442	2	5,285000056	9418,325488	7530,522198	1,250686903	0,632942695
64	A0A4W2C6V1	Ammonium transporter Rh type C (Rhesus blood group family type C glycoprotein)		598	65278	2	6,019999832	69316,64792	33665,92686	2,058955578	0,05317512
65	A0A4W2C855	SSD domain-containing protein	LOC113906256	933	103629	5	10,18000022	39910,07321	30002,34955	1,330231592	0,157649312
66	A0A4W2C8I6	Phosphatidylethanolamine-binding protein 1 (HCNPpp)	PEBP1	187	20986	10	75,94000101	674323,8468	427523,1619	1,577280266	0,047101558
67	A0A4W2CCN9	Apolipoprotein A-I (Apolipoprotein A1)		207	23868	1	6,763000041	14192,28842	20106,84998	0,705843453	0,196201538
68	A0A4W2CDD2	Glucose-6-phosphate isomerase (EC 5.3.1.9)	GPI	565	63636	14	27,61000097	547271,9044	449254,3728	1,218178247	0,221081341
69	A0A4W2CDK1	ATP synthase subunit gamma	ATP5F1C	298	33072	29	68,45999956	2868719,981	3370779,661	0,851055325	0,2263211
70	A0A4W2CE44	Heat shock protein family B (small) member 9	HSPB9	157	16784	15	68,15000176	2631196,992	2032372,081	1,294643347	0,089467949
71	A0A4W2CEF3	Glyceraldehyde-3-phosphate dehydrogenase (EC 1.2.1.12)	GAPDHS	403	44104	141	87,84000278	30651771,7	25782835,34	1,188844101	0,15596884
72	A0A4W2CF85	Calpain small subunit 1	CAPNS1	263	27931	1	7,224000245	38598,41101	28851,87804	1,337812774	0,564883411
73	A0A4W2CG68	Leucine-rich repeat-containing protein 51	LRRC51	192	22107	3	26,03999972	76576,37463	78399,10162	0,976750665	0,784303194
74	A0A4W2CHI3	Cytochrome c oxidase subunit	LOC113875989 COX6B2	88	10533	10	90,90999961	2885871,658	3236675,576	0,891615978	0,491080975
75	A0A4W2CHJ7	Septin-10		487	56824	4	11,08999997	92435,44536	82437,38175	1,121280703	0,939494502
76	A0A4W2CIX0	Fibronectin type-II domain-containing protein		183	21292	16	34,97000039	3139030,5	2143760,378	1,464263699	0,230253432
77	A0A4W2CJE1	Uncharacterized protein		196	23234	3	24,49000031	39548,97643	39715,50021	0,995807083	0,939494502
78	A0A4W2CKE1	Protein FAM24A		97	10404	3	63,91999722	94277,49551	130991,6415	0,71972146	0,157790295
79	A0A4W2CMS0	Luteinizing hormone subunit beta	LHB	338	37441	2	7,987999916	23137,97977	34700,23013	0,666796147	0,263093664
80	A0A4W2CN20	Tubulin alpha-4A chain		1073	120740	105	24,42000061	486305,3923	372055,0638	1,307079085	0,061427518
81	A0A4W2CP64	Destrin (Actin-depolymerizing factor)	DSTN	165	18506	1	6,667000055	75461,52827	97822,59438	0,771412052	0,102428241
82	A0A4W2CPA4	RGS domain-containing protein	RGS22	1254	145932	4	4,78500016	15294,66346	10795,71537	1,416734597	0,301082145
83	A0A4W2CPR2	Eukaryotic initiation factor 4A-I (EC 3.6.4.13) (ATP-dependent RNA helicase eIF4A-1)	EIF4A1	406	46154	2	7,389000058	46158,60497	36072,40208	1,279609959	0,508231288
84	A0A4W2CQX1	ATP-dependent (S)-NAD(P)H-hydrate dehydratase (EC 4.2.1.93) (ATP-dependent NAD(	NAXD CARKD	345	36103	2	9,85499993	83538,00577	61617,94262	1,355741562	0,351036391
85	A0A4W2CR59	Proteasome subunit beta	PSMB6	239	25542	5	33,88999999	284613,2317	395289,6846	0,720011786	0,065711753
86	A0A4W2CS05	Vesicle-associated membrane protein 3 (Synaptobrevin-3)	VAMP3	148	15514	2	16,22000039	91693,47622	71062,10375	1,29032876	0,297900975
87	A0A4W2CSR1	Cytochrome c oxidase subunit 6C (Cytochrome c oxidase polypeptide VIc)	LOC113904380	74	8610	6	37,83999979	4406531,598	6094765,007	0,723002707	0,047002192
88	A0A4W2CTQ1	Aspartate aminotransferase (EC 2.6.1.1)	GOT2	454	50363	14	34,36000049	1421024,363	1756734,416	0,808901078	0,181236373
89	A0A4W2CUA3	GTP:AMP phosphotransferase AK3, mitochondrial (EC 2.7.4.10) (Adenylate kinase 3) (	AK3	227	25671	6	26,42999887	385763,7204	476134,7446	0,810198635	0,113310356
90	A0A4W2CUP5	Ubiquitin-like domain-containing protein		1146	128851	10	70,6799984	728842,3941	655710,8008	1,111530256	0,975027051
91	A0A4W2CVV1	Mannose-6-phosphate isomerase (EC 5.3.1.8)	MPI	423	46369	4	10,86999997	91085,31115	56421,16826	1,61438187	0,145447082
92	A0A4W2CWK5	ATP synthase F1 subunit epsilon	ATP5F1E	51	5783	3	45,1000005	332636,857	411256,9027	0,808829845	0,191863076
93	A0A4W2CXU5	Casein kinase II subunit alpha (CK II alpha) (EC 2.7.11.1)	CSNK2A1	391	45144	11	34,0200007	304689,865	212216,9632	1,435746985	0,126671924
94	A0A4W2CYV7	NADH dehydrogenase [ubiquinone] 1 alpha subcomplex subunit 7 (Complex I-B14.5a	NDUFA7	113	12677	2	16,80999994	31842,99036	32282,25976	0,986392855	0,993202671
95	A0A4W2CZ27	Coiled-coil-helix-coiled-coil-helix domain containing 3	CHCHD3	232	26685	2	9,052000195	7230,3069	5178,92657	1,396101451	0,149930287
96	A0A4W2CZJ1	LY6/PLAUR domain containing 4	LYPD4	246	26824	3	16,67000055	25650,93623	22465,65473	1,141784494	0,99108826
97	A0A4W2D0C7	cAMP-dependent protein kinase type I-alpha regulatory subunit	PRKAR1A	380	42893	11	37,63000071	1102925,17	669211,2615	1,648097146	0,017424933
98	A0A4W2D0I6	Sperm acrosome associated 1	SPACA1	297	32850	26	46,45999968	10655971,24	7815000,438	1,363527913	0,05146089
99	A0A4W2D0I8	H1.7 linker histone		265	29159	4	17,73999929	172490,7195	160883,5049	1,072146704	0,915883223
100	A0A4W2D126	Superoxide dismutase [Mn], mitochondrial (EC 1.15.1.1)		556	59294	13	23,01999927	449222,2263	514431,9308	0,873239392	0,391863779
101	A0A4W2D1Z5	Uncharacterized protein		5914	656236	54	14,00000006	764325,1568	536103,1224	1,425705475	0,038709139
102	A0A4W2D277	Clusterin	CLU	439	51114	21	30,30000031	1583528,439	415565,6292	3,810537562	3,29919E-05
103	A0A4W2D2C2	NADH-ubiquinone oxidoreductase 75 kDa subunit, mitochondrial		875	96701	23	33,59999955	2391025,521	2658818,027	0,899281371	0,391863779
104	A0A4W2D2D8	Transmembrane protein 38B	TMEM38B	291	32451	12	35,40000021	1072668,411	1040586,345	1,030830758	0,993202671
105	A0A4W2D3D4	Calmodulin 1	CALM1	365	38625	17	26,85000002	159201,4586	85544,96334	1,861026673	0,139456822
106	A0A4W2D4I1	Calcium/calmodulin dependent protein kinase IV	CAMK4	463	49902	3	10,58000028	72178,15847	63858,28494	1,130286517	0,588531215
107	A0A4W2D4S5	Lon protease homolog, mitochondrial (EC 3.4.21.53) (Lon protease-like protein) (LOI	LONP1	1027	113918	19	28,2400012	941682,6465	949655,9286	0,991604031	0,980045196
108	A0A4W2D592	Fibrous sheath-interacting protein 2 C-terminal domain-containing protein		6744	759927	129	22,87999988	24561,251	20903,38844	1,174988977	0,508178477
109	A0A4W2D5V1	NADH dehydrogenase [ubiquinone] 1 alpha subcomplex subunit 12		220	25453	5	29,55000103	85896,08325	95721,59789	0,897353211	0,508178477
110	A0A4W2D819	14-3-3 protein zeta/delta	YWHAZ	229	26085	12	48,46999943	521756,7771	382542,4894	1,363918497	0,028458364
111	A0A4W2D8B4	SPATA31/FAM205 domain-containing protein		1254	138951	9	8,453000337	15843,87169	11392,99605	1,390667707	0,723696372
112	A0A4W2D8G0	Sperm equatorial segment protein 1	SPESP1	345	38997	39	63,19000125	3480356,214	2657034,98	1,309864657	0,088790258
113	A0A4W2D8T3	Albumin	ALB	655	74084			107375,7403	20255168,23	0,005301153	
114	A0A4W2D9A8	SPATA31 domain-containing protein		1523	173961	23	18,44999939	620611,7888	355955,6757	1,743508619	0,000769974
115	A0A4W2D9T4	Testis expressed 46	TEX46	162	18816	1	8,64199996	28910,84135	26656,40774	1,084573797	0,829647132
116	A0A4W2DBJ7	dTMP kinase (EC 2.7.4.9)	DTYMK	228	25491	1	5,702000111	30773,73621	23783,08029	1,293934	0,596824829
117	A0A4W2DDE0	Ring finger protein 130	RNF130	462	50538	1	3,030000068	20561,69111	14116,55187	1,456566115	0,579112446
118	A0A4W2DER0	High mobility group box 4	HMGB4	247	28855	4	12,96000034	78715,7862	89033,24557	0,884116778	0,742505556
119	A0A4W2DFR9	Ferritin		157	17860	13	63,69000077	2377454,472	3028333,773	0,785070158	0,21776382
120	A0A4W2DHN1	Medium-chain specific acyl-CoA dehydrogenase, mitochondrial (EC 1.3.8.7)	ACADM	421	46573	31	48,21999967	1771494,237	1820254,405	0,973212444	0,956801148
121	A0A4W2DIS9	IF rod domain-containing protein		561	60110	9	12,82999963	358099,5227	555964,6974	0,644104786	0,23992354
122	A0A4W2DLZ3	Actin-like protein 7A	ACTL7A	438	49016	43	79,68000174	2744923,21	2048324,678	1,340082088	0,135366534
123	A0A4W2DMG8	Endoplasmin (94 kDa glucose-regulated protein) (Heat shock protein 90 kDa beta mer		808	92862	4	9,15800035	386421,1793	265712,9868	1,454280365	0,455287943
124	A0A4W2DNN5	Isoaspartyl peptidase/L-asparaginase (EC 3.4.19.5) (EC 3.5.1.1) (Asparaginase-like pro	ASRGL1	308	32050	27	66,87999964	5037128,188	5088432,23	0,989917515	0,984853837

125	A0A4W2DNW3	RIIa domain-containing protein		382	41488	26	49,73999858	121649,2731	76462,03781	1,590976079	0,017424933
126	A0A4W2DQ38	IQ motif containing F1	IQCF1	209	24194	14	38,76000047	242529,0955	128519,7401	1,887096063	0,028458364
127	A0A4W2DQI3	Betaine--homocysteine S-methyltransferase	BHMT	538	59271	2	4,833000153	48442,27912	43124,73176	1,123306213	0,457406445
128	A0A4W2DRC1	Spermatid-specific manchette-related protein 1		166	19396	15	69,88000274	230960,5225	250178,1007	0,923184411	0,952134592
129	A0A4W2DS83	Nucleoside diphosphate kinase (EC 2.7.4.6)	LOC113883266 DECR2	187	20457	3	19,78999972	22404,9704	23907,24015	0,937162561	0,66938158
130	A0A4W2DSH2	NADH dehydrogenase [ubiquinone] 1 alpha subcomplex subunit 10, mitochondrial	NDUFA10	343	39278	11	38,78000081	772922,8595	914107,4556	0,845549235	0,236159715
131	A0A4W2DSL6	Arylsulfatase A	ARSA	507	53807	8	21,29999995	269184,7593	218905,2412	1,229686223	0,748316576
132	A0A4W2DTA2	Mitochondrial glutamate carrier 1 (Glutamate/H(+) symporter 1) (Solute carrier famil	SLC25A22	322	34531	3	21,43000066	81099,76263	78316,27981	1,035541561	0,980534154
133	A0A4W2DTM9	Phosphoglycerate kinase (EC 2.7.2.3)		417	44781	40	75,7799983	3038516,64	2195305,759	1,384097239	0,038756378
134	A0A4W2DTV6	S-formylglutathione hydrolase (EC 3.1.2.12)	ESD	282	31548	1	5,319000036	70582,38314	57420,74701	1,229213948	0,331502239
135	A0A4W2DTX9	Secretoglobin family 1D member		123	13632	2	26,82999969	18768,66894	3456,309386	5,43026299	0,000525961
136	A0A4W2DVI7	NADPH:adrenodoxin oxidoreductase, mitochondrial (EC 1.18.1.6)		463	50858	2	7,126999646	68501,04421	99536,58173	0,688199685	0,126671924
137	A0A4W2DW14	Multifunctional fusion protein [Includes: Delta-1-pyrroline-5-carboxylate dehydroge	ALDH4A1	563	61495	10	25,40000081	565219,608	658630,6069	0,858173917	0,258073581
138	A0A4W2DY45	cAMP-dependent protein kinase type II-alpha regulatory subunit	PRKAR2A	401	45094	21	50,37000179	2299077,484	1804079	1,274377388	0,126671924
139	A0A4W2DYA7	Triosephosphate isomerase (EC 5.3.1.1)	TPI1	286	30577	55	80,41999936	12839656,45	12971651,47	0,989824348	0,977014723
140	A0A4W2DYH1	Isocitrate dehydrogenase [NADP] (EC 1.1.1.42)	IDH2	477	53617	17	35,85000038	954796,3541	1133409,029	0,842411106	0,149930287
141	A0A4W2DYQ2	Actin, cytoplasmic 1 (Beta-actin)	ACTB	375	41737	23	44,26999986	1357624,64	954481,6696	1,422368478	0,067550814
142	A0A4W2DZI5	EF-hand domain-containing protein	EFCAB6	1497	172742	41	29,58999872	2013047,497	1823535,992	1,103925289	0,297900975
143	A0A4W2E067	Serine/threonine-protein phosphatase (EC 3.1.3.16)	PPP1CC	337	38548	35	68,83999705	372241,8841	304713,7105	1,221611865	0,249343849
144	A0A4W2E1I7	Uncharacterized protein		473	52661	3	10,57000011	84244,50518	64711,87333	1,30184	0,389687067
145	A0A4W2E5S0	folate gamma-glutamyl hydrolase (EC 3.4.19.9)	GGH	318	35683	2	7,547000051	1937,952827	1612,669947	1,201704559	0,855554095
146	A0A4W2E5V3	Golgi associated RAB2 interactor protein-like Rab2B-binding domain-containing pro		585	63860	20	32,31000006	860291,0669	777902,4887	1,10591119	0,566781527
147	A0A4W2E851	Coiled-coil-helix-coiled-coil-helix domain containing 6	CHCHD6	337	37062	2	7,417999953	37960,43167	34985,81588	1,085023479	0,956801148
148	A0A4W2E8C9	Prostamide/prostaglandin F synthase (EC 1.11.1.20) (Peroxiredoxin-like 2B)	PRXL2B	210	23526	1	5,237999931	18560,10879	22083,42965	0,840454091	0,454341614
149	A0A4W2E975	Cilia- and flagella-associated protein 20	CFAP20	193	22748	13	66,32000208	1608532,955	1448012,567	1,110855659	0,644203165
150	A0A4W2E9B9	Tubulin beta chain	TUBB4B	445	49831	199	84,71999764	1593434,884	1593122,908	1,000195826	0,857569767
151	A0A4W2E9V4	ADAM metallopeptidase domain 20		744	83691	6	9,811999649	196694,7333	202402,4048	0,971800377	0,89270578
152	A0A4W2EA76	IF rod domain-containing protein		428	47264	7	18,21999997	17461,51031	10371,22568	1,683649632	0,498549681
153	A0A4W2ECX5	Fibronectin type-II domain-containing protein		356	41036	13	33,9899987	2442485,252	1547833,01	1,578003076	0,225338155
154	A0A4W2EDB5	NADH dehydrogenase [ubiquinone] iron-sulfur protein 6, mitochondrial	NDUFS6	124	13413	6	61,29000187	18976,42812	21741,27113	0,872829745	0,358725486
155	A0A4W2EE11	Beta-defensin		101	11774	2	21,78000063	19842,40982	18908,63264	1,049383644	0,789772879
156	A0A4W2EEF6	Aconitate hydratase, mitochondrial (Aconitase) (EC 4.2.1.3)	ACO2	780	85359	61	49,73999858	5126376,24	6021528,562	0,851341348	0,263093664
157	A0A4W2EF91	BPTI/Kunitz inhibitor domain-containing protein		138	15824	3	17,38999933	514434,7267	463108,9339	1,110828769	0,632479291
158	A0A4W2EHT0	T-complex protein 1 subunit beta (CCT-beta)	CCT2	526	56405	10	31,56000078	184329,5934	151934,5044	1,213217459	0,508178477
159	A0A4W2EL77	ATP synthase subunit beta (EC 7.1.2.2)	ATP5F1B	528	56284	105	77,4600029	52740855,62	67247729,65	0,784277118	0,040494042
160	A0A4W2ELI9	tripeptidyl-peptidase II (EC 3.4.14.10)		1297	143824	3	3,084000014	46277,80994	27199,56127	1,701417515	0,375311421
161	A0A4W2ELT9	NADH dehydrogenase [ubiquinone] 1 alpha subcomplex subunit 8 (Complex I-19kD) (	NDUFA8	278	31103	7	29,49999869	601312,6589	650038,8997	0,925041039	0,664034446
162	A0A4W2EMT1	Dynein regulatory complex subunit 2 (Coiled-coil domain-containing protein 65)	CCDC65	498	58814	2	5,823000148	18155,97105	12691,76782	1,430531295	0,748316576
163	A0A4W2EMY6	Potassium voltage-gated channel interacting protein 3	LOC113900915	341	37632	5	22,29000032	378165,8345	457525,8958	0,826545203	0,373978461
164	A0A4W2ENE8	Serine/threonine-protein phosphatase (EC 3.1.3.16)	PPP1CB	445	49621	29	34,830001	28323,05832	36587,62719	0,774115746	0,204107047
165	A0A4W2EPP2	Glutamate dehydrogenase 1, mitochondrial (EC 1.4.1.3)	GLUD1	561	61631	2	5,703999847	56850,26757	70974,28987	0,800998047	0,179330148
166	A0A4W2EPS9	Long-chain-fatty-acid--CoA ligase ACSBG2 (EC 6.2.1.15) (EC 6.2.1.3) (Acyl-CoA synthet		678	75715	9	19,47000027	382571,6628	356167,0966	1,074135333	0,819248701
167	A0A4W2ERA1	Actin-like protein 7B	ACTL7B	417	45563	14	37,88999915	974793,5017	703345,9316	1,3859375	0,143973176
168	A0A4W2ERC1	Acyl-CoA thioester hydrolase/bile acid-CoA amino acid N-acetyltransferase domain-c	ACOT4	452	50576	6	13,05000037	366875,4227	273728,8482	1,340287753	0,126671924
169	A0A4W2ERW4	Multifunctional fusion protein [Includes: Poly [ADP-ribose] polymerase (PARP) (EC 2.	PARP6	926	102497	42	31,8599999	916734,7273	883272,1672	1,037884767	0,869709015
170	A0A4W2ERZ2	ATP synthase subunit b	ATP5PB	256	28806	17	42,96999872	1206688,227	1515626,856	0,796164453	0,195027592
171	A0A4W2EU79	Proteasome subunit alpha type	PSMA3	266	29875	7	27,43999958	141987,5105	191286,9824	0,74227482	0,080450779
172	A0A4W2EUH8	Cytochrome c 2	LOC113901903	105	11740	8	55,23999929	1049747,336	772544,66	1,358817671	0,282121058
173	A0A4W2EV06	ATP synthase F1 subunit delta	ATP5F1D	168	17612	12	57,74000287	744604,8457	950260,5542	0,783579664	0,456696907
174	A0A4W2EVB5	Uncharacterized protein		350	37917	46	63,70999813	22276398,29	14833322,96	1,50178071	0,00354176
175	A0A4W2EX92	F-actin-capping protein subunit beta	CAPZB	301	33741	62	76,41000152	2992390,403	2826189,622	1,058807371	0,681095177
176	A0A4W2EY09	Glycerol-3-phosphate dehydrogenase (EC 1.1.5.3)	GPD2	727	80797	42	61,21000051	4573398,893	5162404,782	0,885904745	0,2651476
177	A0A4W2EYG8	Cytochrome b-c1 complex subunit 6	LOC113889946	91	10624	4	61,54000163	238339,3621	241945,5922	0,985094872	0,995935621
178	A0A4W2EYI0	ATP synthase subunit O, mitochondrial (ATP synthase peripheral stalk subunit OSCP)	ATP5PO	213	23320	24	65,72999954	1376326,534	1724326,07	0,798182292	0,075795261
179	A0A4W2EZ36	Superoxide dismutase [Cu-Zn] (EC 1.15.1.1)		330	35056	4	13,63999993	366258,9276	268945,2761	1,361834396	0,373978461
180	A0A4W2F0A5	NAD(P)H-hydrate epimerase (EC 5.1.99.6) (Apolipoprotein A-I-binding protein) (AI-BP	APOA1BP AIBP	363	38374	3	14,59999979	97413,05953	112365,7089	0,866928714	0,609426122
181	A0A4W2F1D4	cAMP-dependent protein kinase catalytic subunit alpha (EC 2.7.11.11)	PRKACA	343	39889	14	30,89999855	985349,4103	770238,8418	1,279277747	0,126671924
182	A0A4W2F2N0	Uncharacterized protein	NDUFV3	447	48351	5	20,8100006	46957,8893	58640,00926	0,800782433	0,79484739
183	A0A4W2F3T3	Solute carrier family 25 member 3 (Phosphate carrier protein, mitochondrial) (Phosp	SLC25A3	361	40004	10	28,52999866	701516,9977	958416,3153	0,731954357	0,208704758
184	A0A4W2F4K2	ATP synthase subunit d, mitochondrial	ATP5PD	161	18692	14	77,64000297	2475361,136	2594743,622	0,953990643	0,829647132
185	A0A4W2F4Y9	Cytochrome c oxidase subunit 7C, mitochondrial (Cytochrome c oxidase polypeptide	LOC113900450	63	7331	1	14,2900005	17531,61767	23285,15225	0,752909729	0,357918821
186	A0A4W2F5L1	galactokinase (EC 2.7.1.6) (Galactose kinase)	GALK1	392	42265	3	10,19999981	230367,813	185047,1426	1,244914187	0,698933149
187	A0A4W2F767	Transmembrane and coiled-coil domains 2	TMCO2	179	19952	3	16,76000059	5402,187196	6060,266245	0,891410868	0,610611446
188	A0A4W2F7C1	NADH dehydrogenase [ubiquinone] 1 beta subcomplex subunit 11, mitochondrial (C	NDUFB11	154	17573	3	22,07999974	66350,44001	68996,05232	0,961655599	0,771775159

189	A0A4W2F836	Sodium/potassium-transporting ATPase subunit beta	ATP1B3	279	31521	3	10,39000005	343236,3277	377959,9698	0,908128784	0,694405834
190	A0A4W2F8A2	NADH dehydrogenase [ubiquinone] 1 alpha subcomplex subunit 6 (Complex I-B14) (N	NDUFA6	154	17799	1	7,142999768	22398,25481	21742,50563	1,03015978	0,961830278
191	A0A4W2F904	Dynein axonemal intermediate chain 7	DNAI7	721	83769	2	3,051000088	69251,50463	56439,65065	1,227000944	0,491080975
192	A0A4W2F919	GLIPR1 like 1	GLIPR1L1	241	27110	12	34,43999887	256268,4199	143960,3095	1,780132461	0,002083183
193	A0A4W2F9T8	RIB43A domain with coiled-coils 2	RIBC2	381	45169	6	19,68999952	179305,8682	191215,7185	0,937715109	0,833283006
194	A0A4W2F9Z4	Adenylate kinase 9	AK9	1902	220085	4	2,680999972	138992,3118	97410,60293	1,42687046	0,157790295
195	A0A4W2FA84	Prohibitin	PHB1	272	29804	18	64,34000134	3323355,349	4081865,826	0,814175549	0,230253432
196	A0A4W2FA94	Complex III subunit 9	UQCR10 LOC113907334	64	7458	3	50	984708,9701	1425687,819	0,690690456	0,047101558
197	A0A4W2FB35	Dynein axonemal heavy chain 1	DNAH1	4266	487581	14	3,962000087	293602,8397	232515,1383	1,262725695	0,07942107
198	A0A4W2FBC3	Phosphoinositide phospholipase C (EC 3.1.4.11)	PLCZ1	637	74100	19	30,30000031	2878688,951	2193340,338	1,312467974	0,126696309
199	A0A4W2FCM2	NADH dehydrogenase [ubiquinone] iron-sulfur protein 7, mitochondrial (Complex I-2	NDUFS7	216	23771	4	24,53999966	127637,3938	134319,3001	0,950253565	0,740874217
200	A0A4W2FCS9	10 kDa heat shock protein, mitochondrial (10 kDa chaperonin) (Chaperonin 10)	LOC113905824	259	27646	10	30,12000024	1018243,744	1266823,716	0,803776982	0,177243867
201	A0A4W2FD88	Protein FAM161A	FAM161A	721	84200	8	15,95000029	212736,6413	132722,9695	1,602862279	0,038463698
202	A0A4W2FDF0	T-complex protein 1 subunit delta	CCT4	545	58389	14	30,27999997	530680,5891	377645,6119	1,405234358	0,047101558
203	A0A4W2FDX9	T-complex protein 1 subunit eta (TCP-1-eta) (CCT-eta)	CCT7	543	59443	10	29,10000086	98277,95376	84176,65747	1,167520269	0,566742372
204	A0A4W2FE87	Malate dehydrogenase (EC 1.1.1.37)	MDH1	245	27009	2	11,84	328563,2393	278540,6156	1,179588257	0,333283194
205	A0A4W2FED5	Cytochrome b-c1 complex subunit 8 (Complex III subunit 8)	UQCRQ LOC113895381	82	9720	4	56,09999895	272779,7809	178345,5264	1,529501672	0,051802887
206	A0A4W2FEJ5	Cytochrome c oxidase subunit 4	COX4I1 LOC113875460	169	19572	14	60,36000252	7849715,708	11189571,13	0,701520694	0,094337531
207	A0A4W2FEV1	IQ motif containing F2	IQCF2	163	19536	1	5,521000177	195626,6261	100769,672	1,941324429	0,04467807
208	A0A4W2FF31	Mitochondria-eating protein (Spermatogenesis-associated protein 18)	SPATA18	557	62902	23	46,50000036	4030003,039	3914776,686	1,029433698	0,938963059
209	A0A4W2FF64	Armadillo like helical domain containing 1	ARMH1	442	49408	3	6,108999997	31889,85872	34502,23767	0,924283782	0,932410033
210	A0A4W2FFW8	Reactive oxygen species modulator 1 (Protein MGR2 homolog)	ROMO1	79	8183	2	21,5200007	59985,16855	64161,60107	0,934907601	0,829683458
211	A0A4W2FG39	OCIA domain-containing protein 1	OCIAD1	220	24540	3	10,00000015	178201,3385	175123,9268	1,017572766	0,934241576
212	A0A4W2FG68	Malate dehydrogenase (EC 1.1.1.37)	MDH2	338	35668	41	70,41000128	14572846,89	16823664,14	0,866211235	0,111227032
213	A0A4W2FGL7	Protein FAM162A	FAM162A	156	17737	1	7,69200027	47538,54935	57560,04048	0,825894995	0,842108033
214	A0A4W2FHP1	Cytochrome c oxidase subunit 5A, mitochondrial (Cytochrome c oxidase polypeptid	LOC113879686	152	16735	20	69,73999739	1349601,306	1692908,577	0,797208617	0,177243867
215	A0A4W2FHT1	Cytochrome b-c1 complex subunit 7 (Complex III subunit VII) (Ubiquinol-cytochrom	LOC113904403	160	19070	9	36,25000119	527570,7483	621301,7612	0,8491377	0,439676506
216	A0A4W2FIJ7	Protein FAM205A-like	LOC113897149	1287	142386	8	5,283999816	473575,7415	449334,3443	1,053949576	0,696820982
217	A0A4W2FIR4	SPATA31/FAM205 domain-containing protein		1388	153579	28	29,8299998	3523594,622	2659036,089	1,325139826	0,05146089
218	A0A4W2FJA4	ADP/ATP translocase (ADP,ATP carrier protein)	SLC25A31	323	35754	41	83,27999711	67884,04134	63300,04634	1,07241693	0,856205159
219	A0A4W2FJN7	NADH dehydrogenase [ubiquinone] 1 beta subcomplex subunit 8, mitochondrial (Co	NDUFB8	186	21684	4	26,87999904	29337,19642	34945,11152	0,839522186	0,428559287
220	A0A4W2FL75	Cilia and flagella associated protein 77	CFAP77	284	32907	7	27,45999992	48156,16923	69441,05661	0,693482668	0,422463852
221	A0A4W2FNN4	NADH dehydrogenase [ubiquinone] 1 alpha subcomplex subunit 11 (Complex I-B14.7	NDUFA11	141	14788	2	24,10999984	435721,7114	457542,4262	0,952308871	0,89270578
222	A0A4W2FPB2	Hydroxysteroid 17-beta dehydrogenase 10	HSD17B10	288	30151	13	67,71000028	777973,0317	867551,2447	0,896745911	0,454341614
223	A0A4W2FPY7	Ubiquitin carboxyl-terminal hydrolase (EC 3.4.19.12)	UCHL1	339	37946	3	18,5800001	12122,22989	9463,908534	1,280890432	0,696102603
224	A0A4W2FQC5	Glutathione peroxidase	GPX5	219	25049	10	40,18000066	156083,4084	76328,96948	2,044877711	0,120157951
225	A0A4W2FQT7	Proteasome subunit alpha type	PSMA4	261	29484	5	21,45999968	171763,5649	269500,6394	0,637340102	0,124717001
226	A0A4W2FQT8	Electron transfer flavoprotein-ubiquinone oxidoreductase (ETF-QO) (EC 1.5.5.1)	ETFDH	617	68612	10	24,63999987	248914,8026	277108,0008	0,898259169	0,879881371
227	A0A4W2FRD0	Thioredoxin domain containing 8		127	14647	4	37,79999912	1515229,379	1563036,455	0,969413973	0,829647132
228	A0A4W2FS86	Peroxiredoxin-5 (EC 1.11.1.24)	PRDX5	219	23253	19	55,7099998	3418675,488	3079881,663	1,110002221	0,620534183
229	A0A4W2FTM0	Diazepam-binding inhibitor-like 5	LOC113877332	87	9788	12	94,24999952	99575,11861	108779,4412	0,915385458	0,664034446
230	A0A4W2FUK5	Adenylate kinase 2, mitochondrial (AK 2) (EC 2.7.4.3) (ATP-AMP transphosphorylase 2	AK2	308	33534	2	12,98999935	39796,49093	32994,1511	1,206168051	0,607981468
231	A0A4W2FUX7	Serine/threonine-protein phosphatase 2A activator (EC 5.2.1.8) (Phosphotyrosyl phc	PTPA	295	33617	1	9,831000119	8207,263879	7610,69546	1,078385533	0,640712455
232	A0A4W2FV49	Voltage-dependent anion-selective channel protein 2	VDAC2	294	31620	62	77,20999718	39615111,89	64649818,58	0,612764471	0,15596884
233	A0A4W2FVY1	ATP synthase subunit f, mitochondrial (ATP synthase membrane subunit f)	ATP5MF	88	10297	8	48,85999858	1260985,369	1539138,032	0,819280235	0,082505553
234	A0A4W2FY09	ADP/ATP translocase (ADP,ATP carrier protein)	SLC25A4	298	32967	16	33,88999999	280889,4344	232807,2817	1,206531997	0,263093664
235	A0A4W2FY37	AU RNA binding methylglutaconyl-CoA hydratase	AUH	339	36204	5	17,11000055	847552,3051	1054243,173	0,80394384	0,297615156
236	A0A4W2FY99	Cilia and flagella associated protein 70	CFAP70	1124	126320	4	5,69400005	54475,35514	44689,39245	1,218977304	0,178188845
237	A0A4W2FYF3	Cilia and flagella associated protein 44	CFAP44	1852	213456	6	5,886000022	53719,06835	43895,97347	1,223781229	0,403417903
238	A0A4W2FZN9	C-type natriuretic peptide	NPPC	193	20294	7	23,31999987	112860,4394	17040,63765	6,623017383	6,0322E-05
239	A0A4W2G035	EF-hand domain-containing family member C2	EFHC2	747	87007	17	28,11000049	1467211,056	1511295,388	0,970830102	0,850739756
240	A0A4W2G038	Chromosome 4 C7orf31 homolog	C7orf31	583	67483	14	27,43999958	174186,3375	162625,3403	1,071089764	0,789772879
241	A0A4W2G0J9	Chromosome 8 C9orf135 homolog	CFAP95	218	25092	2	13,30000013	208254,7253	213487,7512	0,975487934	0,900919909
242	A0A4W2G0N7	Heat shock protein HSP 90-alpha	HSP90AA1	776	89100	5	8,246999979	444198,3556	340428,6562	1,304820694	0,323305515
243	A0A4W2G182	Tektin	TEKT3	490	56700	45	69,17999983	778050,5893	983948,8008	0,790742962	0,898824109
244	A0A4W2G186	Cytochrome c	LOC113891379	105	11704	4	26,66999996	100118,7086	83347,13597	1,201225543	0,282121058
245	A0A4W2G1A4	Keratin, type II cytoskeletal 2 oral-like	KRT76	597	62847	2	2,848000079	307437,4275	482636,1095	0,636996324	0,440968093
246	A0A4W2G1U8	Palmitoyl-protein thioesterase ABHD10, mitochondrial (EC 3.1.1.93) (EC 3.1.2.22) (A	ABHD10	330	36403	9	29,3900013	650641,5632	808474,6088	0,804776744	0,263531049
247	A0A4W2G4G1	2-iminobutanoate/2-iminopropanoate deaminase (EC 3.5.99.10) (Translation inhibit		137	14517	3	27,00999975	170990,736	191055,4047	0,894979842	0,836443921
248	A0A4W2G5C6	Dynein axonemal heavy chain 3	DNAH3	4069	466147	13	4,080000147	204806,848	212046,1411	0,965859822	0,888670417
249	A0A4W2G5W8	Dynein regulatory complex subunit 7	DRC7	807	95871	2	2,974000014	32560,77326	27069,02114	1,202879598	0,454341614
250	A0A4W2G637	Monocarboxylate transporter 1 (Solute carrier family 16 member 1)	SLC16A1 LOC113889450	501	54297	6	11,77999973	25872,04561	34111,51521	0,758454893	0,26655892
251	A0A4W2G685	Chromosome 10 C15orf65 homolog	PIERCE2	120	13771	1	13,33000064	17410,6107	19472,85745	0,894096347	0,956801148
252	A0A4W2G6F8	thioredoxin-disulfide reductase (EC 1.8.1.9)	TXNRD2	488	52514	3	8,196999878	26635,54196	29539,79683	0,901683316	0,674721891

253	A0A4W2G6V9	NADH dehydrogenase [ubiquinone] 1 beta subcomplex subunit 5, mitochondrial (Co	NDUFB5	189	21589	1	6,349000335	17485,84209	21561,921	0,81095938	0,589856937
254	A0A4W2G9X2	Cancer/testis antigen 47A-like	LOC113888212	281	30261	4	22,41999954	121552,0462	101962,477	1,192125278	0,297900975
255	A0A4W2GAI0	Cytochrome b-c1 complex subunit 2, mitochondrial	LOC113883210	453	48149	57	80,34999967	4504855,335	5148022,976	0,875065118	0,168739658
256	A0A4W2GC72	NADH dehydrogenase [ubiquinone] 1 beta subcomplex subunit 7 (Complex I-B18) (N/	NDUFB7	137	16398	2	16,06000066	121241,0504	97304,90274	1,245991178	0,191863076
257	A0A4W2GCD2	NADH dehydrogenase [ubiquinone] iron-sulfur protein 2, mitochondrial (Complex I-4	NDUFS2	510	57393	4	13,52999955	159045,3496	200983,2963	0,791336158	0,262315452
258	A0A4W2GCX1	Cilia- and flagella-associated protein 263	CCDC113	323	37773	3	12,69000024	74430,25527	72243,68972	1,030266527	0,939494502
259	A0A4W2GDN1	Acetyl-CoA acetyltransferase, mitochondrial (EC 2.3.1.9) (Acetoacetyl-CoA thiolase)	ACAT1	422	44889	19	49,52999949	867160,667	1080573,326	0,80250053	0,179719261
260	A0A4W2GDV6	NADH-cytochrome b5 reductase (EC 1.6.2.2)	CYB5R1	304	33723	17	54,28000093	4670182,507	2788764,828	1,674641928	0,038055603
261	A0A4W2GE75	Tubulin polymerization promoting protein family member 2	TPPP2	171	18564	9	51,45999789	282078,4989	171447,0357	1,6452807	0,178188845
262	A0A4W2GES6	aldehyde dehydrogenase (NAD(+)) (EC 1.2.1.3)	ALDH7A1	539	58596	2	5,750999972	63903,97309	68541,69072	0,932337274	0,661201855
263	A0A4W2GEV4	Glyceraldehyde-3-phosphate dehydrogenase (EC 1.2.1.12)	GAPDH	333	35868	18	28,83000076	6283773,256	3091325,997	2,03271129	0,290588235
264	A0A4W2GFX5	Milk fat globule-EGF factor 8 protein	MFGE8	572	63527	16	28,49999964	2193575,109	2165185,291	1,013111958	0,946553979
265	A0A4W2GG79	Fibronectin type-II domain-containing protein		151	17304	53	75,49999952	32909042,96	33966972,97	0,968854157	0,975750228
266	A0A4W2GGE1	Proteasome subunit alpha type	PSMA2	234	25899	4	19,6600005	116230,9547	132352,1413	0,878194743	0,749494315
267	A0A4W2GGZ0	NADH dehydrogenase [ubiquinone] flavoprotein 1, mitochondrial (EC 7.1.1.2)	NDUFV1	464	50652	15	47,8399992	759971,7715	783250,3842	0,970279475	0,833823915
268	A0A4W2GHS6	Proteasome 26S subunit, non-ATPase 2	PSMD2 LOC113891443	1688	188624	1	0,888600014	2939,833733	3441,455315	0,854241437	0,620534183
269	A0A4W2GIZ5	A-kinase anchoring protein 3	AKAP3	810	89355	134	73,21000099	1378227,721	681743,6108	2,021621764	0,610611446
270	A0A4W2GJ13	14-3-3 protein theta	YWHAQ	421	47052	2	9,025999904	16860,14815	14065,6917	1,198671811	0,850739756
271	A0A4W2GL58	Family with sequence similarity 24 member A		89	9388	2	20,21999955	2325182,459	2810552,959	0,827304268	0,579112446
272	A0A4W2GL67	Membrane cofactor protein		335	37318	3	4,775999859	36875,78768	36897,92375	0,999400073	0,748316576
273	A0A4W2GLB6	NADH dehydrogenase [ubiquinone] 1 alpha subcomplex subunit 5 (Complex I subuni	NDUFA5	116	13316	10	49,14000034	212544,7452	230048,0465	0,923914584	0,674721891
274	A0A4W2GMB7	Cilia and flagella associated protein 65	CFAP65	1740	196959	2	1,322000008	30539,98851	18279,29561	1,670742088	0,139171896
275	A0A4W2GMQ8	STI1 domain-containing protein		238	26971	1	5,882000178	25494,30269	19774,06733	1,289279655	0,539023906
276	A0A4W2GMV9	Cytochrome c oxidase subunit 7A-related protein, mitochondrial	COX7A2L LOC113901135	114	12544	2	40,34999907	37759,24137	49385,5779	0,764580328	0,257814642
277	A0A4W2GNK5	Transmembrane protein 256	TMEM256	113	11775	1	24,77999926	13611,28597	15036,53157	0,905214471	0,734231765
278	A0A4W2GPB3	SPEM family member 2	SPEM2	497	56428	1	2,414000034	15399,7546	18083,88999	0,851573119	0,539023906
279	A0A4W2GQJ6	Nucleoside diphosphate kinase homolog 7 (NDK 7)	NME7	377	42599	16	40,84999859	845195,7836	855848,1257	0,987553467	0,946553979
280	A0A4W2GQM1	Leucine rich repeat containing 37A	LRRC37A	2532	278113	72	22,83000052	5939993,075	5240993,903	1,133371491	0,456696907
281	A0A4W2GQY8	NADH dehydrogenase [ubiquinone] 1 alpha subcomplex subunit 2		92	10600	4	36,9599998	432203,696	587160,3	0,736091483	0,05146089
282	A0A4W2GRQ0	MICOS complex subunit	APOO	210	24002	5	24,2899999	173782,3046	136385,9407	1,274195154	0,35499493
283	A0A4W2GRR1	Fascin	FSCN3	498	56194	28	48,59000146	1563119,516	1931693,122	0,809196605	0,188928576
284	A0A4W2GSC1	Cytochrome c oxidase subunit 5B, mitochondrial (Cytochrome c oxidase polypeptid	LOC113894051	129	13834	12	56,59000278	1919622,228	2594381,512	0,739915166	0,178188845
285	A0A4W2GUI0	L-lactate dehydrogenase (EC 1.1.1.27)	LDHA	332	36598	29	68,37000251	10122928,5	11079011,62	0,913703211	0,901043935
286	A0A4W2GVQ9	Sperm associated antigen 16	SPAG16	628	70485	1	1,75199993	19807,70958	15895,88838	1,246090128	0,862711531
287	A0A4W2GW60	Histone H2A/H2B/H3 domain-containing protein		136	15678	2	15,44000059	49968,97008	55057,31586	0,907580933	0,665526058
288	A0A4W2GWM3	Dihydrolipoyllysine-residue succinyltransferase component of 2-oxoglutarate dehyd	DLST	529	56572	20	29,49000001	720849,9614	729322,5717	0,988382904	0,939494502
289	A0A4W2GWX1	Polyamine modulated factor 1 binding protein 1	PMFBP1	1006	117813	3	3,877000138	7047,257125	9673,358225	0,728522294	0,387351759
290	A0A4W2GWX2	Dynein regulatory complex protein 10 (IQ domain-containing protein D)	IQCD	444	51570	2	6,306000054	25316,9182	18912,0704	1,338664549	0,802448656
291	A0A4W2GXD6	Proteasome subunit alpha type	PSMA6	203	22842	2	11,81999967	8809,421289	12265,11794	0,718250027	0,116465155
292	A0A4W2GXF1	Glucose-6-phosphatase (EC 3.1.3.9)	G6PC3	346	38742	2	10,40000021	18274,49297	24328,46079	0,751156973	0,722964781
293	A0A4W2GXI2	Crystallin zeta	CRYZ	330	35382	3	11,81999967	151987,4542	140974,5131	1,078120087	0,722964781
294	A0A4W2GXN7	Sperm acrosome associated 4	SPACA4	126	12964	3	34,92000103	343718,8367	260236,4144	1,320794546	0,236159715
295	A0A4W2GYF8	Mitochondrial-processing peptidase subunit beta (EC 3.4.24.64) (Beta-MPP)	PMPCB	491	54266	2	4,481000081	64195,57645	69922,16561	0,918100518	0,799222135
296	A0A4W2GZC3	Propionyl-CoA carboxylase beta chain, mitochondrial (EC 6.4.1.3) (Propanoyl-CoA:ca	PCCB	544	58644	1	2,941000089	30054,66563	26501,8187	1,134060495	0,936563545
297	A0A4W2GZU2	Acetyl-CoA acyltransferase 2	ACAA2	645	68438	19	23,88000041	552965,4508	638356,0463	0,866233592	0,386344522
298	A0A4W2H0C9	Glutathione S-transferase LANCL1 (EC 2.5.1.18) (LanC-like protein 1)	LANCL1	399	45309	6	25,31000078	597416,2526	578807,1075	1,032150858	0,895740116
299	A0A4W2H0D3	Aldehyde dehydrogenase, mitochondrial (EC 1.2.1.3) (ALDH class 2) (ALDH-E2)	ALDH2	520	56653	19	52,49999762	1494052,847	1694799,727	0,881551267	0,373978461
300	A0A4W2H1Z3	Aldo-keto reductase family 1 member B1 (EC 1.1.1.21) (EC 1.1.1.300) (Aldose reducta	LOC113891189	303	34500	1	5,940999836	27863,90845	23506,07553	1,185391769	0,401399571
301	A0A4W2H1Z5	T-complex protein 1 subunit theta (CCT-theta)	CCT8	553	60203	6	13,74000013	280644,5302	180336,9485	1,556223129	0,126696309
302	A0A4W2H2D6	Proteasome subunit beta	PSMB4	264	29031	1	5,682000145	5735,308351	8172,672251	0,701766592	0,502994352
303	A0A4W2H537	Tektin	TEKT2	430	49886	36	61,15999818	7612655,523	8815708,714	0,863533015	0,179330148
304	A0A4W2H5E7	Cytochrome c oxidase subunit 7A2, mitochondrial (Cytochrome c oxidase subunit VI		83	9283	6	39,75999951	2757127,458	3287730,351	0,838611189	0,284967809
305	A0A4W2H5R5	NADH dehydrogenase [ubiquinone] 1 beta subcomplex subunit 9 (Complex I-B22) (N/	NDUFB9	179	21789	4	30,1699996	117988,7262	128158,3764	0,920647791	0,685582062
306	A0A4W2H658	Pyruvate dehydrogenase E1 component subunit beta (EC 1.2.4.1)	PDHB	359	39098	40	52,36999989	4528566,835	5298992,034	0,854609104	0,403417903
307	A0A4W2H6M4	Actin like 10	ACTL10	383	41133	3	19,32000071	85058,0274	62874,02495	1,352832548	0,26390167
308	A0A4W2H7K9	Ciliary microtubule inner protein 2C	FAM166C	201	23321	2	22,89000005	38560,26595	39571,45991	0,974446382	0,826989917
309	A0A4W2H888	Methionine adenosyltransferase 1A	DYDC1	175	20730	1	8,570999652	28540,16697	18731,70294	1,523629062	0,264398089
310	A0A4W2H8Q1	SAMM50 sorting and assembly machinery component	SAMM50	469	52042	8	15,56999981	263566,2754	288729,1392	0,912849587	0,554318913
311	A0A4W2H983	NAD(+) kinase (EC 2.7.1.23)	NADK2	555	61200	3	8,287999779	75949,654	82967,33031	0,91541639	0,798929132
312	A0A4W2HAB9	Spermatogenesis associated 19	SPATA19	154	17899	8	37,65999973	2266211,087	2549542,251	0,888869791	0,539023906
313	A0A4W2HBQ2	Alpha-L-fucosidase (EC 3.2.1.51)	FUCA1	444	51405	1	2,703000046	35887,92877	13506,73271	2,657039977	0,235916876
314	A0A4W2HCD2	Cilia and flagella associated protein 57	CFAP57	1279	148428	6	5,863999948	138237,467	115678,5454	1,195013877	0,257569081
315	A0A4W2HCZ3	Fibrous sheath interacting protein 2	FSIP2	6725	757955	128	22,86999971	160914,6095	154064,1702	1,044464844	0,995935621
316	A0A4W2HD14	Outer dense fiber of sperm tails 3B	ODF3B	395	42380	2	5,823000148	79489,38925	62909,61275	1,263549174	0,328778161

317	A0A4W2HD58	ATP-binding cassette sub-family A member 17-like	LOC113883545	1334	150599	2	2,098999918	14125,99896	10401,63673	1,358055403	0,303281271
318	A0A4W2HDC1	Calicin	CCIN	588	66890	64	69,9000001	45688064,98	38987517,04	1,171863931	0,126696309
319	A0A4W2HDX7	Cytochrome b-c1 complex subunit 10	UQCR11 LOC113895397	56	6520	2	21,43000066	43551,63166	38975,14935	1,11742052	0,898824109
320	A0A4W2HF26	Glycerol-3-phosphate dehydrogenase [NAD(+)] (EC 1.1.1.8)		351	38301	3	11,96999997	62892,3558	67236,13468	0,935395173	0,961830278
321	A0A4W2HFN3	Cullin-associated NEDD8-dissociated protein 1 (Cullin-associated and neddylation-di	CAND1	1223	135587	3	3,516000137	78900,78834	69093,66205	1,141939593	0,709112696
322	A0A4W2HG11	AFG3 like matrix AAA peptidase subunit 2	AFG3L2	805	89388	11	15,78000039	978813,7519	1143317,187	0,856117413	0,373978461
323	A0A4W2HG44	Histidine triad nucleotide binding protein 2	HINT2	165	17317	6	41,20999873	226987,6298	301480,2946	0,752910336	0,11028276
324	A0A4W2HGQ2	Sperm acrosome-associated protein 5	LOC113886686	156	17580	3	13,45999986	93668,75851	120235,7411	0,779042551	0,344056033
325	A0A4W2HH02	IZUMO family member 2	IZUMO2	221	25293	1	4,072000086	51257,40316	42343,38536	1,210517362	0,840385936
326	A0A4W2HH43	Electron transfer flavoprotein subunit alpha (Alpha-ETF)	ETFA	311	32704	16	68,80999804	14045,97485	13604,26246	1,032468676	0,855554095
327	A0A4W2HHB8	proton-translocating NAD(P)(+) transhydrogenase (EC 7.1.1.1)	NNT	1034	108491	8	10,44000015	516382,7608	554926,0892	0,930543312	0,759626475
328	A0A4W2HI57	Tektin	TEKT1	418	48719	16	42,34000146	523919,2488	621071,6701	0,84357293	0,149686248
329	A0A4W2HIR1	HtrA serine peptidase 2	HTRA2	361	38786	4	14,12999928	173485,1671	143484,099	1,209089845	0,379751635
330	A0A4W2HIT4	Dynein axonemal heavy chain 6	DNAH6	4154	475638	14	4,213000089	224609,0381	211711,5542	1,060920076	0,671062103
331	A0A4W2HIX9	Hydroxysteroid dehydrogenase-like protein 2	HSDL2	418	45135	10	29,8999995	1255056,663	1311787,665	0,956752908	0,727036926
332	A0A4W2HJ36	Heat shock protein family A (Hsp70) member 4 like	HSPA4L	1023	115519	5	6,745000184	72048,8313	58354,15898	1,234682027	0,373978461
333	A0A4W2HJ53	NADH dehydrogenase [ubiquinone] 1 alpha subcomplex subunit 13	GATAD2A	174	19597	2	10,92000008	470833,4012	632675,4836	0,744194162	0,04467807
334	A0A4W2HJC0	Testis expressed 51		168	18788	1	7,738000154	14997,68614	26030,89823	0,57614939	0,089733246
335	A0A4W2HK76	Carboxypeptidase Q (Plasma glutamate carboxypeptidase)	CPQ	484	53024	12	28,5100013	13479939,59	10180789,09	1,324056463	0,088790258
336	A0A4W2HKM7	NADH dehydrogenase [ubiquinone] 1 beta subcomplex subunit 4 (Complex I-B15) (N/	NDUFB4	129	15184	4	41,8599993	479030,0475	507196,1842	0,944466978	0,615908187
337	A0A4W2HKS8	Actin like 9	ACTL9	416	45540	23	62,98000216	1982731,462	1706049,808	1,162176774	0,297900975
338	A0A4W2HL85	Myeloid leukemia factor 1	MLF1	270	31227	7	14,06999975	117380,223	111699,2206	1,050859821	0,938963059
339	A0A4W2HLP1	FUN14 domain-containing protein 2 (Hepatitis C virus core-binding protein 6)	FUNDC2	190	20577	6	24,74000007	339883,8302	310148,9186	1,095873014	0,577235363
340	A0A4W2HLQ2	Tubulin beta chain	TUBB3	512	56029	104	41,80000126	136292,0292	87771,28592	1,552808846	0,35499493
341	A0A4W2HM97	L-serine ammonia-lyase (EC 4.3.1.17)	SRR	339	36681	4	21,52999938	37679,22379	36224,8536	1,040148408	0,861728279
342	A0A4W2HME8	Spermatogenesis associated 48	SPATA48	440	49710	3	9,77300033	76717,64143	77713,93794	0,987179951	0,889303593
343	A0A4W2HN15	Sorbitol dehydrogenase (SDH) (XDH) (EC 1.1.1.14) (EC 1.1.1.9) (L-iditol 2-dehydrogen	SORD	356	38127	3	15,72999954	125513,0928	95452,55807	1,314926444	0,126696309
344	A0A4W2HP47	Proteasome subunit alpha type	PSMA5	241	26411	4	22,40999937	219050,7411	245101,038	0,893716089	0,423145279
345	A0A4W2HPH7	ADP/ATP translocase (ADP,ATP carrier protein)	SLC25A6	410	44462	20	32,67999887	1485638,164	1472708,746	1,008779344	0,995935621
346	A0A4W2HPP0	Histone H2A		238	26496	8	39,07999992	393196,0823	351814,3726	1,117623704	0,857569767
347	A0A4W2HQP5	Nucleoredoxin (EC 1.8.1.8)	GLOD4	594	65077	1	4,039999843	21426,8257	21409,51219	1,000808683	0,838584395
348	A0A4W2HQW9	Stress-70 protein, mitochondrial (75 kDa glucose-regulated protein) (Heat shock 70 k	HSPA9	679	73772	19	25,04000068	1902875,741	1932105,749	0,984871425	0,938963059
349	A0A4W2HRF0	Citrate synthase	CS	466	51773	41	43,56000125	10253500,68	12863772,1	0,797083515	0,067458046
350	A0A4W2HRM3	Leucine-rich repeat and IQ domain-containing protein 4-like	LOC113888572	565	64870	1	2,477999963	22078,03418	9926,069546	2,22424738	0,116465155
351	A0A4W2HRY1	EF-hand calcium binding domain 5	EFCAB5	1505	172053	4	3,920000046	21423,88631	17938,83706	1,194273979	0,850739756
352	A0A4W2HS31	3-hydroxyisobutyrate dehydrogenase, mitochondrial (EC 1.1.1.31)	HIBADH	336	35410	20	55,36000133	1020268,083	1111943,161	0,917554169	0,640712455
353	A0A4W2HSU6	T-complex protein 1 subunit gamma	CCT3	507	56483	12	27,21999884	1197621,541	907325,6359	1,319946769	0,126671924
354	A0A4W2HSV6	Rab GDP dissociation inhibitor	GDI1	447	50566	2	7,605999708	94003,04006	76840,05198	1,223359923	0,331502239
355	A0A4W2HT18	Actin related protein T2	ACTRT2	377	41913	56	68,97000074	11745651,12	8894647,938	1,320530189	0,028478525
356	A0A4W2HTE9	H(+)-transporting two-sector ATPase (EC 7.1.2.2)	ATP6V1A	618	68475	2	7,119999826	46936,58813	54436,57416	0,862225238	0,820037469
357	A0A4W2HTI2	Histone H2B subacrosomal variant	LOC113879192	122	14238	20	73,76999855	27759567,57	32458185,01	0,855240906	0,352630178
358	A0A4W2HU94	Serpin family A member 5	SERPINA5 LOC113879927	374	41701	2	11,76000014	66029,40992	20970,7182	3,148648	0,00502487
359	A0A4W2HVH6	Outer dense fiber protein 2 (Cenexin) (Outer dense fiber of sperm tails protein 2)	ODF2	701	80750	294	79,46000099	827851,1027	843906,3935	0,980975034	0,841818162
360	A0A4W2HVJ6	Electron transfer flavoprotein subunit beta	ETFB	357	38331	6	23,81000072	605000,1329	728953,4255	0,829957185	0,315818638
361	A0A4W2HVK1	pyruvate carboxylase (EC 6.4.1.1)	PC	1266	139249	3	3,475999832	23997,44952	30836,73319	0,77820985	0,31849468
362	A0A4W2HVV4	Chromosome 5 C12orf10 homolog	MYG1	381	42967	7	26,51000023	296922,9071	331368,0773	0,896051634	0,856205159
363	A0A4W2HW44	Cytosol aminopeptidase (EC 3.4.11.1) (EC 3.4.11.5) (EC 3.4.13.23) (Cysteinylglycine-S	LAP3	519	56289	24	46,81999981	1339916,818	1517579,213	0,882930398	0,259862279
364	A0A4W2HWC1	Dihydrolipoamide acetyltransferase component of pyruvate dehydrogenase complex	PDHX	501	53886	13	22,75000066	582788,9861	716275,8225	0,813637663	0,269218621
365	A0A4W2HWK9	Isocitrate dehydrogenase [NAD] subunit, mitochondrial	IDH3B	385	42497	4	14,2900005	180499,5659	210227,7805	0,858590456	0,235916876
366	A0A4W2HWP1	Renin receptor (ATPase H(+)-transporting lysosomal accessory protein 2) (ATPase H(+)	ATP6AP2	377	42449	1	4,774999991	37251,42603	11775,45855	3,163479866	0,028478525
367	A0A4W2HWQ4	Coiled-coil domain containing 81	CCDC81	565	66189	3	4,602000117	82629,41574	76549,36173	1,07942658	0,644203165
368	A0A4W2HXB2	Seminal plasma protein PDC-109	LOC113876423	134	15580	25	77,60999799	10790054,74	8408831,352	1,283181252	0,900271027
369	A0A4W2HXI7	Protein SCO1 homolog, mitochondrial	SCO1	305	33717	2	12,1299997	33032,55697	32886,04866	1,004455029	0,915883223
370	A0A4W2HXJ5	Adenylate kinase isoenzyme 1 (AK 1) (EC 2.7.4.3) (EC 2.7.4.6) (ATP-AMP transphospho	AK1	211	23442	21	76,30000114	12740173,07	10558946,86	1,206576114	0,157649312
371	A0A4W2HXU7	Radial spoke head 3	RSPH3	606	68696	3	7,096000016	177814,6032	97809,34181	1,817971575	0,028478525
372	A0A4W2HY76	Chaperonin containing TCP1 subunit 6B	CCT6B	518	56605	8	24,8999998	17975,82266	15360,51909	1,170261405	0,35499493
373	A0A4W2HY90	DnaJ heat shock protein family (Hsp40) member C11	DNAJC11	559	63237	2	5,18799983	39259,57281	37140,29353	1,057061457	0,819248701
374	A0A4W2HYH2	protein deglycase (EC 3.5.1.124) (Maillard deglycase) (Parkinsonism-associated degly	PARK7	189	20035	10	64,55000043	509973,1111	432962,0477	1,17787024	0,291079228
375	A0A4W2HYH3	Dynein light chain Tctex-type 1	DYNLT1	113	12452	3	49,55999851	27473,73741	25817,15988	1,064165754	0,995935621
376	A0A4W2HYZ9	Mitochondrial carrier 2	MTCH2	303	33280	49	61,39000058	25061404,42	27656080,83	0,906180618	0,331502239
377	A0A4W2HZD8	Meiosis-specific nuclear structural protein 1	MNS1	526	64310	3	6,654000282	29825,42458	27279,17129	1,093340566	0,749494315
378	A0A4W2HZR1	Mitochondrial glutathione transporter SLC25A40 (Solute carrier family 25 member 4	LOC113891614	339	38277	2	7,964999974	117939,1177	111531,3738	1,057452389	0,640712455
379	A0A4W2HZT3	Testis expressed 49	TEX49	131	15447	1	9,923999757	67452,74174	64505,81819	1,045684616	0,850739756
380	A0A4W2I018	Solute carrier family 2, facilitated glucose transporter member 5 (Fructose transporte	SLC2A5	508	56009	1	1,377999969	590976,8467	642024,751	0,920489196	0,972332714

381	A0A4W2I0K8	Prohibitin	PHB2	299	33357	14	49,16000068	1014099,937	1351474,45	0,7503656	0,019992908
382	A0A4W2I1H5	Adenosine 5'-monophosphoramidase HINT1 (Desumoylating isopeptidase HINT1) (Hi	HINT1	126	13779	1	11,11000031	13578,34101	12452,51196	1,090409794	0,6998072
383	A0A4W2I1K4	Coiled-coil domain containing 63	CCDC63	558	65966	7	10,04000008	98731,50675	71157,47711	1,387507129	0,149930287
384	A0A4W2I1X5	Succinate dehydrogenase cytochrome b560 subunit, mitochondrial (Malate dehydro	SDHC	135	14561	1	9,629999846	226194,644	254485,8583	0,888829916	0,620534183
385	A0A4W2I292	Hexokinase-1 (EC 2.7.1.1) (Hexokinase type I)	HK1	921	102497	63	51,35999918	15935043,67	14478029,79	1,100636198	0,620530717
386	A0A4W2I2Q2	Heat shock 70 kDa protein 1-like	HSPA1L LOC113881288	641	70363	34	57,24999905	2400693,352	1872116,189	1,282342072	0,263093664
387	A0A4W2I344	Cytochrome b-c1 complex subunit 1, mitochondrial	UQCRC1	490	53827	37	54,07999754	6323132,714	8405465,656	0,752264416	0,061427518
388	A0A4W2I3C7	Hyaluronidase (EC 3.2.1.35)	LOC113891262	554	62449	46	48,9199996	58406,30334	40274,85328	1,450192827	0,826989917
389	A0A4W2I4L1	aspartyl aminopeptidase (EC 3.4.11.21)	DNPEP	433	47465	6	22,85999954	328790,66	300663,413	1,093550614	0,749494315
390	A0A4W2I5J8	ATP-dependent 6-phosphofructokinase (ATP-PFK) (Phosphofructokinase) (EC 2.7.1.1	PFKM	850	93427	5	8,471000195	226482,1972	170873,482	1,325437947	0,072184045
391	A0A4W2I5U3	Apolipoprotein A-II (Apolipoprotein A2)	APOA2	100	11202	2	43,99999976	5550,126472	24301,72228	0,228384079	0,001306308
392	A0A4W2I6G9	Sperm acrosome membrane-associated protein 3	SPACA3	163	18099	14	69,94000077	12182467,07	9128574,026	1,334542179	0,120157951
393	A0A4W2I6S5	Peptidase S1 domain-containing protein		350	38411	7	20,85999995	630988,1835	466853,4515	1,351576563	0,228656896
394	A0A4W2I8C2	Dynein axonemal intermediate chain 1	DNAI1	734	83124	4	7,902000099	102230,9577	92694,9229	1,102875481	0,566781527
395	A0A4W2I8F3	NADH dehydrogenase [ubiquinone] iron-sulfur protein 8, mitochondrial (Complex I-2	NDUFS8	195	22231	3	21,02999985	419606,2786	440951,4995	0,951592815	0,79484739
396	A0A4W2I8P6	Very long-chain specific acyl-CoA dehydrogenase, mitochondrial (EC 1.3.8.9)	ACADVL	677	72731	39	54,65000272	4366051,735	5046539,719	0,86515751	0,261388431
397	A0A4W2I8R7	Dihydroxyacetone phosphate acyltransferase (DAP-AT) (DHAP-AT) (EC 2.3.1.42)	GNPAT	680	77682	11	17,35000014	154661,103	165348,8138	0,93536264	0,728739825
398	A0A4W2I8V6	Mitochondrial chaperone BCS1 (BCS1-like protein)	BCS1L LOC113875138	419	47504	4	14,80000019	174475,5762	196785,391	0,886628704	0,373978461
399	A0A4W2I9C4	Enoyl-CoA hydratase, mitochondrial (EC 4.2.1.17) (EC 5.3.3.8) (Enoyl-CoA hydratase 1	ECHS1	290	31178	5	30,00000119	104020,8981	113638,4006	0,915367495	0,655023404
400	A0A4W2I9M3	Rae1 protein homolog (mRNA-associated protein mrnp 41)	RAE1	368	40981	1	4,075999931	19175,33696	13084,78368	1,465468396	0,178217033
401	A0A4W2I9W7	Ras-related protein Rab-2A (EC 3.6.5.2)	RAB2A	212	23519	47	84,90999937	7996836,305	6745322,549	1,185538015	0,127805396
402	A0A4W2IB22	Leucine rich repeat containing 74A	LRRC74A	486	54176	2	6,995999813	195835,7396	122695,0318	1,596117925	0,007885062
403	A0A4W2IB54	Fructose-bisphosphate aldolase (EC 4.1.2.13)	ALDOA	445	48167	79	79,78000045	4245902,033	4222438,506	1,005556867	0,900890629
404	A0A4W2ICU0	Four and a half LIM domains 1	FHL1	309	35024	6	21,68000042	37024,61678	45644,03035	0,811160112	0,256865367
405	A0A4W2ICW9	Profilin	PFN3	137	14629	14	81,0199976	3124695,232	2355983,388	1,326280673	0,137151486
406	A0A4W2IDE6	UBC core domain-containing protein		147	16673	1	16,32999927	28231,94233	22300,59268	1,265972736	0,920965712
407	A0A4W2IEM6	Dynein light chain	DNAL4	134	14634	2	13,42999935	289462,4284	188564,3375	1,535085755	0,824808492
408	A0A4W2IEV4	Transmembrane protein 14C	TMEM14C	114	11721	2	35,08999944	98121,1675	115389,7656	0,850345497	0,291079228
409	A0A4W2IFA5	Succinate dehydrogenase [ubiquinone] iron-sulfur subunit, mitochondrial (EC 1.3.5.	SDHB	285	31993	8	32,98000097	2130042,224	2651506,419	0,803332856	0,546607241
410	A0A4W2II32	Tudor domain-containing protein 3	TDRD3	736	82237	1	0,951099955	2561612,165	1800142,933	1,423004873	0,041235348
411	A0A4W2II81	Transmembrane protein 65	TMEM65	236	25041	1	4,661000147	156948,492	182868,8743	0,858257003	0,658082286
412	A0A4W2IIC9	T-complex-associated-testis-expressed 1	TCTE1	502	55376	1	3,984000161	28359,81247	20524,24508	1,381771284	0,351036391
413	A0A4W2IJI3	Disintegrin and metalloproteinase domain-containing protein 20-like	LOC113897899	723	82091	22	24,07000065	408524,1589	574582,9944	0,710992429	0,134305811
414	A0A4W2IK05	Coiled-coil domain containing 114	CCDC114	797	89300	3	4,893000051	50833,3169	40786,80388	1,246317732	0,383567814
415	A0A4W2IKF1	Glutamine synthetase (EC 6.3.1.2)	GLUL	373	42031	26	51,74000263	5018009,657	3441332,171	1,458159052	0,038463698
416	A0A4W2IKL9	D-beta-hydroxybutyrate dehydrogenase, mitochondrial (EC 1.1.1.30) (3-hydroxybut	BDH1	344	38391	7	24,70999956	634555,43	655192,3111	0,968502559	0,89270578
417	A0A4W2IKP9	tRNA-dihydrouridine synthase (EC 1.3.1.-)	BCAP29	369	42809	2	7,045999914	29051,59394	38667,66304	0,751314966	0,307832535
418	A0A4W2IL98	Synaptophysin like 1	SYPL1	297	32290	3	14,48000073	519131,8121	524267,1807	0,990204673	0,972332714
419	A0A4W2ILV6	Pyruvate kinase (EC 2.7.1.40)	PKM	655	71874	42	45,64999938	30657,78758	24720,10185	1,240196653	0,414269402
420	A0A4W2IMA0	Succinate--CoA ligase [GDP-forming] subunit beta, mitochondrial (EC 6.2.1.4) (GTP-s	SUCLG2	432	46663	39	64,34999704	467542,99	579255,9279	0,807144075	0,544675396
421	A0A4W2IMG1	Keratin associated protein 6-2	KRTAP6-2	69	7021	1	20,29000074	778801,3381	5958,427593	130,7058491	0,598003408
422	A0A4W2IPA3	BPI fold-containing family A member 1 (Palate lung and nasal epithelium clone prote	BPIFA1	306	32258	1	4,90199998	53455,59134	5286,908846	10,11093493	0,000483363
423	A0A4W2IQG1	Keratin 24	KRT24	525	55115	5	6,285999715	88232,64958	145779,1413	0,605248795	0,447720839
424	A0A4W2IQJ0	Family with sequence similarity 81 member B	FAM81B	445	51475	5	17,98000038	97342,30567	68498,24454	1,421091976	0,323305515
425	A0A4W2IR05	Outer dynein arm-docking complex subunit 4 (Tetratricopeptide repeat protein 25)	ODAD4	683	78354	10	19,17999983	260791,5285	158598,6753	1,644348719	0,2263211
426	A0A4W2IRM1	Cilia- and flagella-associated protein 206	CFAP206	582	66645	3	4,123999923	17901,78699	17267,60014	1,036726983	0,826989917
427	A0A4W2IRR0	Putative malate dehydrogenase 1B	MDH1B	526	59163	1	2,470999956	19726,21448	13217,83529	1,492393727	0,191863076
428	A0A4W2ITX2	NADH dehydrogenase [ubiquinone] 1 beta subcomplex subunit 3 (Complex I-B12) (N	NDUFB3	179	20284	1	6,703999639	135873,43	177809,6225	0,764151164	0,137302261
429	A0JN77	Triokinase/FMN cyclase (EC 2.7.1.28) (EC 2.7.1.29) (EC 4.6.1.15) (Bifunctional ATP-de	TKFC DAK	578	59160	1	3,632999957	24371,0217	21995,55948	1,107997354	0,749494315
430	A1A4J9	DnaJ (Hsp40) homolog, subfamily A, member 3 (DnaJ heat shock protein family (Hsp4	DNAJA3	453	49254	6	17,44000018	212789,6841	210007,9726	1,013245743	0,857569767
431	A1A4K9	Succinyl-CoA:3-ketoacid-coenzyme A transferase (EC 2.8.3.5)	OXCT2	517	56009	3	10,24999991	29987,01381	32040,29245	0,935915734	0,993202671
432	A1A4P8	LOC784495 protein	LOC784495	168	19359	9	49,39999878	525265,3814	559234,2722	0,93925821	0,709112696
433	A2VDL2	Solute carrier family 2, facilitated glucose transporter member 3 (GLUT-3) (Glucose tr	SLC2A3	494	54119	15	15,98999947	4728104,915	4242968,601	1,114338889	0,820037469
434	A2VDZ0	Serine/threonine-protein phosphatase 2A 56 kDa regulatory subunit	PPP2R5A	489	56526	1	2,65800003	22385,96929	15217,02115	1,471113767	0,157485667
435	A4FV36	Spermatogenesis-associated protein 20	SPATA20	789	88360	5	9,379000217	153911,3901	142326,4613	1,081396873	0,615908187
436	A4IFP2	KRT4 protein (Keratin 4)	KRT4	549	58046	12	22,58999944	178860,179	104745,0509	1,707576419	0,478411675
437	A5D7S5	SLC25A35 protein	SLC25A35	311	33688	2	11,2499997	55971,08346	59726,59315	0,937121649	0,875767994
438	A5D954	Mitochondrial 2-oxoglutarate/malate carrier protein (Solute carrier family 25 memb	SLC25A11	311	33899	3	12,21999973	215767,7602	228767,4905	0,943174923	0,995935621
439	A5D973	Alpha isoform of regulatory subunit A, protein phosphatase 2	PPP2R1A	589	65282	1	3,395999968	21967,20039	27989,89467	0,784826118	0,280418181
440	A5D9E7	Trifunctional enzyme subunit beta, mitochondrial (EC 2.3.1.155) (EC 2.3.1.16) (TP-be	HADHB	496	53704	17	39,52000141	2671925,181	3070226,422	0,87026975	0,481330957
441	A5D9G3	Succinate-CoA ligase subunit beta (EC 6.2.1.-)	LOC283398	432	46664	38	64,1200006	66830,1713	82462,36248	0,810432412	0,935160425
442	A5PJ81	LACTB protein (Lactamase beta)	LACTB	556	61653	3	6,835000217	88320,21191	85558,99153	1,032272708	0,890904408
443	A5PJA6	Stomatin (EPB72)-like 2 (Stomatin like 2)	STOML2	356	38733	2	12,08000034	23409,26532	24677,74666	0,94859817	0,722964781
444	A5PJD4	KLKBL4 protein	KLKBL4	327	36921	3	8,257000148	339499,1329	255067,1452	1,331018672	0,157649312

445	A5PK71	Parkin coregulated gene protein	PACRG	257	29284	15	56,80999756	2373998,139	1700444,385	1,396104547	0,017424933
446	A5PKG4	cysteine desulfurase (EC 2.8.1.7)	NFS1	457	50344	3	7,876999676	33439,34828	38390,25059	0,87103751	0,51475685
447	A5PKH9	POC1 centriolar protein homolog B	WDR51B	478	53723	5	7,321999967	43213,60695	46453,24434	0,930260256	0,829647132
448	A6H746	C12H13ORF26 protein (Testis expressed 26)	TEX26 C12H13ORF26	290	33737	1	3,448000178	116762,3108	94551,25814	1,234910176	0,21776382
449	A6H758	C11H9ORF9 protein	C11H9ORF9	223	25006	23	61,87999845	3855352,169	2560487,853	1,505710002	0,04467807
450	A6QLB8	[tau protein] kinase (EC 2.7.11.26)	GSK3A	495	52263	6	15,55999964	55555,0131	28530,67114	1,947203163	0,079715878
451	A6QLG3	Protein-serine/threonine kinase (EC 2.7.11.-)	PDK3	415	47913	1	3,613999858	128283,659	137540,8201	0,932695173	0,89270578
452	A6QLL2	MTX2 protein	MTX2	264	29679	3	14,76999968	162163,2007	162375,3339	0,998693563	0,956801148
453	A6QNM9	SLC25A12 protein (Solute carrier family 25 member 12)	SLC25A12	675	74530	8	15,55999964	227289,6013	199300,162	1,140438618	0,647930368
454	A6QNX2	DPP7 protein	DPP7	488	53642	6	19,67000067	620860,3944	414256,1165	1,498735612	0,047101558
455	A6QNZ7	Keratin, type I cytoskeletal 10 (Cytokeratin-10) (Keratin-10)	KRT10	526	54849	13	17,87	448431,9829	515113,303	0,870550188	0,444776105
456	A6QPG6	LANCL2 protein	LANCL2	433	49166	6	18,70999932	236112,5839	163240,1166	1,446412737	0,039053585
457	A6QQ54	Solute carrier family 25 member 3 (Phosphate carrier protein, mitochondrial) (Phosp	LOC616319	330	36606	13	33,93999934	2349945,529	2719138,647	0,86422424	0,327210741
458	A6QQK5	MMAA protein	MMAA	421	46687	1	4,038000107	27391,62169	26354,60914	1,039348432	0,912555075
459	A7E3P5	15-oxoprostaglandin 13-reductase (EC 1.3.1.48) (15-oxoprostaglandin 13-reductase)	ZADH1	296	32091	2	8,107999712	59551,74466	46812,5145	1,272133003	0,539023906
460	A7E3Q2	Heat shock-related 70 kDa protein 2	HSPA2	636	69809	22	34,74999964	1530826,101	1009602,095	1,516266764	0,012113394
461	A7MB90	non-specific serine/threonine protein kinase (EC 2.7.11.1)	CSNK1G2	414	47268	3	10,14000028	104507,4876	86100,42192	1,21378601	0,403417903
462	A7MBF3	STYXL1 protein	STYXL1	309	35367	1	4,207000136	30283,84234	27901,98969	1,085364975	0,829647132
463	A7Z057	14-3-3 protein gamma	YWHAG	247	28303	2	5,668000132	37647,52299	23394,86252	1,60922181	0,597256593
464	B2KJ42	Fructose-1,6-bisphosphatase 1 (EC 3.1.3.11) (D-fructose-1,6-bisphosphate 1-phosph		338	36740	13	34,0200007	225214,6056	192851,7308	1,167812208	0,391863779
465	B5B3R8	Alpha-S1-casein	CSN1S1	214	24443	1	5,606999993	9845,358043	14240,21199	0,69137721	0,35499493
466	Q2YDM5	Sperm microtubule inner protein 8 (Testis, prostate and placenta-expressed protein)	SPMIP8 TEPP	216	24905	14	80,08999825	1814782,898	2034880,149	0,891837732	0,473409662
467	D5K1R5	Sperm protamine P1	PRM1	51	6713	1	17,64999926	51417,11715	118952,1549	0,432250405	0,081462806
468	E1B6Z7	Metabolism of cobalamin associated B	MMAB	233	25743	4	23,6499995	169641,1666	184046,3624	0,921730614	0,748316576
469	E1B7Q2	Adenylate kinase 7	AK7	724	83016	4	7,458999753	173571,6962	102535,0294	1,692803886	0,006770554
470	E1B7S8	Acrosin-binding protein (Acrosin-binding protein, 60 kDa form) (Proacrosin-binding	ACRBP	542	61273	118	59,03999805	63645068,22	41392133,66	1,537612647	0,001306308
471	E1B7X2	Uncharacterized protein				112	79,24000025	922546,8625	748467,666	1,232580784	0,267201444
472	E1B836	Enkurin	ENKUR	259	30157	8	35,51999927	129084,2244	122890,8971	1,050396957	0,749494315
473	E1B844	Mitochondrial import inner membrane translocase subunit TIM44	TIMM44	459	52004	2	5,663999915	22729,65537	27232,77686	0,834643323	0,915883223
474	E1B8N5	Sodium/potassium-transporting ATPase subunit alpha	ATP1A4	975	107456	27	33,98000002	1128573,862	1281805,531	0,880456383	0,330497504
475	E1B8R6	RBR-type E3 ubiquitin transferase (EC 2.3.2.31)	ARIH2	488	56785	3	7,942999899	56434,42008	59896,69841	0,94219584	0,92558903
476	E1B8W3	Outer dynein arm-docking complex subunit 2 (Armadillo repeat-containing protein 4	ODAD2 ARMC4	1044	115859	12	17,43000001	227289,0719	202992,5869	1,119691489	0,43754635
477	E1B958	ALMS1 centrosome and basal body associated protein	ALMS1	4331	482210	5	1,408000011	150298,8267	184541,8224	0,81444317	0,258073581
478	E1B993	Ankyrin repeat and EF-hand domain containing 1	ANKEF1	776	86915	1	2,31999997	34565,87459	28306,46026	1,221130239	0,649061468
479	E1B9F6	Elongation factor 1-alpha				3	7,500000298	290321,3366	210940,5921	1,376318013	0,149930287
480	E1B9R5	Dynein axonemal heavy chain 8	DNAH8	4493	515264	74	18,02999973	2124252,023	1695395,326	1,252953803	0,03202207
481	E1B9Y3	Lipocalin 12	LCN12	193	21636	2	19,68999952	44758,65797	57736,48225	0,775223156	0,686853185
482	E1BA21	Transcription factor A, mitochondrial	TFAM	246	28830	7	32,51999915	1203418,35	1043370,88	1,15339461	0,696102603
483	E1BA36	Sperm associated antigen 17	SPAG17	2229	252206	1	0,493500009	67149,99381	41308,14369	1,625587301	0,059298148
484	E1BAJ3	Coiled-coil domain containing 40	CCDC40	1125	129259	4	5,067000166	147605,144	111692,5373	1,321530942	0,331502239
485	E1BBC4	Dynamin-like GTPase OPA1, mitochondrial (EC 3.6.5.5)	OPA1	1000	115850	2	2,562000044	38640,52091	40176,546	0,961768115	0,632942695
486	E1BC58	RAB2B, member RAS onco family	RAB2B	216	24167	36	79,17000055	3693439,265	3297767,43	1,119981728	0,453022686
487	E1BCC9	Small nuclear ribonucleoprotein Sm D1 (snRNP core protein D1)	SNRPD1	109	12145	1	14,92999941	11282,4549	11122,81156	1,014352787	0,850739756
488	E1BCN8	Phosphoethanolamine/phosphocholine phosphatase 1	PHOSPHO1	348	38318	5	28,83999944	223381,0925	276859,5099	0,806839153	0,175687361
489	E1BCW3	ATP-dependent 6-phosphofructokinase (ATP-PFK) (Phosphofructokinase) (EC 2.7.1.1	PFKP	909	97498	10	15,20999968	82644,66996	62436,18155	1,323666309	0,391863779
490	E1BD64	Mitochondrial pyruvate carrier	MPC2	127	14282	1	9,448999912	77876,68643	99707,86583	0,781048574	0,3242436
491	E1BD83	Proteasome subunit alpha type	PSMA8	278	30903	6	33,59999955	472629,3886	597713,2723	0,790729285	0,149930287
492	E1BDA8	Izumo sperm-oocyte fusion 1	IZUMO1	315	35693	12	22,98000008	2008051,29	2066232,81	0,971841741	0,758217953
493	E1BDM7	Zinc finger MYND-type containing 12	ZMYND12	338	38691	3	10,10999978	167335,5646	146434,2854	1,142734873	0,280910069
494	E1BDQ6	NME/NM23 family member 5	NME5	211	24002	8	58,37000012	81670,68312	70714,03395	1,154943065	0,566781527
495	E1BE64	Testis specific 10	TSGA10	892	103566	87	54,47999835	4253625,817	5134823,783	0,828387886	0,149930287
496	E1BED9	Glutathione S-transferase omega (GSTO) (EC 1.20.4.2) (EC 1.8.5.1) (EC 2.5.1.18) (Glut	GSTO2	244	28261	29	56,15000129	11909304,33	16133984,04	0,738150248	0,267201444
497	E1BEL9	Coenzyme A synthase	COASY	562	61800	1	2,847000025	44423,96765	42695,66897	1,040479485	0,89270578
498	E1BFM2	Peptidase S1 domain-containing protein		497	54750	3	9,809000045	158170,1277	91715,06888	1,724581681	0,046739942
499	E1BGD1	Translocase of outer mitochondrial membrane 34	TOMM34	309	34743	2	8,414000273	37948,81456	42365,79648	0,895741794	0,872033851
500	E1BHQ2	Selenoprotein V	SELENOV	381	40621	1	8,399000019	11556,7427	6689,177794	1,72767761	0,210140429
501	E1BHV9	Ornithine decarboxylase antizyme 3	OAZ3	235	27509	8	48,71999919	3418628,171	3916326,667	0,872917012	0,660680191
502	E1BHW8	UBX domain protein 11	UBXN11	475	53409	2	6,526000053	71143,74341	52605,07588	1,352412143	0,416170442
503	E1BI52	Serine protease 42	PRSS42	337	36709	2	9,792000055	33464,69582	36601,47257	0,914299165	0,694405834
504	E1BIN5	Cullin 3	CUL3	784	90773	3	6,379999965	29599,29359	26426,71041	1,120052142	0,972332714
505	E1BJB1	Tubulin beta chain	TUBB2A	445	49907	153	74,83000159	19954,73709	20911,64293	0,954240523	0,939494502
506	E1BJG2	Radial spoke head 6 homolog A	RSPH6A	703	79699	19	22,86999971	1564136,88	1167846,581	1,339334212	0,05146089
507	E1BJG7	Chromosome 15 C11orf42 homolog	C15H11orf42	333	36540	2	7,507999986	4709,728967	3257,330524	1,445886112	0,624310673
508	E1BJL9	Cilia- and flagella- associated protein 210	CFAP210 CCDC173	547	65300	4	6,398999691	42242,35082	32918,51787	1,283239755	0,26655892

509	E1BJW4	Cilia and flagella associated protein 69	CFAP69	855	96423	1	1,487999968	28482,48632	44738,81309	0,636639293	0,912478478
510	E1BJX2	Glutathione transferase		363	40919	3	15,35000056	21248,79029	23105,31369	0,919649504	0,7288098
511	E1BKF9	Cilia- and flagella-associated protein 52	CFAP52	623	68637	6	13,79999965	300781,8799	338053,1217	0,889747382	0,668791875
512	E1BLB4	Dynein axonemal heavy chain 17	DNAH17	4462	509241	81	21,64999992	10944,71081	9246,995404	1,183596436	0,745351842
513	E1BLI3	Ubiquitin-like protease family profile domain-containing protein	LOC782385	212	24156	3	13,12000006	6161,328574	4061,585688	1,516976139	0,336384557
514	E1BM93	Retinol dehydrogenase 11	RDH11	340	37807	9	31,76000118	309884,9148	329456,5132	0,940594289	0,664034446
515	E1BMD1	Cilia- and flagella-associated protein 43	CFAP43	1668	192834	6	5,705999956	106191,8639	92489,22686	1,148153871	0,50928674
516	E1BMQ6	Calcium-transporting ATPase (EC 7.2.2.10)	ATP2A3	999	109321	1	1,501999982	32399,11624	17195,1917	1,884196281	0,179869978
517	E1BN01	Axonemal dynein light intermediate polypeptide 1 (Inner dynein arm light chain, axo	DNALI1	265	30323	17	56,98000193	1419562,697	1062995,224	1,33543657	0,05146089
518	E1BNA2	X-prolyl aminopeptidase 3	XPNPEP3	491	55561	6	19,72000003	212927,5871	246619,2484	0,863385922	0,417979925
519	E1BNL3	Zonadhesin	ZAN	2437	266609	98	32,85999894	8140095,048	7952027,818	1,023650223	0,995935621
520	E1BNS6	Sperm-associated antigen 8	SPAG8	484	51542	7	27,68999934	34262,85569	30512,63822	1,122907021	0,689937702
521	E1BNS9	L-lactate dehydrogenase (EC 1.1.1.27)	LDHC	332	36015	63	87,94999719	20599437,62	23432973,21	0,879079126	0,439676506
522	E1BP40	Cilia and flagella associated protein 61	CFAP61	1235	141145	5	5,259000137	16372,21056	21000,929	0,779594587	0,980566238
523	E1BPM9	Dynein axonemal intermediate chain 2	DNAI2	607	69284	5	10,23000032	630954,5055	510022,6502	1,237110754	0,103202567
524	E1BPT0	Leucine rich repeats and death domain containing 1	LRRD1	863	98513	4	6,488999724	20301,64561	18048,00308	1,124869356	0,748316576
525	E1BPV0	Armadillo repeat containing 3	ARMC3	874	96145	6	11,37000024	264774,8105	203932,9272	1,298342618	0,081462806
526	E1BPY2	Equatorin	EQTN	323	36543	2	14,2900005	20773,14118	28137,72867	0,738266455	0,280418181
527	F1MAU4	Prolylcarboxypeptidase	PRCP	484	54989	5	15,02999961	848426,9418	648115,9569	1,309066584	0,126671924
528	F1MB31	deleted				2	7,260999829	35473,80853	24772,08227	1,432007537	0,195882065
529	F1MBE1	Threonine synthase like 1	THNSL1	724	80975	2	3,742999956	14661,05016	15774,72093	0,929401555	0,694405834
530	F1MBL6	non-specific serine/threonine protein kinase (EC 2.7.11.1)	SMG1	3646	409116	1	0,191400002	2044404,883	1818995,688	1,123919587	0,500318666
531	F1MBQ1	Serine/threonine-protein phosphatase with EF-hands (EC 3.1.3.16)	PPEF1	658	75965	9	17,77999997	579731,9881	454796,7005	1,274705792	0,073531958
532	F1MBS4	Solute carrier family 25 member 10	SLC25A10	294	32232	1	3,832999989	27546,1606	36907,04002	0,746366021	0,290588235
533	F1MBU8	Dpy-19 like 2	DPY19L2	773	89528	29	24,32000041	4244695,565	5968376,733	0,71119766	0,038463698
534	F1MBV2	Ribose-phosphate pyrophosphokinase 1 (EC 2.7.6.1) (Phosphoribosyl pyrophosphate	PRPS1L1	329	35658	2	7,598999888	124243,392	79519,94734	1,562417935	0,26390167
535	F1MBZ1	Kelch like family member 10	KLHL10	608	68916	2	5,099000037	64017,2785	52334,95269	1,223222248	0,577235363
536	F1MC11	Keratin 14	KRT14	515	55895	10	21,35999948	39738,80503	26122,42338	1,521252621	0,961830278
537	F1MDK8	ADP/ATP translocase (ADP,ATP carrier protein)	SLC25A31	323	35654	43	83,27999711	1516963,841	1299389,723	1,167443312	0,993202671
538	Q75WB5	5-oxoprolinase (EC 3.5.2.9) (5-oxo-L-prolinase) (5-OPase) (Pyroglutamase)	OPLAH	1288	137354	5	5,702000111	51245,95672	44103,57306	1,161945692	0,525759353
539	F1MEI0	Fibrous sheath CABYR binding protein	FSCB	791	84536	12	20,5400005	52265,15749	40631,65814	1,286316136	0,457406445
540	F1MEM9	Tetratricopeptide repeat domain 19	TTC19	382	42476	3	12,30999976	111025,2932	125480,9797	0,884797788	0,373978461
541	F1MES6	UBX domain protein 6	UBXN6	441	49806	3	19,94999945	35193,93948	24949,68878	1,410596332	0,26390167
542	F1MEX9	long-chain-fatty-acid--CoA ligase (EC 6.2.1.3)	ACSL3	720	80282	2	4,166999832	51378,02866	47338,54387	1,085331834	0,963426705
543	F1MEY2	Enoyl-[acyl-carrier-protein] reductase, mitochondrial (EC 1.3.1.104) (2-enoyl thioest	MECR	419	45172	5	18,55999976	39944,5243	26699,8336	1,496058923	0,125398808
544	F1MFA3	Pitrilysin metalloproteinase 1	PITRM1	1032	116381	8	8,624000102	390370,4284	486868,1687	0,801799036	0,627287151
545	F1MFB3	Uncharacterized protein	LOC132342087	588	68130	3	5,951999873	326761,8327	217823,8883	1,500119364	0,038709139
546	F1MFR7	Solute carrier family 9 member B1	SLC9B1	513	55287	3	7,992000133	9278,624692	9960,528835	0,931539364	0,995935621
547	F1MGC0	Succinate--CoA ligase [ADP-forming] subunit beta, mitochondrial (EC 6.2.1.5) (ATP-sp	SUCLA2	463	50146	6	22,89000005	471648,8938	435511,5517	1,08297677	0,824808492
548	F1MGQ1	Deoxyribonuclease	DNASE1L3	305	34936	13	43,27999949	437850,5473	98503,38033	4,445030677	0,00013993
549	F1MGY9	Aspartylglucosaminidase	AGA	346	36965	20	44,22000051	945581,0437	596479,2206	1,58527072	0,021763449
550	F1MH21	Membrane metalloendopeptidase like 1	MMEL1	779	88991	2	3,593999892	176282,0782	125391,4556	1,40585399	0,116465155
551	F1MHK9	ATP binding cassette subfamily A member 14	ABCA14	1655	188235	10	6,401000172	307096,7701	332749,864	0,922905772	0,696102603
552	F1MI43	Sperm surface protein Sp17 (Sperm autoantigenic protein 17)	SPA17	147	16960	8	44,22000051	248392,0735	230821,8539	1,07612026	0,715927018
553	F1MIC3	Glycogen synthase kinase-3 beta (EC 2.7.11.26)	GSK3B	410	45710	4	16,06000066	29307,39842	21859,78681	1,340699188	0,104219828
554	F1MIH2	Septin	SEPTIN7	417	48669	7	18,70999932	69163,17431	64437,13192	1,073343463	0,915883223
555	F1MIH9	Phospholipase B-like (EC 3.1.1.-)	PLBD2	589	65723	3	6,791000068	354281,6089	393015,5064	0,901444353	0,664034446
556	F1MIM1	Uncharacterized protein		111	13227	1	10,62000021	175666,7164	122455,6625	1,434533225	0,218520607
557	F1MJ55	Casein kinase II subunit alpha (CK II alpha) (EC 2.7.11.1)	CSNK2A2	350	41171	6	27,14000046	190299,4138	138442,2092	1,374576546	0,249343849
558	F1MJ59	Omega-amidase NIT2 (EC 3.5.1.3) (Nitrilase homolog 2)	NIT2	283	31159	1	3,886999935	49240,58841	50522,55066	0,97462594	0,939494502
559	F1MJQ7	Fibronectin type III domain containing 8	FNDC8	321	35343	41	61,05999947	948065,8439	658196,7809	1,440398786	0,038709139
560	F1MJS8	A-kinase anchoring protein 3	AKAP3	858	94740	154	77,03999877	19082951,23	15310808,35	1,246371243	0,077490335
561	F1MJY8	Immunity related GTPase cinema (Interferon-gamma-inducible GTPase IFGGE proteir	IRGC IFGGE	465	50697	9	33,75999928	1833188,853	1487451,571	1,232435992	0,373978461
562	F1MK41	Short chain dehydrogenase/reductase family 39U member 1	SDR39U1	326	34631	3	19,3900004	45540,35813	44324,63565	1,027427693	0,781648136
563	F1MK55	Dynein axonemal heavy chain 2	DNAH2	4424	508289	14	4,566000029	415936,2492	324123,973	1,283262837	0,016045645
564	F1MKF8	Sulfide quinone oxidoreductase	SQOR	450	49856	20	38,67000043	968416,2091	1079649,51	0,896972768	0,625168767
565	F1MLB8	ATP synthase subunit alpha	ATP5F1A	553	59690	92	68,72000098	200176,9133	177687,3431	1,126568217	0,995935621
566	F1MLM6	ITPRIP like 1				2	6,763999909	46542,13561	41397,62966	1,124270544	0,723696372
567	F1MLS7	Membrane spanning 4-domains A13	MS4A13	176	19737	1	6,25	47134,53184	25553,58468	1,844536977	0,065711753
568	F1MM32	Sulfhydryl oxidase (EC 1.8.3.2)	QSOX1	608	67021	7	16,60999954	172029,4991	100316,6185	1,71486541	0,003482382
569	F1MMV1	EF-hand domain-containing family member B (Cilia- and flagella-associated protein 2	EFHB CFAP21	877	97819	10	19,49999928	207398,6867	273288,2218	0,758900934	0,038463698
570	F1MMZ2	Ferritin	FTMT	242	27396	12	52,89000273	473297,4433	620414,3891	0,762873092	0,096113278
571	F1MN23	Dehydrogenase/reductase SDR family member 7B (Short-chain dehydrogenase/reduc	DHRS7B	325	35104	2	9,538000077	24023,95631	23056,59942	1,041955749	0,89270578
572	F1MNH2	Radial spoke head component 1	RSPH1	308	34964	7	29,21999991	137376,201	113179,7812	1,213787477	0,044354441

573	F1MNL6	Nucleoporin 210 like	NUP210L	1883	209539	8	5,466999859	264543,342	380728,3039	0,694834976	0,191863076
574	F1MNT1	Nucleoporin 155	NUP155	1354	151087	7	7,754000276	446493,8456	414630,5443	1,076847453	0,975750228
575	F1MP86	Tetraspanin	TSPAN8	238	26208	8	27,30999887	420864,3039	392155,1321	1,073208711	0,686434921
576	F1MPF5	Sperm acrosome developmental regulator	SPACDR	200	22379	13	50,49999952	1911516,071	856449,5844	2,23190729	0,210140429
577	F1MPK4	3-hydroxyisobutyryl-CoA hydrolase (HIB-CoA hydrolase) (HIBYL-CoA-H) (EC 3.1.2.4) (3	HIBCH	386	43478	2	9,066999704	9227,932619	15671,2102	0,588846203	0,403364989
578	F1MQE8	Synaptojanin 2 binding protein	SYNJ2BP	145	15826	1	8,275999874	22112,80028	27125,92406	0,815190673	0,644203165
579	P12820	Angiotensin-converting enzyme (ACE) (EC 3.4.15.1) (Dipeptidyl carboxypeptidase I) (K	ACE DCP1	1306	150097	13	10,18000022	1830538,32	1462584,76	1,251577597	0,50928674
580	F1MQX0	Acetyl-coenzyme A synthetase (EC 6.2.1.1)	ACSS1	675	74294	22	37,92999983	51449,59988	63281,06428	0,8130331	0,484595034
581	F1MR35	Angiotensin-converting enzyme (EC 3.4.-.-)		747	86747	12	19,81000006	1942188,287	1400396,643	1,386884421	0,04467807
582	F1MR53	Coiled-coil domain containing 27		305	36069	3	12,79000044	61593,27677	47806,321	1,2883919	0,300356268
583	F1MRQ2	Chromosome 3 C1orf185 homolog	C3H1orf185	225	25679	2	10,58000028	27971,31067	24140,96076	1,15866601	0,99108826
584	F1MS25	NADH dehydrogenase [ubiquinone] iron-sulfur protein 3, mitochondrial (Complex I-3	NDUFS3	266	30240	17	46,9900012	1056990,423	1114029,058	0,948799689	0,715337856
585	F1MSR1	Peptidase S1 domain-containing protein		471	52558	1	4,746999964	32273,2235	30904,07558	1,044303151	0,895983532
586	F1MSV5	glycerol kinase (EC 2.7.1.30)	GK2	554	60868	41	51,6200006	2329025,196	2841825,46	0,819552512	0,088790258
587	F1MUB8	Spermatosis and centriole associated 1	SPATC1	419	45166	11	26,26999915	4120486,017	3179261,936	1,296051127	0,149930287
588	F1MUV1	fatty acid amide hydrolase (EC 3.5.1.99)	FAAH	579	63025	7	19,16999966	615454,4627	517504,0505	1,189274677	0,291079228
589	F1MUZ9	60 kDa heat shock protein, mitochondrial (EC 5.6.1.7) (60 kDa chaperonin) (Chapero	HSPD1	541	57682	43	64,74999785	3124318,163	3947238,961	0,791519894	0,080450779
590	F1MVL2	Short-chain specific acyl-CoA dehydrogenase, mitochondrial (EC 1.3.8.1) (Butyryl-Co	ACADS	414	44700	12	32,51999915	882269,1903	923909,1256	0,954930703	0,975750228
591	F1MW14	RAN guanine nucleotide release factor	RANGRF	186	20714	5	61,83000207	75052,77899	58810,60395	1,276177661	0,717762839
592	F1MWD3	T-complex protein 1 subunit epsilon (CCT-epsilon)	CCT5	541	59601	6	16,64000005	171695,1762	131309,2501	1,307563451	0,126671924
593	F1MWF0	Huntingtin interacting protein 1	HIP1	1038	116622	4	5,110999942	91546,78008	77177,15746	1,186190099	0,454341614
594	F1MWN8	Cilia and flagella associated protein 58	CFAP58	873	103738	7	10,37999988	54977,06221	41893,13066	1,312316873	0,225338155
595	F1MWR3	Electron transfer flavoprotein subunit alpha (Alpha-ETF)	ETFA	333	34957	16	64,25999999	8088,860315	9744,925517	0,830058711	0,829647132
596	F1MWT0	alpha-mannosidase (EC 3.2.1.24)	MAN2C1	1008	112316	2	2,212000079	110272,3052	99107,25365	1,11265625	0,694005862
597	F1MWX4	Histone deacetylase 11	HDAC11	268	30573	2	6,233999878	127363,1401	127922,675	0,995625991	0,980045196
598	F1MWY9	Enoyl-CoA delta isomerase 2	ECI2	393	42772	18	42,23999977	1088116,634	1240586,991	0,877098214	0,332251049
599	F1MX05	Saccharopine dehydrogenase-like oxidoreductase	SCCPDH	429	47316	65	60,83999872	10708984,99	9119234,266	1,174329409	0,451384763
600	F1MX68	Carboxypeptidase vitellogenic like	CPVL	480	54524	13	23,95000011	1876400,752	1350084,51	1,389839479	0,03202207
601	F1MX88	Solute carrier family 25 member 13	SLC25A13	679	75042	4	8,100000024	25506,84165	25376,56748	1,00513364	0,984853837
602	F1MXX0	MICOS complex subunit MIC60 (Mitofilin)	IMMT	760	84097	18	30,30999899	875406,5937	704175,3942	1,243165553	0,11028276
603	F1MXX5	Nardilysin convertase				3	3,10399998	81676,99158	80413,80422	1,015708588	0,855554095
604	F1MXY8	Citrate transport protein	SLC25A1	311	33780	7	31,51000142	774597,5241	767001,3114	1,009903781	0,92558903
605	F1MY12	NTR domain-containing protein	LOC112442231	243	28493	1	4,526999965	17984,87661	12494,11182	1,439468196	0,259862279
606	F1MY24	Chaperonin containing TCP1 subunit 8 like 2	CCT8L2	534	56607	6	20,03999949	301966,844	234221,3193	1,289237226	0,186576931
607	F1MY32	Lymphocyte antigen 6 complex locus protein G5c	LY6G5C	140	16094	3	26,24000013	91137,09769	84023,01775	1,084668227	0,975027051
608	F1MY93	Leucine rich repeat containing 34	LRRC34	464	50914	1	2,661000006	52897,21331	52946,04234	0,999077759	0,975027051
609	F1MYA6	IZUMO family member 4	IZUMO4	207	23711	30	45,07000148	10419810,14	8549801,12	1,218719593	0,106356656
610	F1MYE8	Acyl-CoA dehydrogenase family member 9	ACAD9	594	65165	18	33,66999924	706713,3653	885305,001	0,798271064	0,228329123
611	F1MYG0	Ornithine aminotransferase (EC 2.6.1.13)	OAT	439	48118	9	29,37999964	129318,3209	173689,101	0,744539065	0,398756376
612	F1MYH5	A-kinase anchoring protein 4	AKAP4	831	92103	690	97,17000127	64964873,61	76187556,76	0,852696639	0,801100048
613	F1MZ38	Succinate--CoA ligase [ADP/GDP-forming] subunit alpha, mitochondrial (EC 6.2.1.4) (	SUCLG1	346	36139	14	29,19000089	1217520,907	1388508,085	0,876855468	0,427628483
614	F1MZ78	Leucine rich repeat and coiled-coil centrosomal protein 1	LRRCC1	1027	118790	1	1,168000046	75106,70035	67303,67897	1,115937516	0,840385936
615	A5PJD8	Protein SPMIP9 (Sperm microtubule inner protein 9) (Testis-expressed sequence 37 p	SPMIP9 TEX37 TSC21	180	20322	4	38,33000064	135192,95	163876,4525	0,824968737	0,362798124
616	F1MZD5	Dehydrogenase/reductase 4	DHRS4	279	29454	2	8,961000293	165365,553	198830,2974	0,831691926	0,48786839
617	F1MZG1	Spermatosis associated 6	SPATA6	488	56206	4	10,66000015	115272,8364	103532,0411	1,113402529	0,480735231
618	F1MZJ1	Tektin bundle interacting protein 1	TEKTIP1	306	34641	9	52,88000107	672044,2149	624693,8418	1,075797727	0,749494315
619	F1MZR7	Spermatid maturation 1	SPEM1	313	35314	4	24,27999973	81887,22512	26922,81285	3,041555337	0,004887624
620	F1N058	Uncharacterized protein		212	23978	64	88,67999911	16882153,77	14437218,78	1,169349445	0,385818207
621	F1N124	Leucine rich repeat containing 23	LRRC23	342	39611	5	14,32999969	112046,2457	84411,50529	1,327381206	0,204107047
622	F1N191	Interleukin 4 induced 1	IL4I1	589	65759	80	74,57000017	38857544,62	40237576,57	0,965702906	0,798663492
623	F1N1E1	Stabilizer of axonemal microtubules 4	SAXO4	428	47685	2	5,373999849	164526,3802	223400,2194	0,736464721	0,172555489
624	F1N1M7	Carnitine O-palmitoyltransferase 2, mitochondrial (EC 2.3.1.21) (Carnitine palmitoyl	CPT2	658	74455	5	13,36999983	49925,19362	60309,32152	0,827818857	0,373978461
625	F1N1W5	Nitrilase 1	NIT1	244	26701	1	5,488000065	12901,81574	15312,36358	0,842575065	0,855554095
626	F1N206	Dihydrolipoyl dehydrogenase, mitochondrial (EC 1.8.1.4) (Dihydrolipoamide dehydr	DLD	509	54187	33	50,09999871	2169697,947	2426815,114	0,894051605	0,351036391
627	F1N2F2	Phosphoglycerate mutase (EC 5.4.2.11) (EC 5.4.2.4)	PGAM2	253	28699	12	45,05999982	516877,1728	424036,5581	1,218944836	0,306796398
628	F1N2S7	non-specific serine/threonine protein kinase (EC 2.7.11.1)	TSSK6	273	30211	6	25,63999891	1165663,926	1209821,915	0,963500422	0,946638787
629	F1N2V1	Golgi associated RAB2 interactor family member 2	GARIN2	393	43848	7	21,90999985	681807,5347	528039,1157	1,291206493	0,258073581
630	F1N310	NADH-cytochrome b5 reductase (EC 1.6.2.2)	CYB5R2	235	26708	1	4,029000178	8317,982058	7450,050596	1,116500076	0,749494315
631	F1N338	3-hydroxyacyl-CoA dehydrogenase (EC 1.1.1.35)	HADH	314	34391	4	16,24000072	822967,5911	866066,5416	0,950235982	0,860410144
632	F1N343	Coiled-coil domain containing 136	CCDC136	1204	138878	39	37,72000074	1423036,128	1241208,206	1,146492684	0,447720839
633	F1N369	Zona pellucida binding protein	ZPBP	323	36800	55	55,43000102	44397817,01	37461026,81	1,18517352	0,318490948
634	F1N3A8	Cilia-and flagella-associated protein 96	CFAP96	309	34499	3	17,14999974	33802,11134	26722,33027	1,264938761	0,186310882
635	F1N3F1	LETM1 domain containing 1	LETMD1	270	31154	1	3,333000094	41867,71351	38255,56875	1,09442141	0,952134592
636	F1N3G6	Phospholipid-transporting ATPase (EC 7.6.2.1)	ATP11C	1119	128315	3	4,11100015	33510,39133	44587,56617	0,75156359	0,615908187

637	F1N3Q8	MIER family member 2	MIER2	559	61072	1	1,425999962	255006,6723	123542,5992	2,064119373	0,625236702
638	F1N3R4	Mitochondrial proton/calcium exchanger protein (Electroneutral mitochondrial K(+	LETM1	786	87860	6	10,05000025	105191,2997	110056,4155	0,955794347	0,748316576
639	F1N3X1	IQ motif containing F5		197	23660	2	6,215000153	130648,6715	149037,9027	0,876613728	0,446878302
640	F1N402	Centrosomal protein of 44 kDa	CEP44	396	44944	1	7,272999734	18286,47122	19190,32296	0,95290065	0,836095172
641	F1N427	Septin	SEPTIN12	361	41134	8	36,84000075	127465,9423	80023,13187	1,592863705	0,00502487
642	F1N4A1	Armadillo repeat containing 12	ARMC12	338	38134	22	71,89000249	4121168,505	4864673,948	0,847162328	0,191863076
643	F1N4M3	Pyruvate dehydrogenase phosphatase regulatory subunit	PDPR	879	98940	5	6,834000349	173751,4433	206729,2181	0,840478404	0,454797657
644	F1N5C9	Radial spoke head 10 homolog B (Chlamydomonas)	RSPH10B	871	99777	2	2,985000052	58656,74458	54924,36875	1,067954824	0,99108826
645	F1N5R7	Dynein axonemal heavy chain 7	DNAH7	4024	461307	33	11,20999977	420773,5997	388048,5049	1,084332485	0,571258346
646	P11180	Dihydrolipoyllysine-residue acetyltransferase component of pyruvate dehydrogenase	DLAT	647	69067	18	39,71999884	6251045,56	7962953,405	0,785015966	0,166978533
647	F1N6A0	Isochorismatase domain containing 2	ISOC2	219	24032	11	53,43000293	396316,0684	436338,3242	0,90827701	0,794308208
648	F1N6I4	NADP-dependent oxidoreductase domain-containing protein	AKR7A2	369	40242	1	4,490999877	98618,48643	99123,66137	0,994903589	0,727036926
649	F1N6K8	Uncharacterized protein	LOC789612	275	29927	18	60,72999835	2162513,173	1786269,625	1,210630883	0,271770823
650	F1N6M5	Coiled-coil domain containing 183	CCDC183	534	62388	2	4,493999854	23478,54658	20274,34965	1,158041909	0,87421943
651	F1N7K1	Ropporin-1-like protein	ROPN1L	218	24422	10	46,32999897	1443136,314	1059905,566	1,361570654	0,017424933
652	F1N7P3	EF-hand domain-containing protein		495	55523	3	8,889000118	276638,8491	223978,4876	1,235113479	0,489639155
653	F1N7X7	Golgi associated RAB2B interactor family member 3	GARIN3	587	62663	35	49,90999997	2568000,882	3037053,642	0,845556643	0,424620554
654	F2Z4D5	Ras-related protein Rab-11A (EC 3.6.5.2)	RAB11A	216	24394	1	5,093000084	33566,58531	26186,58447	1,281823727	0,423145279
655	F6QGE6	Acyl-CoA thioesterase 13	ACOT13	236	25627	3	14,8300007	46700,87191	57926,6317	0,806207275	0,508178477
656	F6QJG7	Zona pellucida binding protein 2	ZPBP2	326	37409	13	38,96000087	351634,786	279425,9782	1,258418377	0,149930287
657	F6QQ46	Chromosome 10 C15orf48 homolog	C10H15orf48	179	20048	11	45,44999897	2102061,458	2461522,397	0,853968041	0,282121058
658	F6R6Q1	FAD synthase (EC 2.7.7.2) (FAD pyrophosphorylase) (FMN adenylyltransferase) (Flavin	FLAD1	490	54273	2	7,142999768	52732,29262	60009,35363	0,878734554	0,466326307
659	F6RFP6	Basigin	BSG	320	34897	5	16,56000018	429117,5003	462155,1757	0,928513891	0,748316576
660	F6RJC2	Cilia- and flagella-associated protein 161	CFAP161	321	36470	7	26,48000121	432516,8218	441246,7569	0,980215299	0,938963059
661	F6RP72	Tubulin alpha chain	LOC100295712 TUBA3E	458	50886	138	74,01999831	65296645,01	73119643,51	0,893010987	0,624466646
662	G1K1H0	Disintegrin and metalloproteinase domain-containing protein 2 (Fertilin subunit bet	ADAM2	745	83127	37	20,66999972	644959,141	866799,8468	0,744069284	0,102419752
663	G3MWG4	isoleucine--tRNA ligase (EC 6.1.1.5) (Isoleucyl-tRNA synthetase)	IARS2	1012	113608	10	11,06999964	184889,4196	248162,9586	0,745032299	0,07942107
664	G3MY76	Cylicin 1	CYLC1	661	74119	33	30,07999957	1473981,853	474805,8128	3,104388811	0,001852239
665	G3MY97	Golgi associated RAB2 interactor protein-like Rab2B-binding domain-containing pro		856	94610	2	3,652000055	79917,79171	84081,9587	0,950474905	0,760141892
666	G3MZ01	Profilin		153	17049	1	7,842999697	97273,5906	98712,42901	0,985423939	0,975027051
667	G3MZM7	Uncharacterized protein	LOC132343038	520	55964	9	24,04000014	159579,2056	113512,259	1,405832347	0,195711262
668	G3MZM8	vitamin-K-epoxide reductase (warfarin-sensitive) (EC 1.17.4.4)	VKORC1L1	301	31826	2	11,93000004	347825,2043	264437,2673	1,315341093	0,126696309
669	G3MZS1	deleted				1	34,47999954	17999,54105	18946,65854	0,950011371	0,857569767
670	G3MZX2	Proline rich 30	PRR30	303	31236	9	34,16999876	37085,71635	29009,21667	1,278411505	0,26390167
671	G3MZZ7	Family with sequence similarity 170 member B	FAM170B	289	32536	1	4,49800007	4120,547836	3402,889881	1,210896614	0,827214415
672	G3N0F7	Testis expressed 50	TEX50	179	20784	5	20,66999972	94537,1541	59945,84944	1,577042531	0,058738224
673	G3N0V2	Keratin, type II cytoskeletal 1 (Cytokeratin-1) (Keratin-1) (Type-II keratin Kb1)	KRT1	606	63165	18	16,33999944	947403,6345	1383167,5	0,684952209	0,428559287
674	G3N136	Testis specific serine kinase 2	TSSK2	258	26780	13	39,10999894	354823,1401	346429,1054	1,024230166	0,977014723
675	G3N284	Actin related protein T1 O				9	28,65000069	104290,4857	97632,18321	1,068197824	0,855554095
676	G3N2D2	non-specific serine/threonine protein kinase (EC 2.7.11.1)	TSSK1 TSSK1B	367	41541	5	18,79999936	22296,34432	28920,76961	0,770945747	0,519920943
677	G3N2I2	Chromosome 11 C2orf81 homolog	C11H2orf81	606	65873	1	2,639999986	37440,57677	42720,61445	0,87640539	0,625168767
678	G3N3B6	Coiled-coil domain containing 188	CCDC188	502	53674	5	19,92000043	16578,01181	18438,39985	0,899102522	0,875767994
679	G3X6A7	Outer dense fiber protein 3				11	48,42999876	2003540,85	1480509,661	1,353277795	0,145447082
680	G3X6E2	Ciliary microtubule inner protein 2A	CIMIP2A FAM166A	320	36204	32	82,81000257	3055915,069	3263903,996	0,936276028	0,658082286
681	G3X7X2	Solute carrier family 25 member 32	SLC25A32	317	35618	3	10,72999984	297022,8392	275839,2434	1,076796889	0,980045196
682	G3X800	Disintegrin and metalloproteinase domain-containing protein 20-like		722	82124	18	20,85999995	619512,2535	524244,9386	1,181722908	0,615629458
683	G3X807	Histone H4		82	9262	6	41,83999896	5139572,604	4899350,932	1,049031326	0,920965712
684	G3X8G1	Sperm microtubule inner protein 1	SPMIP1 ATP6V1FNB	176	20248	3	18,17999929	72706,14515	64274,42373	1,131183151	0,625243734
685	G5E519	Coiled-coil domain-containing protein 39	CCDC39	941	109998	2	2,125000022	17300,69344	20620,48603	0,839005124	0,939494502
686	G5E521	NAD-dependent protein deacetylase (EC 2.3.1.286)	SIRT3	333	36992	7	30,03000021	380567,6058	334854,1475	1,136517522	0,65224982
687	G5E531	T-complex protein 1 subunit alpha (CCT-alpha)	TCP1	556	60222	13	22,48000056	523783,8945	420612,1712	1,245289439	0,126671924
688	G5E5C6	Acrosin (EC 3.4.21.10)	ACR	414	45494	64	56,04000092	75656765,79	51021359,29	1,482844966	0,000733725
689	G5E6S5	Coiled-coil domain containing 159	CCDC159	297	33958	5	22,25999981	162881,8392	178360,4777	0,913217106	0,629265378
690	G8CY12	Beta-defensin		89	9374	5	26,96999907	85770,58704	85012,05847	1,0089226	0,995935621
691	I6NUJ4	Choline transporter-like protein	SLC44A5	717	81886	4	10,59999987	98074,08304	90599,48126	1,082501596	0,798929132
692	M0QVZ6	IF rod domain-containing protein		606	64982	10	11,45000011	1258725,101	2290869,353	0,549453027	0,177243867
693	M4MFL5	Trypsin-like serine protease-like protein		301	32668	1	8,305999637	27404,40495	12848,7419	2,132847338	0,191863076
694	M5FHL5	NADH dehydrogenase [ubiquinone] 1 beta subcomplex subunit 10				4	29,55000103	177345,4509	210429,3096	0,842779227	0,508178477
695	M5FJY9	Phosphoglycolate phosphatase				3	13,07999939	81025,74991	67562,37315	1,199273296	0,961830278
696	M5FK65	Protease, serine, 21-like				1	4,947999865	17302,25315	12568,54461	1,376631399	0,686434921
697	M5FK83	CG2446-like				2	17,6699996	164253,041	155512,0879	1,056207547	0,781648136
698	M5FKE0	Tektin				41	71,59000039	47213,44511	59014,48689	0,800031443	0,606839298
699	M5FMT6	Hydroxyacylglutathione hydrolase, mitochondrial				5	23,3799994	15870,20707	22620,01332	0,701600253	0,191863076
700	N0E4A8	Casein kinase II subunit beta (Phosvitin)	Csnk2b-Ly6g5b	233	26239	3	25,31999946	60349,74367	54450,51997	1,108340998	0,749494315

701	O77779	Fertilin alpha	ADAM 1	812	89905	20	21,06000036	818363,6372	863363,2924	0,947878656	0,694005862
702	Q05AT5	HIG1 domain family, member 2A	HIGD2A	106	11575	1	30,18999994	24053,09145	24477,20969	0,982672934	0,915538761
703	Q08DM3	Malic enzyme	ME2	584	65413	2	6,848999858	14545,27403	11355,50923	1,280900199	0,285608246
704	Q08DN5	Carnitine acetyltransferase	MGC142781 CRAT	626	71017	14	28,11999917	539314,1922	670276,1199	0,804614958	0,088797393
705	Q0II40	Dual specificity phosphatase 21 (Similar to Dual specificity protein phosphatase 18 (I	DUSP21 LOC538872	196	22052	6	23,46999943	169906,4014	214158,9016	0,793366048	0,258073581
706	Q0IIB9	Uncharacterized protein LOC777692	LOC777692	106	12606	1	16,04000032	22435,80611	29587,87855	0,758276944	0,263093664
707	Q0V8B1	Protein Flattop (Cilia- and flagella-associated protein 126)	C1orf192	216	23381	1	4,630000144	83426,97215	88301,53401	0,944796408	0,723696372
708	Q0VCS8	glutathione transferase (EC 2.5.1.18)	GSTT3	239	27277	2	12,54999936	165524,099	158426,4289	1,044801048	0,975027051
709	Q0VCU3	Cathepsin F	CTSF	460	50893	17	31,95999861	11955408,3	8899763,966	1,343339929	0,05146089
710	Q148D3	Fumarate hydratase, mitochondrial (EC 4.2.1.2)	FH	510	54691	30	54,50999737	2710451,114	3468788,912	0,781382547	0,05146089
711	Q148I4	Acyl-CoA thioesterase 7	ACOT7	338	37368	2	7,987999916	89428,15843	67643,261	1,322055695	0,159205073
712	Q148J8	Isocitrate dehydrogenase [NAD] subunit, mitochondrial	IDH3A	366	39698	10	30,86999953	946323,258	1117816,773	0,84658173	0,370087102
713	Q17QF5	Protein kinase, cAMP-dependent, regulatory, type I, beta	PRKAR1B	381	43032	4	15,21999985	84383,12488	52527,10334	1,606468271	0,040206821
714	Q17QY3	Nipsnap homolog 3A (C. elegans)	NIPSNAP3A	247	28540	1	4,857999831	7943,946421	6898,840861	1,151490023	0,95403064
715	Q1JPA2	Elongation factor 1-gamma (eEF-1B gamma)	EEF1G	439	50231	4	11,15999967	158020,2222	115441,2253	1,368837014	0,857569767
716	Q1JPF2	NADH dehydrogenase [ubiquinone] 1 alpha subcomplex subunit 9, mitochondrial (C	NDUFA9	369	41660	12	39,84000087	1126770,028	1009982,232	1,115633516	0,444776105
717	Q1LZ81	PDCD6 protein	PDCD6	189	21737	3	26,98000073	45346,64165	41293,95089	1,09814248	0,748316576
718	Q1RMN5	Spermatogenesis associated 3 (Spermatosis associated 3)	SPATA3	230	24393	2	11,2999998	311227,9105	285823,6197	1,088881006	0,900890629
719	Q1RMP3	CutA divalent cation tolerance homolog (E. coli)	CUTA	153	16347	4	30,72000146	105716,0244	73154,5035	1,44510617	0,137302261
720	Q24JZ7	Succinyl-CoA:3-ketoacid-coenzyme A transferase (EC 2.8.3.5)	OXCT1	520	56453	15	29,42000031	602442,9594	772500,6729	0,779860757	0,05146089
721	Q29RT2	Hypothetical LOC540061	LOC540061	242	27071	2	7,438000292	20090,77606	18298,60269	1,09794045	0,749494315
722	Q29RT3	MS4A13 protein	NYD-SP21	861	97385	3	4,994000122	44234,83495	47343,83157	0,934331538	0,900890629
723	Q29RU5	Rhabdoid tumor deletion region gene 1	RTDR1	379	41493	6	22,95999974	144859,2121	111342,9612	1,301018138	0,195027592
724	Q2HJI1	Succinate dehydrogenase [ubiquinone] flavoprotein subunit, mitochondrial (EC 1.3.!	SDHA	678	74407	35	45,87000012	2883236,037	3483308,294	0,827729214	0,303281271
725	Q2KIC7	Serine/threonine-protein phosphatase (EC 3.1.3.16)	PPP6C	305	35144	5	23,61000031	43740,6945	41803,71883	1,046335009	0,935160425
726	Q2KII5	Histone H2B	HIST1H2BI H2BC5	126	13936	5	33,32999945	3605664,409	2966249,887	1,215563269	0,972332714
727	Q2KIK5	LOC767871 protein	LOC767871	392	43229	7	15,04999995	148297,0094	114871,3201	1,290983766	0,26390167
728	Q2KIV7	inorganic diphosphatase (EC 3.6.1.1)	PPA2	327	36963	3	13,45999986	17580,18533	14272,74713	1,231731017	0,539023906
729	Q2KIV8	Glutathione S-transferase (EC 2.5.1.18)	GSTM3	225	26850	105	91,56000018	58389771,12	60391522,01	0,966853776	0,952134592
730	Q2KIZ0	TSBP protein	TSBP	573	62599	2	6,806000322	25898,16667	18407,35597	1,406946587	0,274006077
731	Q2NKS5	Uncharacterized protein MGC137053	MGC137053	204	23462	3	17,64999926	164795,5551	145486,0793	1,132723872	0,644203165
732	Q2NKS8	peptidylprolyl isomerase (EC 5.2.1.8)	LOC526524	108	11945	3	41,67000055	97758,37257	81071,91959	1,205822843	0,26472689
733	Q2NKT6	Ciliary microtubule inner protein 3 (Uncharacterized protein MGC137036)	MGC137036 CIMIP3	112	12953	1	14,2900005	48321,89181	43371,54248	1,114138189	0,984853837
734	Q2NKZ3	ADAM metallopeptidase domain 32	ADAM32	747	82546	22	19,14000064	9457280,851	7609303,652	1,242857597	0,249343849
735	Q2NKZ8	glycerol kinase (EC 2.7.1.30)	LOC538702	553	60602	43	64,74000216	9728768,578	12405470	0,784232163	0,016045645
736	Q2NL07	Farnesyl pyrophosphate synthase ((2E,6E)-farnesyl diphosphate synthase) (Dimethyla	FDPS	421	48333	11	21,85000032	38405,89622	45688,11647	0,840610189	0,377095823
737	Q2T9Y3	Pyruvate dehydrogenase E1 component subunit alpha (EC 1.2.4.1)	PDHA2	391	43292	22	49,86999929	1126408,829	1454711,612	0,774317617	0,145447082
738	Q2TA15	Chromosome 15 open reading frame 23 ortholog (Kinetochore localized astrin (SPAG	KNSTRN C10H15orf23	234	25676	1	9,402000159	40444,5855	22165,49032	1,824664599	0,276175072
739	Q2TA22	Long-chain-fatty-acid--CoA ligase (EC 6.2.1.15) (EC 6.2.1.3) (Acyl-CoA synthetase) (Lor	ACSL6	697	78043	15	31,56000078	614177,0246	418059,4292	1,469114154	0,017424933
740	Q2TA28	Signal-regulatory protein delta	SIRPD	286	31467	7	28,31999958	212801,9864	164278,8627	1,295370463	0,204107047
741	Q2TA33	Membrane cofactor protein	LOC616002	397	45796	8	20,14999986	340647,3326	272958,967	1,247980004	0,373133287
742	Q2TBH3	Actin related protein M1	ARPM1	368	40943	26	48,64000082	4184543,478	4655037,426	0,898927999	0,315593909
743	Q2TBP9	Coilin	COIL	571	63122	3	7,705999911	76906,53275	50195,89619	1,532127895	0,26390167
744	Q2TBR1	5'-nucleotidase, cytosolic IB	NT5C1B	564	64011	90	68,79000068	4242585,574	4321140,732	0,981820736	0,900919909
745	Q2TBS5	Serine peptidase inhibitor, Kazal type 2 (Acrosin-trypsin inhibitor)	SPINK2	84	9391	3	53,57000232	536118,3013	556561,0076	0,963269604	0,552042161
746	Q2TBW4	Polycystic kidney disease 2-like 2	PKD2L2	600	71610	1	1,999999955	47139,20329	54269,0757	0,868619977	0,566781527
747	Q2YDK3	Hyaluronidase (EC 3.2.1.35)	SPAM1	553	62299	49	52,25999951	14330153,61	9175382,566	1,561804482	0,01594206
748	Q2YDK7	Thioredoxin domain containing 3 (Spermatozoa)	TXNDC3	410	46919	7	21,95000052	240914,9394	196498,8441	1,226037438	0,061953186
749	Q2YDL8	Uncharacterized protein LOC780846	LOC780846	243	27324	7	27,97999978	137881,1809	113292,0973	1,217041472	0,840385936
750	Q2YDP2	Sad1 and UNC84 domain containing 5 (Sperm associated antigen 4-like)	SUN5 SPAG4L	372	42629	8	34,40999985	11954,90657	10644,43177	1,123113646	0,489639155
751	Q32KP4	Abhydrolase domain containing 16B (Chromosome 20 open reading frame 135 orthc	ABHD16B C13H20orf135	470	52624	4	11,28000021	389774,2036	347206,518	1,12260048	0,491533511
752	Q32KP8	Serine peptidase inhibitor-like, with Kunitz and WAP domains 1 (Eppin)	SPINLW1	134	15093	2	22,39000052	50394,9032	26893,07025	1,873899214	0,457406445
753	Q32KR0	TBC1 domain family, member 21	TBC1D21	299	34883	1	3,678999841	23982,12349	14699,015	1,631546296	0,233339863
754	Q32KR2	Acrosomal vesicle protein 1	ACRV1	273	28953	26	23,44000041	344079,3143	295100,1023	1,165974907	0,727036926
755	Q32KR7	Hypothetical LOC539526	LOC539526	477	54623	20	46,11999989	775911,0345	724812,3048	1,070499258	0,727036926
756	Q32KS3	F-actin-capping protein subunit alpha	CAPZA3	299	35066	67	84,61999893	18618968,68	13844697,31	1,344844763	0,149930287
757	Q32L54	Sperm associated antigen 6	SPAG6	509	55443	24	53,82999778	1818344,494	1724234,979	1,054580446	0,692594105
758	Q32L61	Calcium binding tyrosine-(Y)-phosphorylation regulated	CABYR	454	48564	29	51,09999776	1208386,665	1546669,603	0,781282998	0,126671924
759	Q32L64	Similar to DnaJ (Hsp40) homolog, subfamily B, member 3	LOC528549	244	26915	9	28,2799989	187218,1705	210116,9034	0,891019083	0,708414398
760	Q32L74	Aquaporin 7	AQP7	330	35948	7	23,93999994	699137,1604	677195,3512	1,032401004	0,759626475
761	Q32L87	DDB1 and CUL4 associated factor 7 (WD repeat domain 68)	DCAF7 WDR68	342	38926	1	4,385999963	29027,64466	33183,44036	0,874762964	0,537436684
762	Q32LI7	Ciliary microtubule inner protein 4 (Uncharacterized protein MGC133880)	CIMIP4 MGC133880 TEX3:	272	30275	1	4,411999881	8751,305499	11524,51555	0,759364284	0,696102603
763	Q32LN4	Cilia- and flagella-associated protein 45	CFAP45 CCDC19	549	65666	6	13,66000026	206490,3457	167744,1078	1,230984196	0,282430005
764	Q32LP8	Cysteine-rich secretory protein 2	CRISP2	244	27143	6	22,13000059	93079,07149	85304,73342	1,091136069	0,829647132

765	Q32P61	Histone H2A	LOC523631	117	13150	11	76,06999874	323756,3528	427355,1491	0,757581495	0,418068945
766	Q32PA1	CD59 glycoprotein (MAC-inhibitory protein) (Membrane attack complex inhibition f	CD59	121	13663	2	20,65999955	50078,02785	63571,3163	0,787745649	0,428925016
767	Q32S21	FabG-like protein (Hydroxysteroid (17-beta) dehydrogenase 8)	HKE6 FABGL	259	26468	3	17,75999963	148448,7191	188310,1912	0,788320155	0,4399745
768	Q3MHX0	sulfite oxidase (EC 1.8.3.1)	SUOX	547	60501	8	25,96000135	602513,2105	803382,4023	0,749970635	0,284967809
769	Q3SZ00	enoyl-CoA hydratase (EC 4.2.1.17)	HADHA	763	83249	24	42,3299998	3906840,855	4228639,74	0,923900142	0,615629458
770	Q3SZ15	Eukaryotic translation elongation factor 1 epsilon 1	EEF1E1	174	19757	2	14,37000036	75243,24255	64583,59151	1,165052001	0,508178477
771	Q3SZA4	Solute carrier family 25 (Carnitine/acylcarnitine translocase), member 20 (Solute car	SLC25A20	301	32928	2	8,637999743	165333,6479	189165,8027	0,874014465	0,454797657
772	Q3SZK3	Growth hormone inducible transmembrane protein	GHITM	345	37029	4	7,825999707	341774,4841	405222,4934	0,843424266	0,344056033
773	Q3SZW9	DnaJ (Hsp40) related, subfamily B, member 13 (DnaJ heat shock protein family (Hsp4	DNAJB13	316	36077	13	57,27999806	907288,0279	647895,7597	1,400361114	0,017680092
774	Q3SZX1	MGC127695 protein	MGC127695	78	8983	1	11,54000014	36181,72921	54261,14105	0,666807378	0,178188845
775	Q3T010	Phosphatidylethanolamine-binding protein 4	PEBP4	223	25146	15	44,38999891	1673464,523	1076790,661	1,554122433	0,053703612
776	Q3T034	Serine rich single-pass membrane protein 1 (Uncharacterized protein MGC127766)	SSMEM1 MGC127766	241	27622	5	27,79999971	13031,12085	8925,99506	1,459906796	0,241740802
777	Q3T0U3	Es1 protein	ES1	274	28699	6	35,76999903	375139,8264	432169,7207	0,8680382	0,607981468
778	Q3T0Z0	WAP four-disulfide core domain protein 2	WFDC2	123	12729	2	21,95000052	36916,167	21692,12156	1,70182372	0,126671924
779	Q3T172	ECH1 protein	ECH1	347	37572	3	11,52999997	95608,23843	104518,006	0,914753754	0,694005862
780	Q3ZBG1	Ras-related protein Rab-14 (EC 3.6.5.2)	RAB14	215	23897	3	23,26000035	24049,50431	17281,59645	1,391625154	0,727036926
781	Q3ZBY4	Fructose-bisphosphate aldolase (EC 4.1.2.13)	ALDOC	364	39382	21	25,2700001	58008,88757	43483,46296	1,334044798	0,731867033
782	Q4R0H2	Spermadhesin 2	spadh2 SPADH2	134	15221	4	43,27999949	217380,3293	48915,7134	4,44397749	1,13878E-05
783	Q58D70	Serine/threonine-protein phosphatase (EC 3.1.3.16)	PPP2CA	309	35580	1	11,96999997	9590,773383	8448,709597	1,135176121	0,712074596
784	Q58DM0	Isocitrate dehydrogenase [NAD] subunit, mitochondrial	IDH3G	392	42873	5	15,55999964	566877,3592	656826,0048	0,863055596	0,366989999
785	Q5E974	Deoxyguanosine kinase isoform a	DGUOK	277	32043	3	16,96999967	46333,10494	62560,42983	0,740613597	0,26390167
786	Q5RZ69	Sperm mitochondria-associated cysteine-rich protein	SMCP	103	10977	4	38,83000016	41264,75969	45510,79319	0,906702714	0,795757874
787	Q7JAT1	ATP synthase subunit a	ATP6 atp6 ATPase6	226	24788	1	6,194999814	7467,772877	6852,197574	1,089836187	0,855554095
788	Q7JAT2	ATP synthase protein 8	ATP8 atp8 ATPase8	66	7937	3	33,32999945	102294,8686	111641,6209	0,916278963	0,723696372
789	Q862J3	V-type proton ATPase subunit D (V-type proton ATPase subunit d) (Vacuolar proton p		165	18391	1	9,696999937	13050,16583	11920,98409	1,094722191	0,735430026
790	Q862K7	Dynein light chain		78	9252	24	79,49000001	1523948,889	1289500,455	1,181813378	0,601718366
791	Q8HZY1	Serine protease inhibitor clade E member 2 (Serine protease inhibitor-E2) (Serpin fam	SERPINE2	397	43877	5	17,63000041	148274,4396	23573,49046	6,289880571	2,68784E-06
792	Q8M444	Cytochrome c oxidase subunit 2	COX2	227	26020	24	30,84000051	2012853,954	2262284,316	0,889744026	0,579112446
793	Q9N273	Kappa-casein	CSN3	160	17879	1	6,25	16038,71815	24664,96884	0,650263062	0,606839298
794	U3GR08	NADH-ubiquinone oxidoreductase chain 4 (EC 7.1.1.2)	ND4	459	52113	2	5,011000112	7987,717942	6026,232417	1,325491184	0,384406391
795	U3GU87	Cytochrome c oxidase subunit 1 (EC 7.1.1.9)	COX1	514	57062	1	2,724000067	61072,77041	53755,84974	1,136113943	0,423145279
796	U3GXK6	Cytochrome c oxidase subunit 3	COX3	260	29876	2	5,384999886	48286,28423	49292,10938	0,979594601	0,857569767
797	U3GXM3	NADH-ubiquinone oxidoreductase chain 1 (EC 7.1.1.2)	ND1	318	35710	1	5,974999815	19654,20792	20201,24999	0,972920385	0,900890629
798	V6F7X8	deleted				1	6,751000136	17851,15843	22332,57446	0,799332762	0,96153267
799	V6F959	Oligoribonuclease, mitochondrial				6	47,11999893	1748342,987	2083176,794	0,839267696	0,51475685
800	V9VJQ3	ATP synthase subunit	SPEF2	1713	196868	1	0,817300007	27680,39145	18230,10323	1,518389177	0,05146089
801	W0UV03	Ribonuclease A C1	RAC1	150	16377	13	52,67000198	78504,14892	42701,14199	1,83845549	0,072184045
802	W0UVF3	Ribonuclease A M1	RAM1	146	17714	4	28,08000147	288112,9996	75584,1012	3,811820145	0,072184045
